# RBM6 splicing factor promotes homologous recombination repair of double-strand breaks and modulates sensitivity to chemotherapeutic drugs

**DOI:** 10.1093/nar/gkab976

**Published:** 2021-10-28

**Authors:** Feras E Machour, Enas R Abu-Zhayia, Samah W Awwad, Tirza Bidany-Mizrahi, Stefan Meinke, Laila A Bishara, Florian Heyd, Rami I Aqeilan, Nabieh Ayoub

**Affiliations:** Department of Biology, Technion - Israel Institute of Technology, Haifa 3200003, Israel; Department of Biology, Technion - Israel Institute of Technology, Haifa 3200003, Israel; Department of Biology, Technion - Israel Institute of Technology, Haifa 3200003, Israel; The Concern Foundation Laboratories, The Lautenberg Center for Immunology and Cancer Research, Department of Immunology and Cancer Research-IMRIC, Faculty of Medicine, Hebrew University of Jerusalem, Jerusalem 91120, Israel; Freie Universität Berlin, Institute of Chemistry and Biochemistry, Laboratory of RNA Biochemistry, Takustrasse 6, 14195 Berlin, Germany; Department of Biology, Technion - Israel Institute of Technology, Haifa 3200003, Israel; Freie Universität Berlin, Institute of Chemistry and Biochemistry, Laboratory of RNA Biochemistry, Takustrasse 6, 14195 Berlin, Germany; The Concern Foundation Laboratories, The Lautenberg Center for Immunology and Cancer Research, Department of Immunology and Cancer Research-IMRIC, Faculty of Medicine, Hebrew University of Jerusalem, Jerusalem 91120, Israel; Department of Biology, Technion - Israel Institute of Technology, Haifa 3200003, Israel

## Abstract

RNA-binding proteins regulate mRNA processing and translation and are often aberrantly expressed in cancer. The RNA-binding motif protein 6, RBM6, is a known alternative splicing factor that harbors tumor suppressor activity and is frequently mutated in human cancer. Here, we identify RBM6 as a novel regulator of homologous recombination (HR) repair of DNA double-strand breaks (DSBs). Mechanistically, we show that RBM6 regulates alternative splicing-coupled nonstop-decay of a positive HR regulator, Fe65/APBB1. RBM6 knockdown leads to a severe reduction in Fe65 protein levels and consequently impairs HR of DSBs. Accordingly, RBM6-deficient cancer cells are vulnerable to ATM and PARP inhibition and show remarkable sensitivity to cisplatin. Concordantly, cisplatin administration inhibits the growth of breast tumor devoid of RBM6 in mouse xenograft model. Furthermore, we observe that RBM6 protein is significantly lost in metastatic breast tumors compared with primary tumors, thus suggesting RBM6 as a potential therapeutic target of advanced breast cancer. Collectively, our results elucidate the link between the multifaceted roles of RBM6 in regulating alternative splicing and HR of DSBs that may contribute to tumorigenesis, and pave the way for new avenues of therapy for RBM6-deficient tumors.

## INTRODUCTION

Defective DNA damage repair leads to genomic instability, which is considered a common characteristic of cancer cells. To preserve genomic stability and cope with the enormous amount of DNA damage that our genome continuously experiences, cells have evolved a sophisticated cellular response, called DNA damage response (DDR). Among the different types of DNA lesions, double-strand breaks (DSBs) are considered a very cytotoxic form of DNA lesion, as a single unrepaired DSB can trigger cell death (1–3). Vertebrate cells use two main pathways to repair DSBs. The first is non-homologous end-joining (NHEJ), an error-prone process that functions throughout the cell cycle. The second is homologous recombination repair (HR), an error-free process that functions in late S and G2 phases, when an intact chromatid is available (4–8). Work from our lab and many others revealed a prevalent role of RNA binding proteins and pre-mRNA processing factors in DDR including DSB repair (9–23). Remarkably, splicing factors were shown to accumulate at DNA damage sites, suggesting that they may play a direct role in DDR beside their canonical function in pre-mRNA splicing (17,24–28). In addition, it was shown that DNA damage alters the expression and the activity of splicing factors, which subsequently may affect constitutive and alternative splicing of DDR factors (24,29–32). For example, splicing factors, such as MFAP1 and SRSF10, may contribute to genome stability by regulating the expression of known DDR genes. Concordantly, depletion of some SFs leads to abnormal splicing of DDR genes, thus disrupting the integrity of DNA damage repair and sensitizing cells to genotoxic agents (16,29,31). Therefore, the splicing machinery provides a basis for innovative-splicing-targeted cancer therapies (24,33–37).

RBM6 is an understudied protein that contains two RNA recognition motifs (RRMs), one Zinc finger domain, a G-patch and OCRE domain which were shown to be involved in alternative splicing regulation (38–41). RBM6 is a nuclear protein that forms foci which correspond to splicing speckles (42). In line with this, RBM6 regulates gene expression and alternative splicing (AS) of genes that are implicated in several cellular processes including tumorigenesis (43). In this regard, several lines of evidence suggest that RBM6 is a putative tumor suppressor gene: (i) RBM6 represses growth and progression of laryngocarcinoma (44). (ii) RBM6 is mapped to 3p21.3 region, which is frequently deleted in heavy smoker lung cancer and other tissues carcinomas (45,46). (iii) RBM6 is mutated in 2.4% of diverse human cancers from multiple origins (*n* = 10967 cases). Specifically, RBM6 mutations have been found in 1.5% of breast (*n* = 1084 cases) and 3.1% of lung cancers (*n* = 487 cases) (47–49). *(iv)* Insertional mutagenesis experiments support RBM6 as a cancer driver gene (50–55). In addition, a recent study identified RBM6 as a diagnostic biomarker for early detection of pancreatic cancer (56). While these observations identify RBM6 as an important factor in tumorigenesis in human patients, little is known about the underlying molecular mechanisms.

Herein, we describe a previously unrecognized role of RBM6 in regulating genomic integrity and DNA repair that can be associated with cancer progression. We identified a novel role of RBM6 in regulating HR of DSBs, at least in part by regulating mRNA expression levels of the HR factor Fe65 (also called APBB1). Interestingly, our data unprecedently show that RBM6 regulates alternative splicing coupled to nonstop-decay (AS-NSD) (57), a translation-dependent mRNA surveillance mechanism, of Fe65. Accordingly, RBM6-deficient cells are devoid of Fe65 protein, resulting in defective HR of DSBs that is amended upon the restoration of Fe65 expression. In line with this, RBM6 depletion renders cancer cells vulnerable to ATM and PARP inhibition and exhibits pronounced sensitivity to cisplatin. In agreement with this, cisplatin treatment severely sensitizes RBM6-deficient MDA-MB-231 breast cancer cells in a xenograft mouse model. Notably, RBM6 immunohistochemical staining of human tissue microarrays (TMA) shows that RBM6 protein level is significantly lost in human metastatic breast tumors when compared to primary tumors. This study proposes therefore that cisplatin can be therapeutically exploited to treat RBM6-deficient tumors.

## MATERIALS AND METHODS

### Transfections

Cell transfections with plasmid DNA or siRNA were performed using Polyethylenimine (PEI) and Lipofectamine RNAiMax, respectively, following the manufacturer's instructions. siRNAs used in this study are: RBM6 siRNA #63 (5′-CAAGUAGCAAGAAGAAAA-3′); RBM6 siRNA #66 (5′-GCAAGAAUUAAUAACCUA-3′); Rad51 siRNA (5′-GAGCUUGACAAACUACUUC-3′); PARP1 siRNA (5′-CCAUCGAUGUCAACUAUGA-3′); PELO siRNA (5′-UGCAGGCACCGUUAGGAUA-3′); Stealth RNAi negative control (Invitrogen).

### Cell irradiation and drug treatment

Cells were exposed to ionizing radiation (IR) using an X-ray machine (CellRad). Where indicated, cells were treated with ATM inhibitor (KU-60019), PARP inhibitor (Ku-0059436), Cisplatin, Caffeine and CHX.

### Endogenous homologous recombination assay

Homologous recombination repair assay was performed using Cas9-mediated knock-in of green fluorescent mClover into the first exon of the LMNA gene, as previously described (58). Briefly, cells were plated in 6-well plates and co-transfected with 1.6 μg pX330-LMNA-gRNA1 plasmid containing Cas9 and gRNA against LMNA exon 1 and 0.4 μg pCR2.1-CloverLamin plasmid containing HR donor sequence. In addition, 0.4 μg pDsRed-Monomer-C1 was included per transfection as a transfection control unless otherwise indicated in the figure legend. 12−16 h post transfection, culture medium was renewed and where indicated, ATMi (5 μM) or Caffeine (4 mM) were added. siRNA-mediated knockdown was performed 24 h prior to transfections. Seventy-two hours post-transfection, cells were collected and analyzed by flow cytometry. HR efficiency is percentage of DsRed-Monomer positive cells that express mClover.

### HR and NHEJ reporter assays

The efficiency of HR of DSBs was performed as previously described (13) in mock and RBM6-depleted U2OS‐HR‐ind cells. In brief, U2OS‐HR‐ind cells, which stably express cytoplasmic mCherry‐I‐SceI enzyme fused to glucocorticoid receptor (I‐SceI‐GR), were treated with 0.1 μM of dexamethasone (Dex) for 48 h. This treatment induces rapid entry of I‐SceI‐GR into the nucleus and generation of DSB at its recognition sequence within the reporter construct expressing GFP. Repairing the DSB by HR restores the integrity of the GFP gene. The number of GFP‐positive cells was determined using a BD LSRII. Data analysis was performed using FCS‐Express software and was based on at least 10 000 events. HeLa cells containing the plasmid pEJSSA stably integrated into their genome (59) were used to monitor the efficiency of NHEJ *in vivo* as previously described (13). In brief, control and NELF‐E‐depleted HeLa cells were co‐transfected with constructs expressing I‐SceI endonuclease and Red‐Monomer (MR) tag, and the percentage of GFP‐positive cells from the total number of red cells was determined by flow cytometry.

### Immunoprecipitation

HCT116 cells were transfected with plasmids expressing either Flag-RBM6, Myc-RNPSI or co-transfected with both plasmids. Thirty-six hours following transfection, cells were harvested and lysed with 10% NP-40 containing buffer supplemented with protease inhibitors. 1.5 mg proteins were immunoprecipitated using 1.5 μg flag antibody and subjected to western blot analysis.

### RNA immunoprecipitation using GFP-TRAP

HCT116 cells expressing either GFP only or GFP-RBM6 fusion were subject to GFP-TRAP assay as previously described (13). Briefly, GFP-TRAP beads (Chromotek) were blocked at 4°C for 2 h in immunoprecipitation (IP) buffer (50 mM HEPES, pH 7.4, 100 mM NaCl, 0.5% Nonidet P-40, 10 mM EDTA, 20 mM β-glycerophosphate) containing 5% BSA. Whole-cell extracts were prepared using Nonidet P-40–lysis buffer and precleared by centrifugation at 12 800 g for 15 min at 4°C. Four milligram of cell lysate was incubated for 2 h with GFP-TRAP beads. Next, Immunoprecipitated RNA was extracted using TRIzol reagent according to the manufacturer's instructions (Ambion) and purified RNA was resuspended in 20 μl RNase-free water. 10 μl of precipitated RNA or 1 μg of input RNA was then subject to RT-PCR using qScript cDNA Synthesis Kit (Quanta) with random primers followed by PCR reaction using Fe65 primers listed in the Key Resources table. PCR products were then resolved on agarose gel, stained with Ethidium Bromide, and imaged using GelDoc system (Invitrogen).

### UV cross-linking and immunoprecipitation (CLIP) followed by qPCR

MCF10A-HRas cells were UV cross-linked using 400 mJ/cm^2^ at 254 nm. Cells were then scraped and lysed in RIPA buffer (50 mM Tris (pH 7.5), 1 M sodium chloride, 1% NP-40, 0.1% sodium deoxycholate, 1 mM EDTA) supplemented with complete protease inhibitor cocktail (Calbiochem) and RNase inhibitor (MO314, NEB). Lysate was then immunoprecipitated with 2 μg of either IgG or RBM6 antibody overnight at 4°C. Immunoprecipitated RNA was then treated with RQ1 DNaseI (M6101 Promega) according to the manufacturer's instructions and eluted with Proteinase K before RNA extraction with Trizol reagent (Ambion). 10 μl of precipitated RNA or 1 μg of input RNA was then subject to RT-PCR using qScript cDNA Synthesis Kit (Quanta) with random primers followed by qPCR reaction in the Step‐One‐Plus real‐time PCR System (Applied Biosystems) using the indicated primers and the Fast SYBR Green Master mix (Applied Biosystems) with three technical repeats for each PCR. RNA levels from immunoprecipitated samples from three replicates was normalized to input RNA levels.

### Quantitative Immunofluorescence

Cells were seeded into 48-well plates in triplicates and grown for 24 h to reach density of ∼80%. Cells were then exposed to IR at the indicated timepoints before fixation. Before fixation, cells were pre-extracted on ice in 0.2% Triton X-100 in PBS for 2 min to remove soluble, non-chromatin-bound proteins. Cells were then fixed with 4% (wt/vol) paraformaldehyde for 10 min, permeabilized with 0.2% Triton X-100 in PBS for 10 min, blocked with blocking buffer (4% (wt/vol) BSA, 0.15% Tween 20 and 0.15% Triton X-100 in PBS) for 1 h at room temperature and incubated with the indicated antibodies for 1 h at 37°C. Excess antibody was washed three times with wash buffer (0.15% Tween 20 and 0.15% Triton X-100 in PBS × 1), and cells were stained with Alexa Fluor 488 (1:500; Molecular Probes) secondary antibodies for 1 h at room temperature in the dark, and then washed as above. DNA was stained with DAPI. For quantitative analysis of signal intensity, 12 fields from each well were acquired using a 40× objective of the high-throughput laser-scanning microscope INCell Analyzer 2000 (GE Healthcare). Data analysis was performed using the INCell Analyzer workstation 3.7. For quantifying γH2AX, average nuclear signal intensity per cell measured. The data were exported and plotted as min to max box plot in Graphpad Prism software version 8.

### PARP-trapping

Cells were seeded into 48-well plates in triplicates and grown for 24 h to reach density of ∼80%. Cells were treated either with DMSO, 10 μM PARPi, or 10 μM PARPi and 40 μM VP16 for 4 h as indicated, pre-extracted on ice in 0.2% Triton X-100 in PBS for 2 min to remove soluble, non-chromatin-bound proteins, fixed with 4% (wt/vol) paraformaldehyde for 10 min, permeabilized with 0.2% Triton X-100 in PBS for 10 min, blocked with blocking buffer (4% (wt/vol) BSA, 0.15% Tween 20, and 0.15% Triton X-100 in PBS) for 1 h at room temperature and incubated with PARP1 antibody (Genetex) 1 h at 37°C. Excess antibody was washed three times with wash buffer (0.15% Tween 20 and 0.15% Triton X-100 in PBS × 1), and cells were stained with Alexa Fluor 488 secondary antibody for 1 h at room temperature in the dark, and then washed as above. DNA was stained with DAPI. For quantitative analysis of PARP1 signal intensity, 12 fields were acquired using a 40× objective of the high-throughput laser-scanning microscope INCell Analyzer 2000 (GE Healthcare). Data analysis of PARP1 nuclear intensity was performed using the INCell Analyzer workstation 3.7. Data were exported and plotted in Graphpad Prism software version 8.

### MDA-MB-231 subcutaneous xenograft model

2.5 × 10^6^ MDA-MB-231 cells expressing either scramble or RBM6 shRNA were resuspended in 100 μl PBS and mixed 1:1 with Matrigel (High Concentration – Corning) and injected subcutaneously into the right flank of 5 weeks-old female NOD SCID mice (*n* = 16 mice for each cell line). Tumors were measured using digital calipers. Tumors’ volumes were calculated using the formula: 0.5 × length × width^2^. When tumors reached ∼100 mm^3^, mice were assigned randomly to either control (vehicle: PBS × 1) or cisplatin (5 mg/kg in PBS × 1) treatment (*n* = 8 for each group). Treatment was administered once a week via intraperitoneal injection. Mice were euthanized when the tumors of control mice reached ∼1500 mm^3^ and tumors were excised, weighed and photographed.

### Immunohistochemistry

Tissues were fixed in 4% formalin, then 70% ethanol and processed. Paraffin embedded tissue sections were deparaffinized and rehydrated. Antigen retrieval was performed in 25 mM sodium citrate buffer pH 6.0 using pressurized chamber for 2.5 min. Endogenous peroxidase was blocked with 3% H_2_O_2_ for 15 min. The sections were then incubated with blocking solution (CAS Block) for 30 min to reduce non-specific binding followed by incubation with either RBM6 antibody (abcam) or Ki67 antibody (abcam) overnight. Slides were subsequently incubated with horseradish peroxidase-conjugated anti-rabbit immunoglobulin antibody for 30 min. The enzymatic reaction was detected in a freshly prepared 3,3-diamminobenzidine using DAB peroxidase kit (Vector laboratories) for several min at room temperature. The sections were then counterstained with hematoxylin.

### Immunofluorescence on paraffin-embedded tissue sections

Tissues were fixed in 4% formalin, then 70% ethanol and processed. Paraffin-embedded tissue sections were deparaffinized and rehydrated. Antigen retrieval was performed in 25 mM sodium citrate buffer pH 6.0 using pressurized chamber for 2.5 min. The sections were then incubated with blocking buffer (5% gaot serum + 0.5% BSA in PBT) for 1 h to reduce non-specific binding followed by incubation with the γH2AX antibody (Cell Signaling Technology 9718) or c.caspase 3 antibody (Cell Signaling Technology 9661) overnight. Slides were subsequently incubated with secondary anti-Rabbit Alexa flour 647 (abcam) for 1 h, and mounted by Dako's Fluorescence Mounting Medium.

### RNA-sequencing

Two biological replicates of RNA samples were purified from control and RBM6 knockout MCF10A-H-RAS cells before and after 12 h exposure to IR (5 Gy). RNA sequencing libraries were prepared using TruSeq mRNA library preparation kit. Sequencing was performed on a HiSeq 2500 sequencer with V4 sequencing chemistry to obtain 150 bp paired-end reads. Read depth was 45–50 million reads per sample. Reads were aligned to the hg38 genome with an average unique mapping rate of 75%. Differential expression analysis was performed using DeSeq2 (R platform) (60). Alternative splicing analysis was performed using rMATS (61). Differentially expressed genes were subsequently analyzed for gene set enrichment of biological themes using DAVID bioinformatics platform (62).

### Statistical analysis

Statistical analyses were performed using GraphPad Prism 8 software. Statistical parameters are expressed as the mean ± SD and corresponding sample size and *P*-values are reported in the figures and figure legends. Statistical analysis between two groups were done by paired or unpaired and two-tailed *t*-test.

## RESULTS

### RBM6 depletion leads to elevated levels of γH2AX and confers sensitivity to ionizing radiation (IR)

**Figure 1.**
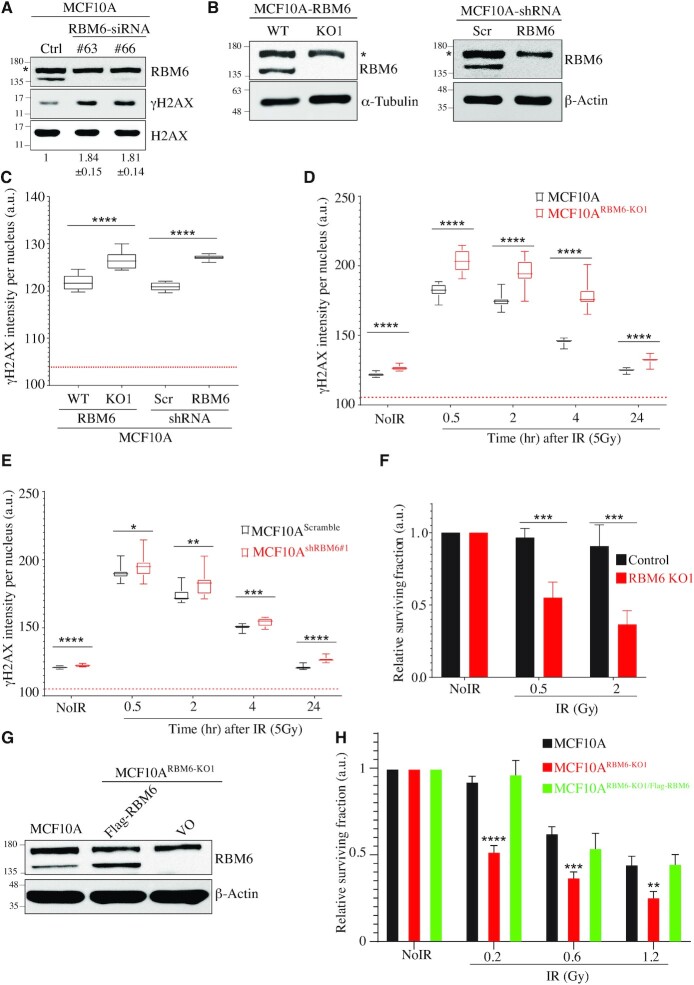
RBM6 depletion leads to elevated levels of γH2AX and confers sensitivity to ionizing radiation. (**A**) Western blot shows γH2AX levels in MCF10A cells transfected either with control or RBM6 siRNAs. H2AX was used as a loading control. Band intensities of γH2AX were normalized to the intensities of their respective H2AX bands and the mean ratio ± SD (*n* = 3) is shown at the bottom of the blot. Two-tailed *t*-test: *P*-value(si#63) = 0.0004; *P*-value(si#66) = 0.0007. (**B**) Left: western blot for RBM6 to validate the generation of RBM6 knockout in MCF10A cells. α-tubulin was used as a loading control. Right: western blot analysis shows protein levels of RBM6 in MCF10A cells expressing either scramble shRNA or shRNA against RBM6. β-actin was used as a loading control. (**C**) RBM6-deficient cells exhibit elevated levels of γH2AX regardless of DNA damage. Control and MCF10A^RBM6-KO1^ and MCF10A^shRBM6#1^ cells were fixed and stained for γH2AX. High-content screening microscope (IN Cell Analyzer 2000; GE Healthcare) was used for automatic image acquisition. Data are presented as min to max box plot (*n* = ∼5000 cells per condition; unpaired *t* test across three replicates: *P< 0.01, **P < 0.001, ***P < 0.0001, ^****^P < 0.00001). Box plots represent γH2AX intensity per nucleus and red dotted line represents background fluorescent signal. (**D, E**) MCF10A^WT^ and MCF10A^RBM6-KO1^ (D), and MCF10A^scramble^ and MCF10A^shRBM6#1^ (E) were exposed to IR (5 Gy) and fixed at the indicated timepoints after IR treatment. NoIR, no treatment. Image acquisition and analysis was performed as described in (C). (**F**) IR sensitizes RBM6-MCF10A deficient cells. MCF10A^WT^ and MCF10A ^RBM6-KO1^ were subjected to short-term growth delay assay. Cells were treated with increasing doses of irradiation and incubated for 48 h. Cell viability was determined using CellTiter 96® proliferation assay and normalized to untreated cells. Data are presented as mean ± SD (*n* = 3, two-way ANOVA; *P< 0.01, **P < 0.001, ***P < 0.0001, ^****^P < 0.00001). (**G**) Western blot analysis for RBM6 protein levels in MCF10A^WT^ and MCF10A^RBM6-KO1^ cells ectopically expressing either RBM6 or an empty vector. β-Actin was used as a loading control. (**H**) Control and RBM6-KO1 cells expressing either RBM6 or empty vector were exposed to increasing dosage of IR and subjected to colony formation assay. Fourteen days post IR exposure, colonies were stained using crystal violet and counted. The number of colonies of IR-exposed cells was normalized to untreated controls. Data presented as mean ± SD (*n* = 3, two-way ANOVA; *P< 0.01, **P < 0.001, ***P < 0.0001, ^****^P < 0.00001). All experiments were performed in triplicates. * indicates unspecific band. The positions of molecular weight markers are indicated to the left of all western blots.

Since many tumor suppressor genes play a role in DNA repair, and since RBM6 is phosphorylated in response to DNA damage including IR (63–65), we were prompted to investigate whether RBM6 is involved in DSB repair. MCF10A-H-Ras cell line, a non-tumorigenic human breast epithelial cell line transformed with H-Ras oncogene (hereafter called MCF10A), was depleted of RBM6 using two different siRNA sequences followed by western blot for γH2AX, a surrogate marker for DSBs. Results show that RBM6-depleted cells exhibit ∼1.8-fold increase of γH2AX when compared to control cells (Figure 1A). Similar results were observed in the triple-negative breast cancer cell line MDA-MB-231 (hereafter called MDA231) and in HeLa cells (Supplementary Figure S1A, B). To further substantiate this phenotype, we measured γH2AX levels in control and RBM6 knockout MCF10A cell line (hereafter called MCF10A^RBM6-KO1^). To establish MCF10A^RBM6-KO1^ cells, we used a pair of specific guide-RNAs guiding Cas9-D10A nickase to RBM6 exon 3 (66). RBM6 knockout clones were confirmed by DNA sequencing (Supplementary Figure S2) and western blot (Figure 1B). Results show that γH2AX levels in MCF10A^RBM6-KO1^ are significantly higher than in control cells (Figure 1C and Supplementary Figure S3A). Similar results were also observed in MCF10A cells depleted of RBM6 using shRNA (hereafter called MCF10A^shRBM6#1^) (Figure 1B, C). To address whether γH2AX levels in RBM6-deficient cells are also elevated after exogenous DNA damage, MCF10A^RBM6-KO1^ and MCF10A^shRBM6#1^ cells were exposed to IR, followed by γH2AX quantification at the indicated timepoints after IR exposure. We found that IR resulted in increased levels of γH2AX in RBM6-deficient cells compared to control cells (Figure 1D, E and Supplementary Figure S3B). These observations suggest that RBM6 might be required for intact repair of endogenous and IR-induced DSBs. Corollary to this, RBM6 knockout cells are markedly more sensitive to increasing doses of IR when compared to control cells (Figure 1F). In addition, transducing MCF10A^RBM6-KO1^ cells with a lentiviral vector expressing RBM6 reverses the sensitivity to IR (Figure 1G, H). Altogether, the hypersensitivity of RBM6-depleted cells to IR suggests that RBM6 is required for intact repair of IR-induced DNA damage.

### RBM6 fosters HR of DSBs

**Figure 2.**
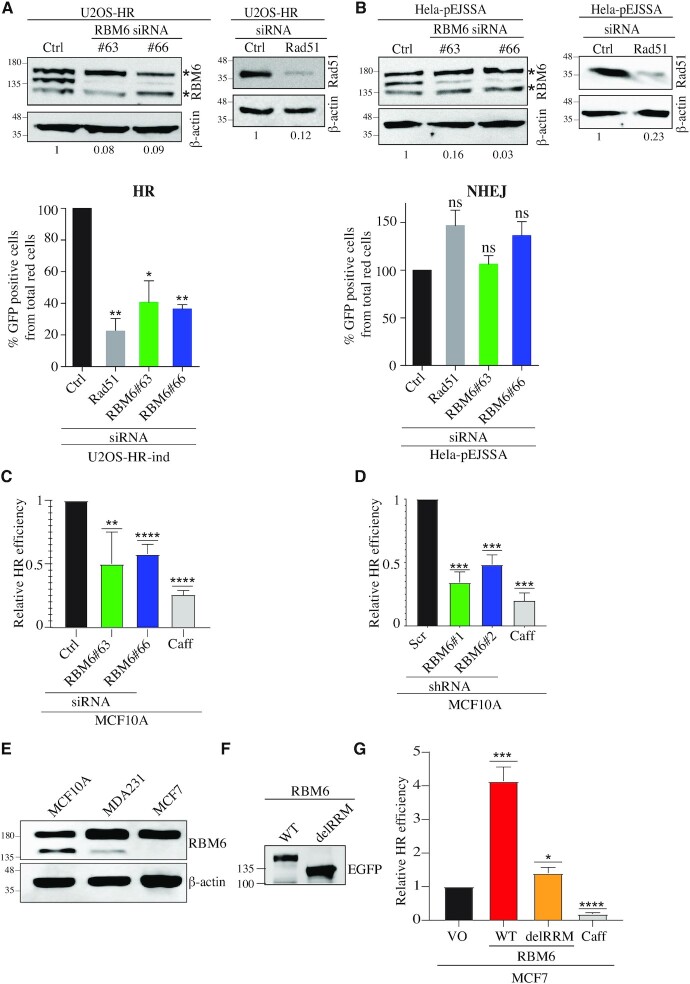
RBM6 promotes homologous recombination (HR) of double-strand breaks. (**A**) RBM6 depletion impairs HR of DSBs induced by I-Sce-I endonuclease. *Top*: U2OS-HR cells were transfected with siRNAs against RBM6 and Rad51 genes or control siRNA and subjected to western blot analysis with the indicated antibodies. β-Actin was used as a loading control. Band intensities of RBM6 and Rad51 were normalized to the intensities of their respective β-actin bands and the normalized ratio is shown at the bottom of the blot. *Bottom*: U2OS-HR cells were transfected with RBM6 or Rad51-specific siRNAs, and the percentage of GFP-positive cells was analyzed by fluorescence-activated cell sorting 72 h after transfection. Rad51 serves as a positive control. Data are presented as mean ± SD of three independent experiments. (**B**) RBM6 depletion had no detectable effect on the integrity of NHEJ. *Top:* HeLa-pEJSSA cells were transfected with siRNAs against RBM6 and Rad51 genes or control siRNA and subjected to western blot with the indicated antibodies. β-Actin was used as a loading control. Band intensities of RBM6 and Rad51 were normalized to the intensities of their respective β-actin bands and the normalized ratio is shown at the bottom of the blot. *Bottom*: RBM6‐depleted HeLa cells containing pEJSSA plasmid stably integrated into their genome were used to determine the efficiency of NHEJ. The percentage of GFP-positive cells was analyzed by fluorescence-activated cell sorting 72 h after transfection. Data are presented as mean ± SD of three independent experiments. (**C**) RBM6 depletion impairs HR of endogenous DSBs at the LMNA gene. MCF10A cells were transfected with the indicated siRNAs, 24 h after siRNA transfection, cells were transfected with plasmids expressing Cas9 endonuclease, LMNA-donor plasmid or Red-Monomer (MR) tag. 72 h after transfection, cells were harvested, and the percentage of GFP-positive cells from the total number of red cells was determined by flow cytometry. Data are presented as mean ± SD of three independent experiments. (**D**) as in (C), except that MCF10A cells were stably infected with lentiviral vectors expressing either scramble or RBM6 shRNAs. (**E**) Western blot shows the levels of RBM6 in MCF10A, MDA231 or MCF7 cells. β-actin used as a loading marker. (**F**) MCF7 cells were transfected with RBM6^WT^ or RBM6^delRRM^ and subjected to western blot analysis. (**G**) MCF7 cells were complemented with empty vector or vectors expressing either RBM6^WT^ or RBM6^delRRM^ fused to Mono-Red (MR). HR efficiency was measured as described in (C). Caffeine was used as a positive control. Data are presented as mean ± SD of three independent experiments. *P< 0.01, **P < 0.001, ***P < 0.0001, ^****^P < 0.00001. * indicates unspecific bands. The positions of molecular weight markers are indicated to the left of all western blots.

Since IR predominantly induces DSBs and RBM6-deficient cells are hypersensitive to IR, we sought to determine the effect of RBM6 on DSB repair. Toward this end, U2OS-HR-ind and HeLa-pEJSSA cells were used to determine the integrity of HR and NHEJ of I-SceI-induced DSBs, respectively (13) (Supplementary Figure S4A, B). RBM6 knockdown in U2OS-HR-ind cells leads to a decrease of 60–65% in GFP-positive cells (represents cells that repair DSBs by HR) as compared to control siRNA-treated cells. RAD51 was used as a positive control and its depletion results in ∼80% decrease in the GFP-positive cells (Figure 2A). On the other hand, RBM6 depletion in HeLa-pEJSSA cells has no prominent effect on NHEJ (Figure 2B). To further confirm the effect of RBM6 on HR, we used a state-of-the-art fluorescent mClover-based reporter assay, where we measured HR efficiency of Cas9-induced DSB at the LMNA gene in MCF10A cells (58). RBM6-proficient and -deficient cells were co-transfected with three plasmids: The first expresses Cas9 and gRNA against LMNA exon 1. The second contains mClover-LMNA homology donor sequence, and the third expresses DsRed reporter that is used as a transfection control. HR repair of DSB at the LMNA gene will generate mClover-LMNA fusion. Therefore, HR efficiency is determined as the percentage of DsRed-positive cells that express mClover (Supplementary Figure S4C). Results show that RBM6 depletion in MCF10A cells, using either siRNA or shRNA leads to a reduction in HR efficiency (Figure 2C-D). Similar results were obtained in RBM6-deficient MDA231 cells (Supplementary Figure S5). Next, we sought to address whether ectopic expression of RBM6 affects HR efficacy. Toward this end, we took advantage of MCF7 breast cancer cell line, which is known to lack RBM6 expression (43) (Figure 2E). We observed that expression of RBM6 in MCF7 increased HR efficiency of Cas9-induced DSB at the LMNA gene (Figure 2F, G). Interestingly, expression of an RBM6 mutant that lacks the RRM domain (Hereafter RBM6^delRRM^) has negligible effect on the integrity of HR, suggesting that RBM6 regulates HR in an RRM-dependent manner (Figure 2G). Lastly, we sought to confirm that the increase in mClover fluorescence following RBM6 overexpression reflects the increase in HR efficiency and not due to prospective effect of RBM6 on the expression or the stability of the fluorescent mClover gene. Toward this end, we determined HR efficiency using mClover-based reporter assay in mock and caffeine treated MCF7 cell overexpressing RBM6. Our results show that caffeine treatment reduces HR efficiency in MCF7 cells overexpressing RBM6, arguing against the possibility that the increase in mClover fluorescence is due to an unanticipated effect of RBM6 on the stability of mClover fluorescent protein (Supplementary Figure S6). Altogether, our results provide firm evidence that RBM6 is required for intact HR repair, but not NHEJ, of DSBs in a cell line-independent manner.

### Transcriptome analysis of RBM6 knockout cells before and after DNA damage

**Figure 3.**
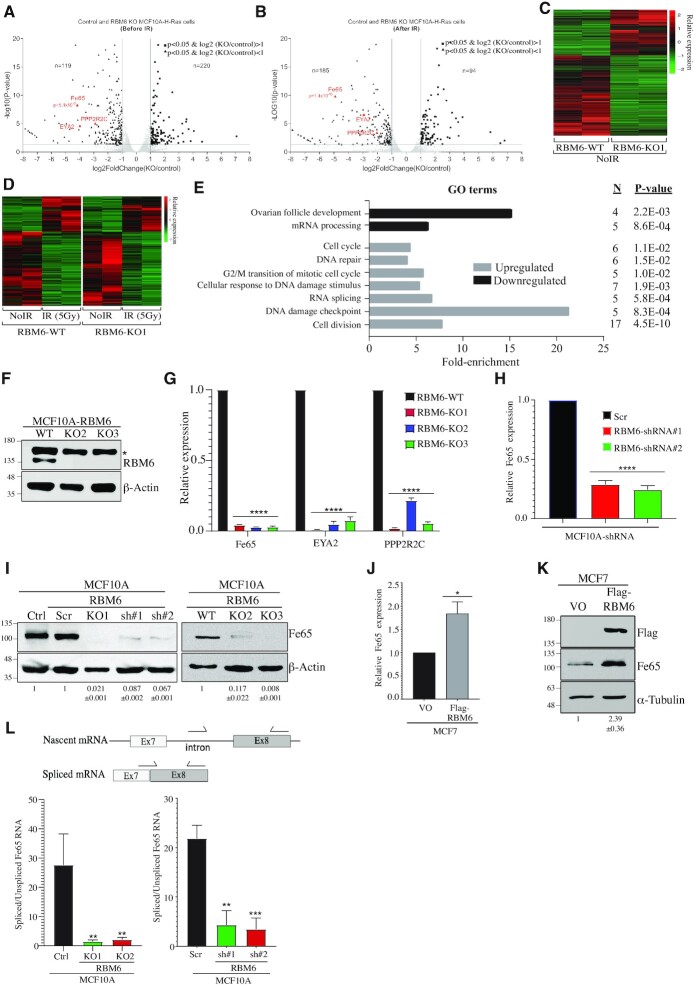
Transcriptome analysis identifies Fe65 as a target gene of RBM6. (**A, B**) Volcano plots showing the differentially expressed genes between control and RBM6-knockout (KO1) MCF10A cells before (A) and after 12hr exposure to IR (5Gy) (B). DDR genes downregulated in RBM6 KO1 cells are highlighted in red. (**C**) Heatmap summarizing the differentially expressed genes in RBM6-knockout cells compared to control cells without IR treatment. The relative expression of genes with |log_2_(KO/control) > 1| and *P*< 0.05 is presented in each row for control and RBM6-knockout cells. (**D**) Heatmap summarizing IR-induced differentially expressed genes compared to untreated cells in control (left) and RBM6 knockout (right) MCF10A cells. Each row represents the relative expression of genes with |log_2_(+IR/–IR) > 1| and *P*< 0.05 in both control (left) and RBM6-knockout (right) cells. (**E**) Gene ontology analysis of genes from (D) that show differential response to IR in RBM6-knockout cells compared to control cells (**F**) Western blot analysis validating RBM6 knockout in two additional MCF10A clones. β-Actin was used as a loading marker. * indicates unspecific band. (**G**) RT-qPCR analysis showing the relative expression of Fe65, EYA2, and PPP2R2C in three different MCF10A RBM6 knockout clones compared to control MCF10A cells. Gene expression was normalized to the levels of GAPDH transcript. (**H**) RT-qPCR analysis showing the relative expression of Fe65 in MCF10A cells depleted of RBM6 using two different shRNA sequences. Gene expression was normalized to the levels of GAPDH transcript. (**I)** Western blot analysis using Fe65 antibody shows that RBM6 knockout or knockdown led to a severe reduction in Fe65 protein level. β-Actin was used as a loading marker. Band intensities of Fe65 from three independent experiments were normalized to the intensities of their respective β-actin bands and the mean normalized ratio ± SD is shown at the bottom of the blot. Two-tailed *t*-test: *P*-value < 0.0001. (**J**) RT-qPCR analysis showing the relative expression of Fe65 in MCF7 cells complemented with Flag-RBM6. Gene expression was normalized to the levels of GAPDH transcript. (**K**) Western blot analysis shows that complementing MCF7 cells with Flag-RBM6 led to an increase in Fe65 protein level. α-Tubulin was used as a loading marker. Band intensities of Fe65 from three independent experiments were normalized to the intensities of their respective α-Tubulin bands and the mean normalized ratio ± SD is shown at the bottom of the blot. Two-tailed *t*-test: *P*-value = 0.003. (**L**) Schematic diagram showing the location of qPCR primers (arrows) used to detect either nascent or spliced Fe65 transcript (Top). RT-qPCR analysis using primers to detect nascent (unspliced) and spliced Fe65 transcript. Shown is the ratio between the relative expression of spliced and unspliced Fe65 transcript in MCF10A RBM6-KO1 cells (left) or MCF10A cells depleted of RBM6 using two different shRNA sequences (right). Gene expression was normalized to the levels of GAPDH transcript. *P< 0.01, **P < 0.001, ***P < 0.0001, ^****^P < 0.00001. All RT-qPCR Data are presented as mean ± SD of three independent experiments.

To test whether RBM6 promotes HR via its canonical function by regulating gene expression and alternative splicing (AS) of key HR genes, RNA samples were purified from control and MCF10A^RBM6-KO1^ cells before and 12 h after exposure to IR (5 Gy) and analyzed by RNA-sequencing (RNA-seq). In the absence of DNA damage, 339 transcripts were differentially expressed in MCF10A^RBM6-KO1^ cells compared to control cells (|fold-change (FC)| ≥  2; *P*_adj_ < 0.05) (Supplementary Table S1, Supplementary Figure S7 and Figure 3A–C). Gene set enrichment analysis revealed several pathways that were altered following RBM6 depletion (Supplementary Figure S8) including HR and cancer-related pathways.

As expected, IR-induced DNA damage led to significant changes in gene expression profile both in control MCF10A and MCF10A^RBM6-KO1^ cells (Figure 3D). To identify IR-induced alterations in gene expression regulated by RBM6 activity, we compared IR-induced differential expression profile of control and MCF10A^RBM6-KO1^ cells. Interestingly, most IR-induced changes in gene expression are observed both in control and MCF10A^RBM6-KO1^ cells. However, the small subset of genes with a different IR response between control and MCF10A^RBM6-KO1^ cells are linked to DNA damage checkpoint, cell cycle regulation and DNA repair pathways, substantiating the emerging function of RBM6 in DNA repair (Figure 3E).

In this latter regard, we identified three DDR genes (Fe65 (APBB1) (67,68); EYA2 (69) and PPP2R2C (70)) whose expression was significantly downregulated (∼4–8-fold) in an RBM6-depenedent manner but regardless of IR (Figure 3A, B). To quantitatively validate the RNA-seq data, we conducted real-time qRT-PCR evaluation of the aforementioned DDR genes in control and MCF10A^RBM6-KO1^ cells. In agreement with the RNA-seq data, we found that the expression levels of Fe65, EYA2 and PPP2R2C are remarkably reduced in MCF10A^RBM6-KO1^ cells when compared to control cells. Similar reduction in the expression level of the 3 DDR genes was also observed in two additional RBM6 knockout MCF10A clones that were established using CRISPR-Cas9 at different regions of RBM6 coding sequence (Figure 3F, G and Supplementary Figure S2). Altogether, these findings confirm that Fe65, EYA2 and PPP2R2C are bona fide RBM6-regulated genes.

### RBM6 regulates alternative splicing coupled to nonstop-decay of the HR factor Fe65

While all RBM6-dependent transcriptome changes are potentially interesting, we focused on Fe65 gene primarily because it promotes HR of DSBs (67,68,71). To further corroborate that RBM6 regulates Fe65, we tested its expression in MCF10A cells depleted of RBM6 using two different shRNA sequences. Results show that Fe65 mRNA levels are dramatically decreased upon RBM6 knockdown (Figure 3H). Next, we tested the protein level of Fe65 in RBM6-deficient MCF10A cells. Western blot analysis shows that RBM6 knockout or knockdown led to a sever reduction in Fe65 protein level (Figure 3I). Appropriately, re-expressing RBM6 in MCF7 cells, which lack RBM6, led to a significant increase in the RNA and protein levels of Fe65 (Figure 3J, K). Similarly, re-expression of RBM6 in MCF10A^RBM6-KO1^ cells increases Fe65 protein levels (Supplementary Figure S9). Collectively, our results identified Fe65 as a novel RBM6 regulated gene. In support of this, RNA-seq expression data of 1375 cancer cell lines from diverse origins show a mild, but statistically significant, correlation between RBM6 and Fe65 expression (Supplementary Figure S10A) (72). Remarkably, similar correlation between RBM6 and Fe65 expression was also observed in 10,953 cancer patients (Supplementary Figure S10B) including breast cancer patients (Supplementary Figure S10C) (49).

Next, we sought to determine how RBM6 regulates Fe65 expression. Toward this end, we measured the levels of the nascent unspliced Fe65 transcript using primers encompassing exon-intron junction and the spliced mRNA using one of the primers designed to overlap an exon-exon junction in control and MCF10A^RBM6-KO1^ cells (Figure 3L). While RBM6 knockout cells show 2–3-fold reduction in the levels of the nascent Fe65 transcript, a striking reduction of 50-fold was observed in the levels of the spliced Fe65 mRNA (Figure 3L and Supplementary Figure S11). Similar results were also obtained following shRNA depletion of RBM6 (Figure 3L). Altogether, we concluded that RBM6 predominantly regulates the levels of the spliced Fe65 mRNA and has a mild effect on Fe65 transcription rate. This result supports the notion that RBM6 fosters Fe65 splicing and or mRNA stability. Since Fe65 undergoes alternative splicing (AS), we sought to determine whether RBM6 regulates AS of Fe65. To identify AS events regulated by RBM6, we used the rMATS-based pipeline (61). Notably, rMATS analysis predicted ∼2000 alternative splicing events regulated by RBM6, most commonly affecting cassette exons (Supplementary Table S2). Our observations are in line with a previously described role of RBM6 in modulating alternative splicing (43). However, due to the low abundance of Fe65 transcript in MCF10A^RBM6-KO1^ cells, the sequencing depth was insufficient to observe splicing alterations in Fe65 (Supplementary Table S1).

Interestingly, while looking at the Ensembl database, we found that Fe65 has a splice variant annotated as a nonstop-decay (NSD) transcript (Transcript ID ENST00000608435.5). NSD is a surveillance mechanism that detects mRNA transcripts that lack a stop codon and targets them for rapid degradation. Fe65 NSD variant is produced following the skipping of the canonical 3′ acceptor splice site and the usage of alternative 3′ splice site (A3′SS) downstream the stop codon in exon 15. To verify the presence of Fe65 NSD variant, RNA was extracted from MCF10A cells and subjected to RT-PCR using specific primers that amplify the NSD variant. DNA sequencing confirms the presence of Fe65 NSD variant in MCF10A cells (Supplementary Figure S12).

**Figure 4.**
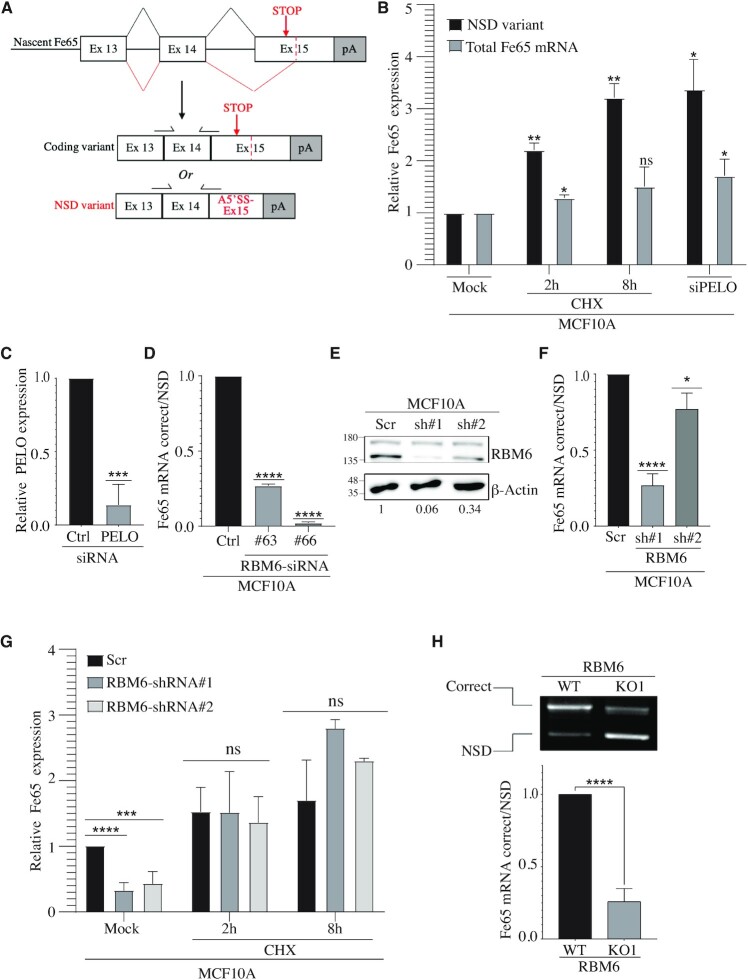
Fe65 harbors an alternatively spliced variant targeted for nonstop-decay (NSD). (**A**) Schematic illustration of the alternative splicing event that results in the correct Fe65 coding variant or the NSD variant. (**B**) Cyclohexamide (CHX) treatment increases the levels of Fe65 NSD variant. MCF10A cells were left untreated or treated with 30 ug/ml CHX for 2 or 8 h. RNA was extracted from the cells and total Fe65 mRNA levels (spliced Fe65 mRNA detected by primers from Figure 3L) or NSD variant (specific primers detecting Fe65 NSD mRNA variant as shown in (A)) were measured by real-time PCR. PELO gene was depleted by siRNA and used as positive control. Results shown are typical of three independent experiments. (**C**) Real time PCR analysis showing the relative reduction in PELO mRNA levels following siRNA transfection. (**D**) RBM6 depletion increases the production of Fe65 NSD variant compared to the correct variant. MCF10A cells were transfected with two siRNAs against RBM6. Cells were harvested and RNA was extracted followed by real-time RT-qPCR analysis. Graph shows the relative expression of the correct variant/NSD variant. Data are presented as mean ± SD of three independent experiments. (**E**) Western blot analysis shows protein levels of RBM6 in MCF10A cells expressing either scramble shRNA or shRNAs against RBM6. β-Actin was used as a loading control. Band intensities of RBM6 were normalized to the intensities of their respective β-actin bands and the normalized ratio is shown at the bottom of the blot. (**F**) As in (D) except of using MCF10A cells stably infected with lentiviral vectors expressing either scramble or RBM6 shRNAs. (**G**) CHX treatment in RBM6-depleted cells restored the levels of Fe65 to similar levels as the control cells. MCF10A cells stably infected with lentiviral vectors expressing either scramble or RBM6 shRNAs were left untreated or treated with 30ug/ml cycloheximide (CHX) for 2 or 8 h. RNA was extracted and real-time RT-qPCR analysis was performed using primers to detect total levels of Fe65. Data presented are mean of three independent experiments ± SD. (**H**) Control and RBM6-KO1 MCF10A cells were treated with 30ug/ml CHX for 8 h to stabilize the Fe65 NSD transcript. RNA was extracted and subjected to reverse transcription followed by co-amplification of the correct and NSD transcripts using the same primer pair. PCR products were then analyzed by gel electrophoresis (top). Band intensities of the correct and NSD amplified products from three independent experiments were quantified and the normalized ratio is presented (bottom). *P< 0.01, **P < 0.001, ***P < 0.0001, ^****^P < 0.00001. The positions of molecular weight markers are indicated to the left of all western blots.

Since an initial round of mRNA translation is needed for degrading the NSD mRNA variant, several translation elongation inhibitors, including cycloheximide (CHX), have been reported to block the NSD pathway. Therefore, to confirm that the decay of Fe65 transcript is regulated by the NSD pathway, we measured the levels of Fe65 NSD mRNA variant before and after CHX treatment. Results show that CHX treatment leads to a remarkable increase in Fe65 NSD variant levels (Figure 4A-B). It was reported that PELO (Pelota mRNA Surveillance and Ribosome Rescue Factor) protein plays an important role in NSD as it recognizes stalled ribosomes and hence promotes the degradation of NSD mRNA molecules (73–78). In agreement with this, we observed that PELO knockdown leads to a significant increase in Fe65 NSD variant (Figure 4B, C). Together, our results provide strong evidence that Fe65 transcript is regulated by the NSD pathway. To determine whether RBM6 regulates alternative splicing-coupled NSD (AS-NSD) of Fe65, we measured the level of the correct and the NSD variant of Fe65 in RBM6-deficent MCF10A (Figure 4A). Our results revealed that the production of Fe65 NSD variant is significantly elevated following RBM6 depletion (Figure 4D-F and Supplementary Figure S13). These results suggest that RBM6 regulates the transcript level of Fe65, at least in part, through the NSD pathway. In agreement with this, blocking the NSD pathway by CHX treatment restored the levels of Fe65 mRNA in RBM6-deficient cells (Figure 4G). To further substantiate the switch toward the Fe65 NSD variant upon RBM6 depletion, we co-amplified the correct and NSD transcripts using the same primer pair after CHX treatment. Gel electrophoresis of the amplified PCR products confirms the switch towards Fe65 NSD variant in RBM6 knockout cells (Figure 4H). Taken together, these results confirm that RBM6 counteracts the production of Fe65 NSD variant.

**Figure 5.**
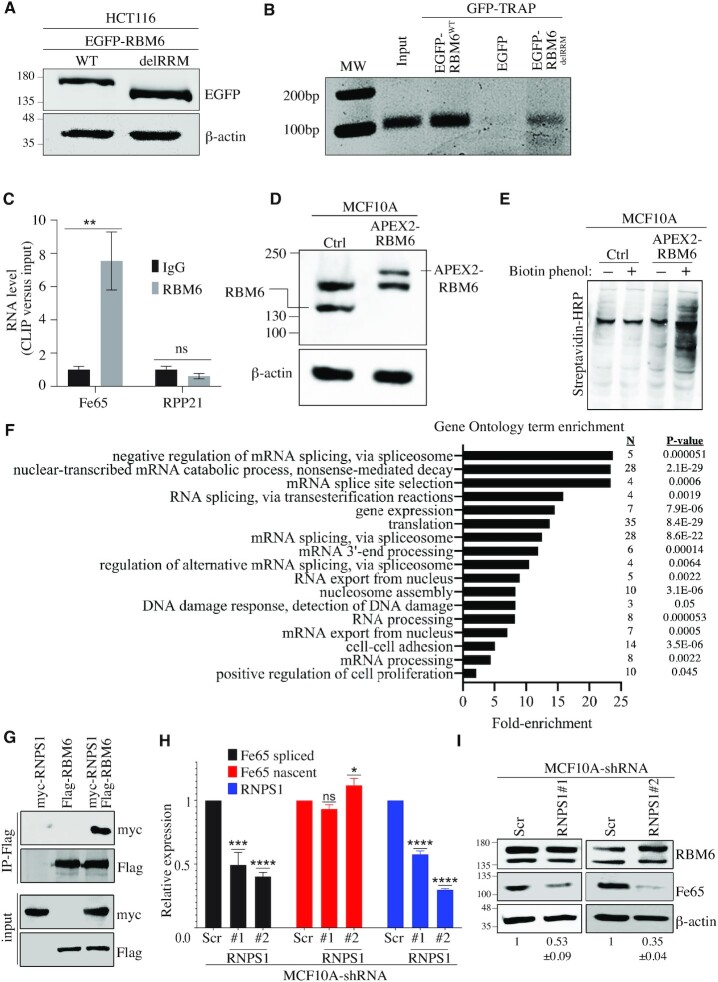
RBM6 regulates alternative splicing-coupled nonstop-decay (AS-NSD) of Fe65. (**A**) Western blot showing the expression of EGFP fused to RBM6 (EGFP-RBM6^WT^) or EGFP fused to RBM6 mutant lacking RRM domains (EGFP-RBM6^delRRM^) 48 h post-transfection in HCT116 cells. β-Actin was used as a loading control. (**B**) RNA immunoprecipitation (RIP) using GFP-TRAP followed by RT-PCR for Fe65 transcript. Cells were transfected with vectors encoding EGFP only, EGFP-RBM6^WT^ or EGFP-RBM6^delRRM^. Forty-eight hours post-transfection, cells were harvested and subjected to GFP-TRAP assay followed by RNA extraction and RT-PCR analysis using primers specific to Fe65 RNA. Results are representative of three independent experiments. (**C**) CLIP-qPCR showing binding of RBM6 to Fe65 RNA. MCF10A cells were UV-crosslinked and immunoprecipitated using IgG or RBM6 antibody followed by RNA extraction and qRT-PCR analysis using primers detecting either Fe65 or RPP21 RNA. Relative RNA enrichment over input from three experiments is presented. (**D**) Western blot analysis validating the establishment of MCF10A cell line expressing APEX2-RBM6 fusion. β-Actin used as a loading marker. (**E**) Western blot analysis confirming APEX2-RBM6 functionality in MCF10A cells. Control and APEX2-RBM6 expressing cells, incubated with 0.5 mM Biotin Phenol for 4 h and labelling reaction was activated using 1 mM H2O2 for 2 min. Labelling reaction was quenched and cells were subjected to western blot analysis using streptavidin–HRP to detect biotinylated proteins. (**F**) Gene ontology analysis of mass spectrometry data showing significantly enriched terms in APEX2-RBM6 expressing cells compared to control MCF10A cells. (**G**) HCT116 cells were transfected with plasmids expressing either Flag-RBM6, Myc-RNPSI or co-transfected with both plasmids. Then, cells were subjected to immunoprecipitation using Flag antibody and immunoblotted using the indicated antibodies. (**H**) RT-qPCR analysis showing the relative expression of nascent Fe65 RNA, spliced Fe65 mRNA, and RNPS1 in MCF10A cells expressing either scramble or shRNAs against RNPS1. Gene expression was normalized to the levels of GAPDH transcript. Data presented are mean of three independent experiments ± SD. (**I**) Western blot analysis shows that RNPS1 depletion led to a significant reduction in Fe65 protein levels in MCF10A cells. β-Actin was used as a loading marker. Band intensities of Fe65 from three independent experiments were normalized to the intensities of their respective β-actin bands and the mean normalized ratio ± SD is shown at the bottom of the blot. Two-tailed *t*-test: *P*-value (shRNPS1 #1) = 0.0057; *P*-value(shRNPS1 #2) = 0.0005. The positions of molecular weight markers are indicated to the left. *P< 0.01, **P < 0.001, ***P < 0.0001, ^****^P < 0.00001.

Next, we sought to determine whether RBM6 binds Fe65 transcript. Toward this end, cells expressing wild-type or RBM6^delRRM^ fused to EGFP were subjected to RNA immunoprecipitation (RIP) using GFP-TRAP assay followed by RT-PCR for Fe65 transcript. RIP data show that RBM6 binds Fe65 transcript in an RRM-dependent manner (Figure 5A, B). The requirement of the RRM domain for binding Fe65 transcript may explain why the expression of RBM6^delRRM^ mutant in MCF7 cells did not significantly increase HR efficiency (Figure 2G). These observations prompted us to look at the endogenous interaction between RBM6 and Fe65 transcript by cross-linking immunoprecipitation (CLIP). Our results show that RBM6 directly binds Fe65 transcript (Figure 5C). In line with this, we noticed that Fe65 has 14 sites containing RBM6 consensus binding motif ‘CUCUGAA’ that was previously identified by CLIP-seq assay (43). Concordantly, RBM6 CLIP assay showed that RBM6 doesn’t bind Rpp21 transcript that lacks the ‘CUCUGAA’ motif (Figure 5C), Collectively, our results show that RBM6 directly binds Fe65 mRNA and identify RBM6 as a novel regulator of AS-NSD of Fe65 gene, presumably in a direct manner.

To further understand how RBM6 regulates alternative splicing-coupled NSD of Fe65, we sought to map RBM6 interactome using ascorbate peroxidase (APEX2)-based proximity labelling combined with mass spectrometry (79). Toward this end, we used CRISPR-cas9 methodology to establish MCF10A cell line expressing APEX2 fused to the N-terminal of RBM6, hereafter called MCF10A^APEX2-RBM6^ (Figure 5D and Supplementary Figure S14). The peroxidase activity of APEX2-RBM6 fusion was confirmed (Figure 5E), and RBM6 proximal proteins that were biotinylated by APEX2 activation using hydrogen peroxidase (H2O2) in the presence of biotin-phenol were isolated and subjected to mass spectrometry. We identified 173 (*P*-value < 0.05) RBM6 proximity-interaction partners. Gene ontology analysis shows that RBM6 is associated with many proteins that are involved in RNA splicing (Figure 5F). Among the most enriched novel proximity interactors of RBM6 is RNPS1 (Supplementary Table S3), a known component of a post-splicing mRNP complex involved in splicing, mRNA export, and nonsense-mediated mRNA decay (80,81). Co-immunoprecipitation assay confirmed RBM6–RNPS1 interaction (Figure 5G). Remarkably, similar to RBM6 knockdown, shRNA depletion of RNPS1 leads to a significant reduction in the levels of the spliced, but not nascent, Fe65 RNA (Figure 5H) and accordingly, results in a significant decrease in Fe65 protein levels (Figure 5I). On the other hand, RNPS1 depletion has no detectable effect on RBM6 protein levels (Figure 5I). Our data identified therefore a previously unrecognized role of RNPS1 in regulating Fe65 levels. Moreover, it raises a possibility that RBM6 and RNPS1 cooperate in regulating alternative splicing-coupled NSD of Fe65.

### RBM6 promotes HR of DSBs by regulating the expression of Fe65 gene

**Figure 6.**
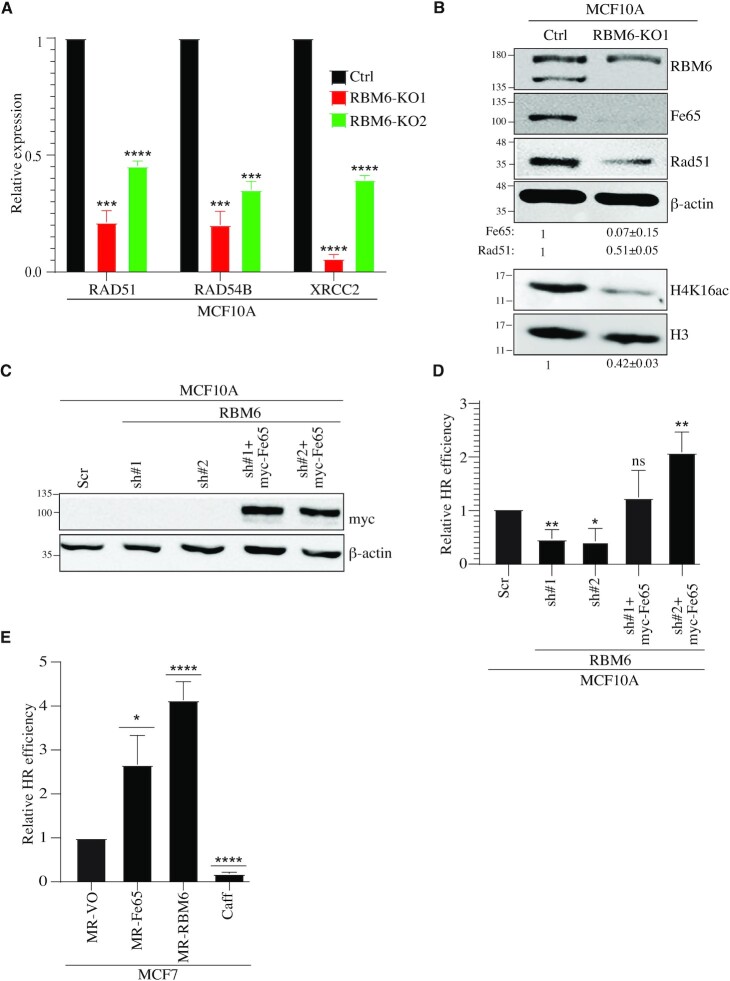
RBM6 promotes HR of DSBs via regulating the expression of Fe65. (**A**) RBM6 depletion reduces the expression of RAD51, RAD54B and XRCC2 genes. RNA was extracted from MCF10A control or RBM6-KO cells and analyzed by real-time RT-qPCR to detect the mRNA levels of the indicated genes. (**B**) Western blot analysis shows that RBM6 depletion reduces Fe65, Rad51 and H4K16ac levels. H3 and β-actin were used as loading controls. Band intensities of Fe65, Rad51 were normalized to the intensities of their respective β-actin bands. H4K16ac bands intensities were normalized to the intensities of their respective H3 bands. The mean normalized ratio ± SD (*n* = 3) is shown at the bottom of the blot. Two-tailed *t*-test: *P*-value(Fe65) = 0.0057; *P*-value(Rad51) = 0.0017; *P*-value(H4K16ac) = 0.0010. (**C**) Western blot shows myc-Fe65 expression in RBM6-deficient MCF10A expressing myc-Fe65. β-Actin was used as a loading control. (**D**) Fe65 expression rescues the deficiency in HR of DSBs seen in RBM6 depleted cells. RBM6 deficient cells were transfected with vector expressing myc- Fe65 and HR efficiency was measured as described in Figure 2C. (**E**) MCF7 cells expressing MR-Fe65 or MR-RBM6 show elevated HR. HR efficiency was measured as in Figure 2C. Caffeine was used as a positive control. Data presented are mean of three independent experiments ± SD. *P< 0.01, **P < 0.001, ***P < 0.0001, ^****^P < 0.00001. The positions of molecular weight markers are indicated to the left of all western blots.

Fe65-null mouse embryonic fibroblasts exhibit elevated levels of γH2AX and are hypersensitive to IR and genotoxic agents, such as etoposide (82). It was also shown that Fe65 contributes to DSB repair by at least two distinct pathways: First, Fe65 interacts with Tip60 acetyltransferase and promotes its recruitment to DSB sites. Accordingly, Fe65 depletion results in a decrease in H4 acetylation which is accompanied by reduction in HR of DSBs (67). Second, ChIP-sequencing analysis revealed that Fe65 is enriched at the promoter regions of genes that are implicated in DDR. Specifically, it was shown that Fe65 promotes the transcription of key genes implicated in HR of DSBs such as, RAD51, RAD54B and XRCC2 (71). Since RBM6 positively regulates Fe65 levels, we sought to determine whether RBM6 deficiency affects the expression of the aforementioned HR genes and histone acetylation levels. Intriguingly, two different RBM6 KO clones show significant reduction in the transcript levels of RAD51, RAD54B and XRCC2 genes, when compared to control MCF10A cells (Figure 6A). Moreover, immunoblot analysis revealed that the levels of H4 acetylation and Rad51 protein are pronouncedly reduced in RBM6-deficient cells (Figure 6B). Intriguingly, exogenous overexpression of either Fe65 or RBM6 in MCF7 cells, that lack RBM6, increases the expression of RAD51, RAD54B (Supplementary Figure S15). Altogether, we concluded that the loss of RBM6 phenocopies Fe65 deficiency. Our results thus far support the hypothesis that RBM6 promotes HR of DSBs by regulating the expression of Fe65 gene. To test this assumption, we expressed myc-Fe65 in RBM6-deficient MCF10A and measured HR efficiency of Cas9-induced DSB at the LMNA gene. Our results showed that Fe65 overexpression rescues the HR defect of RBM6-deficient MCF10A and HeLa cell (Figure 6C, D and Supplementary Figure S16A). Moreover, Fe65 overexpression increases HR efficiency in RBM6-proficient cells (Supplementary Figure S16B). Additionally, Fe65 expression in MCF7 cells that lack RBM6 led to a pronounced increase in HR efficiency (Figure 6E). These findings hence imply epistatic function of RBM6 and Fe65.

### RBM6 deficiency confers sensitivity to ATM and PARP inhibition

**Figure 7.**
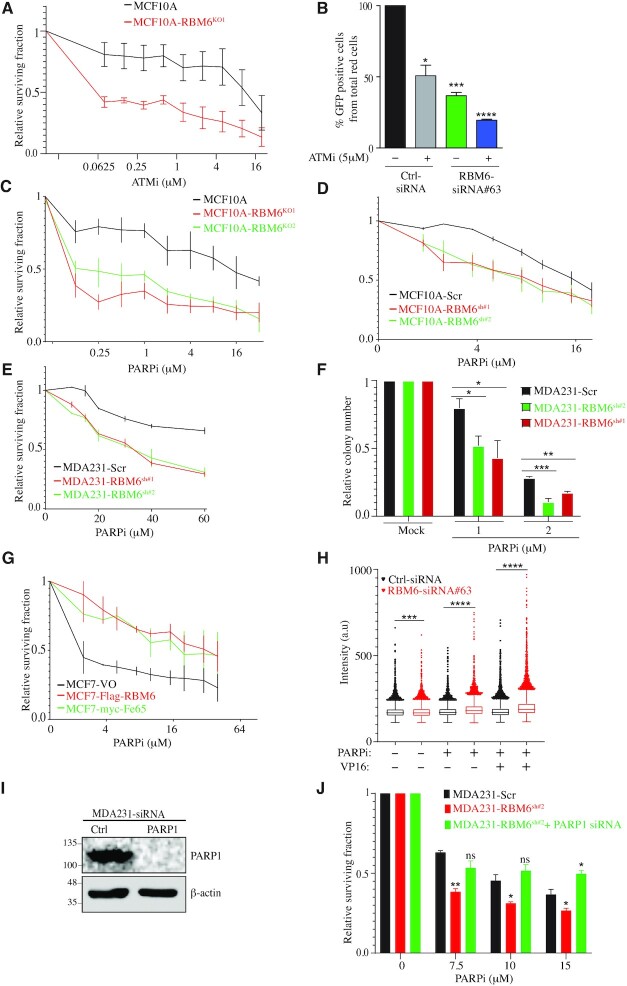
RBM6 depletion sensitizes cells to ATM and PARP inhibition. (**A**) MCF10A RBM6-KO1 cells are hypersensitive to ATM inhibition. Cell viability was determined by the CellTiter 96^®^ proliferation assay. (**B**) ATM inhibition exacerbate the HR deficiency in RBM6 depleted cells. U2OS-HR cells were transfected with siRNA against RBM6 and left untreated or treated with 5μM ATMi and tested for HR efficiency as described in Figure 2A. (**C, D**) PARP inhibition sensitizes MCF10A RBM6-KO1 and KO2 (C) or RBM6-shRNA#1 and #2 (D) cells. Cell viability was assessed by the CellTiter 96^®^ proliferation assay. (**E**) As in (D), except that MDA231 cells were used. (**F**) MDA231 cells constitutively depleted of RBM6 were treated with PARP inhibitor. Cell viability was measured by clonogenic assay. (**G**) MCF7 cells expressing Flag-RBM6 or myc-Fe65 were treated with PARP inhibitor and cell viability was measured by CellTiter 96^®^ proliferation assay. Data presented are mean of three independent experiments ± SD. (**H**) Image-based quantification of PARPi-induced PARP1 trapping. MDA231 cells were treated with 10 μM olaparib and 40 μM VP16 for 4 h as indicated, pre-extracted on ice in 0.2% Triton X-100 for 2 min to remove soluble, non-chromatin-bound proteins, and stained for PARP1 and DNA content. Experiment was performed in triplicates. (**I**) Western blot analysis shows knockdown of PARP1 in MDA231 cells. β-Actin was used as a loading marker. (**J**) PARP1 was depleted in RBM6-deficient MDA231 cells and PARP inhibition effect was measured by CellTiter 96^®^ proliferation assay. Data presented are mean of three independent experiments ± SD. *P< 0.01, **P < 0.001, ***P < 0.0001, ^****^P < 0.00001. All data presented are mean of three independent experiments ± SD.

Tumor cells with mutation in BRCA1 or BRCA2 genes exhibit defective HR (referred to as BRCAness phenotype) and are pronounceably hypersensitive to ATM and PARP inhibition (83–85). Since RBM6-deficient cells are also defective in HR (Figure 2), we sought to determine the sensitivity of RBM6-deficient cells to ATM and PARP inhibitors (ATMi, PARPi). To test this, control and MCF10A^RBM6-KO1^ cells were treated with increasing concentrations of ATMi and subjected to a short-term growth delay assay. Results show that MCF10A^RBM6-KO1^ cells are pronouncedly more sensitive to ATMi when compared to control cells (Figure 7A). To further substantiate this finding, we checked the sensitivity of RBM6-deficient HeLa cells to ATMi. Similar to MCF10A cells, siRNA knockdown of RBM6 hypersensitizes HeLa cells to ATMi (Supplementary Figure S17A, B). Altogether, our data describe a novel vulnerability of RBM6-deficient cells to ATMi. To investigate the underlying basis of the sensitivity of RBM6 deficiency to ATMi, we tested the integrity of HR in RBM6 deficient cells upon ATM inhibition. Results show that ATMi treatment further aggravated the reduction in HR efficiency of RBM6-deficient cells. Moreover, the overall HR efficiency in RBM6-deficient cells treated with ATMi is lower than in control treated cells (Figure 7B). This suggests that the exacerbation of HR deficiency in RBM6-depleted cells treated with ATMi may contribute to the hypersensitivity of RBM6-deficient cells to ATMi. This observation is in line with a previous report showing that ATM inhibition aggravates the reduction in HR in BRCA1-deficient cells and contributes to the synthetic lethality between BRCA1 and ATM (83).

Next, control and RBM6 knockout MCF10A cells were treated with increasing concentrations of PARPi (olaparib; granted FDA approval) and subjected to a short-term growth delay assay. Results show that MCF10A^RBM6-KO1^ and MCF10A^RBM6-KO2^ cells are pronouncedly more sensitive to PARPi compared to control cells (Figure 7C). Similarly, RBM6 depletion in MCF10A using two different sequences of shRNA sensitizes cells to PARPi (Figure 7D). Moreover, the hypersensitivity to PARPi was also observed in the triple negative breast cancer cell line MDA231 that represents highly aggressive and difficult to treat tumors (Figure 7E, F). In addition, we determined the sensitivity of MCF7 breast cancer cell line devoid of RBM6 to PARPi that were transduced with lentiviral vectors expressing either RBM6, Fe65, or empty vector. Results show that MCF7 cells expressing RBM6 and Fe65 are pronouncedly more resistant to PARPi when compared to MCF7 cells transduced with empty vector (Figure 7G). Altogether, our data suggest that re-expression of either RBM6 or Fe65 in MCF7 is sufficient to restore the integrity of HR of DSBs and consequently confer resistance to PARPi. The sensitivity to PARPi was also evident in RBM6-deficient HeLa cells (Supplementary Figure S17C), suggesting that the ‘BRCAness’ phenotype of RBM6-deficient cells displays synthetic lethal interaction with PARPi in a broader range of cancer cells. Collectively, our data describe a novel vulnerability of RBM6-deficient cells to PARP inhibition that can be therapeutically exploited to treat tumors harboring RBM6 mutations.

It was previously shown that the increased cell sensitivity to PARP inhibition requires formation of trapped PARP1–DNA adducts (86,87). On this basis, we proposed that the hypersensitivity of RBM6-deficient cells to PARPi could potentially arise from enhanced PARP trapping on chromatin. To explore this proposition, we quantified PARP1 trapping in control and RBM6-deficient cells upon treatment with PARPi before and after DNA damage. Results show that RBM6-deficient cells show elevated levels of PARP trapping compared to control cells. Notably, PARP trapping in RBM6-deficient cells was further exacerbated following DNA damage induction (Figure 7H). On this basis, we assume that the hypersensitivity of RBM6-deficient cells to PARPi arises from the increase in PARP1 trapping and DNA damage. In support of this assumption, cell survival assay shows that PARP1 depletion suppresses the hypersensitivity of RBM6-deficient cells to PARPi (Figure 7I, J). These results provide supporting evidence that the sensitivity of RBM6-deficient cells to PARPi is mediated, at least in part, by increased PARP1 trapping on DNA.

### Cisplatin sensitizes RBM6-deficient tumors

**Figure 8.**
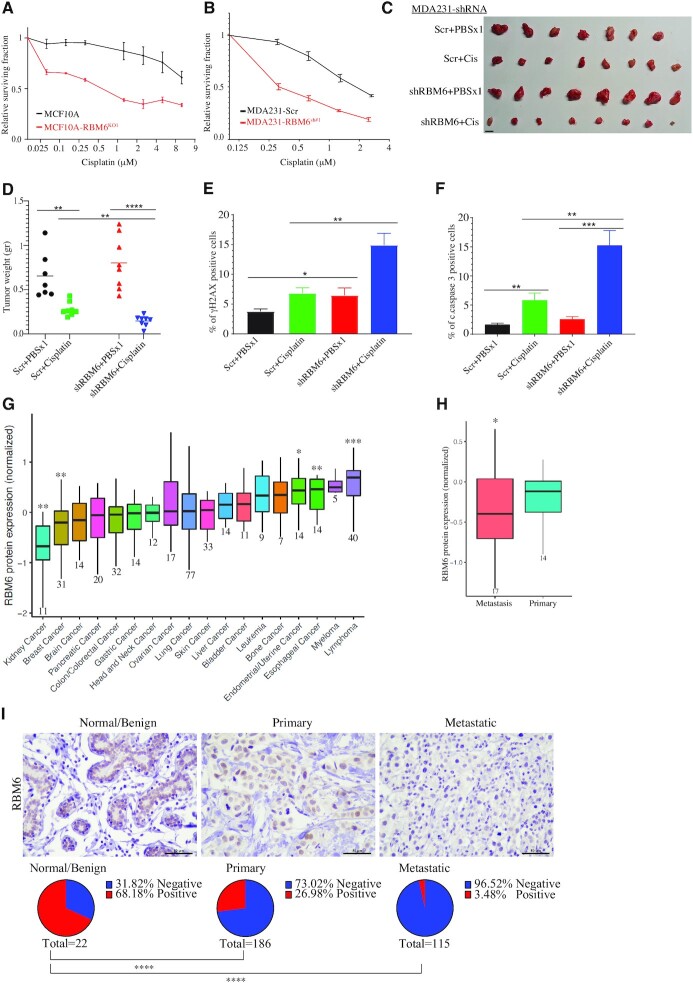
RBM6 deficiency is correlated with breast cancer progression and confers sensitivity to cisplatin in mice xenograft. (**A**, **B**) Cisplatin treatment hypersensitizes MCF10A RBM6-KO1 (A) and MDA231 RBM6-shRNA#1 (B) cells. Cell viability was assessed by the CellTiter 96^®^ proliferation assay. Data presented are mean of three independent experiments ± SD. (**C**, **D**) MDA231 xenograft tumor model. 2.5 × 10^6^ MDA231 cells expressing either scramble or shRNA against RBM6 (RBM6 shRNA#1) were injected subcutaneously. When tumors reached ∼100 mm^3^, mice were assigned randomly to either control (vehicle: PBSx1) or cisplatin (5 mg/kg in PBSx1) treatment (*n* = 8 for each group). Mice were euthanized when the tumors of control mice reached ∼1500 mm^3^. *Left*: Tumors were collected and photographed. Scale bar, 1 cm. *Right*: Average tumor weight ± SD are shown. (**E**, **F**) γH2AX and cleaved caspase 3 (c.caspase 3) in control and RBM6-deficient MDA231 xenografts. Graph shows the percentage of γH2AX (E) and c.caspase 3 (F) positive cells. Data presented are the mean ± SEM of nine different fields from three different tumor sections for each group. (**G**) RBM6 protein expression from quantitative proteome analysis of 375 cancer cell lines from different types of tumors. Data are presented as min to max box plot of the relative protein expression compared to the mean protein expression across all cancer types. The number of cell lines from each cancer type is indicated below. The statistical significance of the differences in protein expression in the indicated cancer types relative to the mean protein expression was determined using one-sample Wilcoxon test. (**H**) As in (G) except that cell lines representing primary and metastatic breast cancer were used for the analysis. (**I**) Representative images (top) and IHC analysis (bottom) of human breast cancer tissue microarray (TMA) (US Biomax – BR1005b, BR1008b and BR2082c) showing immunohistochemically staining of RBM6 in normal, primary and metastatic breast tissue samples. *P< 0.01, **P < 0.001, ***P < 0.0001, ^****^P < 0.00001.

Platinum drugs, such as cisplatin, cause DNA adducts that can be repaired by various mechanisms including HR (88). Therefore, cells with compromised HR are hypersensitive to cisplatin (89–92). Since RBM6 deficiency impairs HR, we sought to examine the sensitivity of RBM6-deficient MCF10A and metastatic breast cancer cell line, MDA231, to cisplatin. We found that RBM6 depletion displays substantial hypersensitivity to cisplatin both in MCF10A and MDA231 cells (Figure 8A-B). Since RBM6-deficient cells show the greatest sensitivity to cisplatin and since cisplatin is a widely used chemotherapeutic drug, we sought to test the *in vivo* relevance of our cellular findings, by determining the effect of cisplatin administration on the growth of MDA231 cells expressing either scramble or RBM6-shRNA in subcutaneous mouse xenograft model. Our results revealed that RBM6-deficient tumors are hypersensitive to cisplatin when compared to RBM6-proficient tumors, as the average weight of the RBM6-deficient tumors in mice treated with cisplatin is significantly lower than cisplatin-treated control tumors (Figure 8C, D). Interestingly, we observed that RBM6-deficient tumors tend to be larger than control tumors supporting its role as a tumor suppressor (Figure 8C, D). Immunostaining analysis showed that cisplatin-treated RBM6-deficient MDA231 tumors exhibit elevated levels of γH2AX likely because of the defective HR in cell devoid of RBM6 (Figure 8E and Supplementary Figure S18). Moreover, RBM6-deficient tumors show increases apoptosis following cisplatin treatment as evident by cleaved caspases 3 staining (Figure 8F and Supplementary Figure S19). In agreement with this, immunohistochemical expression analysis for Ki-67 proliferation marker showed that cisplatin treatment of RBM6-deficient tumors resulted in a decrease in Ki-67 expression relative to vehicle-treated control (Supplementary Figure S20). Altogether, our findings provide the basis for using cisplatin as a promising new therapy of RBM6-deficient cancer cells.

### Low RBM6 expression correlates with human breast cancer metastasis and poor prognosis

Prompted by our previous findings, we sought to examine their clinical relevance in breast cancer. A recent quantitative proteome of 375 cancer cell lines revealed low levels of RBM6 protein in breast cancer cell lines when compared to other types of cancer (93) (Figure 8G). Moreover, we observed that RBM6 protein levels are downregulated in metastatic breast cancer cell lines when compared primary breast cancer cell lines (93) (Figure 8H). Assessment of RBM6 expression in breast cancer tissues from the human protein atlas (https://www.proteinatlas.org/) suggests that RBM6 is downregulated in late and invasive breast cancer. To further corroborate this, we performed immunohistochemical staining of RBM6 in human tissue microarrays (TMA) containing hyperplasia, invasive carcinoma of no special type and metastatic breast carcinoma. We found that RBM6 is drastically downregulated in metastatic carcinoma when compared to invasive carcinoma and hyperplasia, irrespective of breast cancer subtype (Figure 8I and Supplementary Figure S21). These findings substantiate the emerging tumor suppressor activity of RBM6 and suggest it as a potential biomarker for breast cancer progression.

## DISCUSSION

RBM6 regulates alternative splicing as it promotes exon skipping by enhancing the function of the distal splice sites, and consequently substantial number of alternative splicing events are affected by RBM6 depletion in HeLa cells (43). Yet, it remains possible that RBM6 may also function as a constitutive splicing factor. Herein, we identified a novel role of the RRM-containing protein, RBM6, in regulating DSB repair, as its depletion diminishes the efficiency of HR but not NHEJ, which may explain the modest increase in γH2AX levels seen in RBM6-deficient cells. Our findings further support the emerging role of the RRM-containing protein family in orchestrating DNA repair pathways and regulating genome integrity (20,23). Indeed, a growing number of RRM-containing proteins have been implicated in DSB repair including hnRNP (94), RBM14 (95,96), NELF-E (9), FUS (17,97) and NONO (26,98).

Mechanistically, we demonstrated that RBM6 regulates DSB repair through its canonical function by regulating alternative splicing of Fe65. This finding is line with previous reports that identified a growing number of splicing factors that regulate DNA repair by modulating alternative splicing of DDR genes (24). For examples: (1) SRSF6 splicing factor regulates alternative splicing of key HR repair genes such as MCM8 and MDC1 (99). (2) SRSF3 regulates the splicing of the HR factor KMT2C H3K4-specific histone methyltransferase. Moreover, SRSF3 regulates HR by promoting the expression of BRCA1, BRIP1 and RAD51 genes (100,101). (3) SF3B splicing factor and the DNA repair protein, CtIP, control the splicing of hundreds genes, including HR genes such as PIF1 helicase during DDR (102–104).

While this study showed that RBM6 regulates NSD of Fe65 mRNA, previous report did not identify Fe65 as a splicing target of RBM6 (43), presumably due to the low abundance and/or the instability of the Fe65 NSD variant. Nonetheless, it is worth noting that RBM6 may exert its function in regulating HR repair of DSB through alternate pathways other than the regulation of Fe65 levels. In support of this, overexpression of Fe65 elevated HR efficiency to a lesser extent than RBM6 overexpression (Figure 6E) suggesting that RBM6 possesses additional functions in regulating HR. Concordantly, our RNA-seq data showed that other DNA damage responsive proteins are downregulated in RBM6-deficient cells and their impact on DNA repair needs to be elucidated. In addition, we cannot rule out an additional and not mutually exclusive possibility that RBM6 has a direct role in DNA repair. Indeed, several RRM-containing proteins were shown to be recruited to DNA damage sites supporting a direct role in DNA repair (9,26,97,105).

Several surveillance mechanisms for monitoring mRNA translation were identified including nonsense-mediated mRNA decay (NMD), no-go decay (NGD), and non-stop decay (NSD) (57,76,106–108). Here, we implicated, for the first time, RBM6 in regulating HR of DSBs via regulating alternative splicing-coupled NSD of Fe65. To the best of our knowledge, RBM6 is the first RRM-containing protein that regulates AS-NSD and future studies will be essential to determine whether other RRM-containing proteins possess similar functions. This study also identified RNPS1 as one of the most enriched proximity interactors of RBM6 and unprecedently implicated it in regulating Fe65 NSD variant. Notably, while RNPS1 is involved in splicing, mRNA export and NMD (80,81), our findings suggests that RNPS1 may also regulate AS-NSD of Fe65 mRNA. Further work will be required to address whether RBM6 and RNPS1 regulate AS-NSD of target genes other than Fe65 and whether they have common target genes. Beside the role of RBM6 in regulating AS-NSD of Fe65 exon 15, it is possible that RBM6 regulates the general splicing efficiency of Fe65, as evidenced by the decreased ratio between the spliced and the unspliced Fe65 intron 7 in RBM6-defcient cells compared to control cells (Figure 3L and S8). This might lead to the generation of aberrant Fe65 transcripts that are targeted for decay. Moreover, it is worth assessing whether RBM6 regulates Fe65 levels through additional post-transcriptional processes besides splicing.

Previous works suggested that RBM6 exhibits tumor suppressor activity (43–46). Concordantly, we demonstrated that RBM6 level is downregulated in invasive breast cancer (Figure 8H, I). The molecular mechanism underlying the tumor suppressor activity of RBM6 remains elusive. We propose that RBM6 role in DSB repair may contribute, at least in part, to its tumor suppressor function. Also, since RBM6 depletion decreases error-free HR repair, but not the error-prone NHEJ, of DSB (Figure 2A, B), its downregulation may lead to accumulation of mutations and genomic instabilities fueling carcinogenesis.

Our results demonstrate that RBM6-deficient cells are hypersensitive to IR, PARPi, ATMi or cisplatin. Additionally, cisplatin treatment inhibited the growth of tumor devoid of RBM6 in mouse xenograft model. Our data show that the hypersensitivity of RBM6-deficient cells to the aforementioned chemotherapeutic drugs is not restricted to breast cancer cell lines. Interestingly, in addition to breast and lung cancer, RBM6 is mutated in 8.8% of uterine and in 7.2% of stomach cancer (47–49). We therefore propose that cisplatin administration might be effective in eradicating various types of tumors harboring RBM6 mutations.

## DATA AVAILABILITY

RNA-seq data have been submitted to GEO database: GSE167265. Mass spectrometry data have been submitted to PRIDE database: PXD024295.

## Supplementary Material

gkab976_Supplemental_Files

## References

[1] Huang L.C. , Clarkin K.C., Wahl G.M. Sensitivity and selectivity of the DNA damage sensor responsible for activating p53-dependent G1 arrest. Proc. Natl. Acad. Sci. U.S.A. 1996; 93:4827–4832. 8643488 10.1073/pnas.93.10.4827 PMC39364

[2] Bennett C.B. , Lewis A.L., Baldwin K.K., Resnick M.A. Lethality induced by a single site-specific double-strand break in a dispensable yeast plasmid. Proc. Natl. Acad. Sci. U.S.A. 1993; 90:5613–5617. 8516308 10.1073/pnas.90.12.5613 PMC46771

[3] Rich T. , Allen R.L., Wyllie A.H. Defying death after DNA damage. Nature. 2000; 407:777–783. 11048728 10.1038/35037717

[4] Hartlerode A.J. , Scully R. Mechanisms of double-strand break repair in somatic mammalian cells. Biochem. J. 2009; 423:157–168. 19772495 10.1042/BJ20090942 PMC2983087

[5] Chapman J.R. , Taylor M.R., Boulton S.J. Playing the end game: DNA double-strand break repair pathway choice. Mol. Cell. 2012; 47:497–510. 22920291 10.1016/j.molcel.2012.07.029

[6] Hustedt N. , Durocher D. The control of DNA repair by the cell cycle. Nat. Cell Biol. 2016; 19:1–9. 28008184 10.1038/ncb3452

[7] Jasin M. , Rothstein R. Repair of strand breaks by homologous recombination. Cold Spring Harb. Perspect. Biol. 2013; 5:a012740. 24097900 10.1101/cshperspect.a012740 PMC3809576

[8] Chiruvella K.K. , Liang Z., Wilson T.E. Repair of double-strand breaks by end joining. Cold Spring Harb. Perspect. Biol. 2013; 5:a012757. 23637284 10.1101/cshperspect.a012757 PMC3632057

[9] Awwad S.W. , Abu-Zhayia E.R., Guttmann-Raviv N., Ayoub N. NELF-E is recruited to DNA double-strand break sites to promote transcriptional repression and repair. EMBO Rep. 2017; 18:745–764. 28336775 10.15252/embr.201643191 PMC5412775

[10] Abu-Zhayia E.R. , Khoury-Haddad H., Guttmann-Raviv N., Serruya R., Jarrous N., Ayoub N. A role of human RNase P subunits, Rpp29 and Rpp21, in homology directed-repair of double-strand breaks. Sci. Rep. 2017; 7:1002. 28432356 10.1038/s41598-017-01185-6 PMC5430778

[11] Khoury-Haddad H. , Nadar-Ponniah P.T., Awwad S., Ayoub N. The emerging role of lysine demethylases in DNA damage response: dissecting the recruitment mode of KDM4D/JMJD2D to DNA damage sites. Cell Cycle. 2015; 14:950–958. 25714495 10.1080/15384101.2015.1014147 PMC4614868

[12] Zoabi M. , Nadar-Ponniah P.T., Khoury-Haddad H., Usaj M., Budowski-Tal I., Haran T., Henn A., Mandel-Gutfreund Y., Ayoub N. RNA-dependent chromatin localization of KDM4D lysine demethylase promotes H3K9me3 demethylation. Nucleic Acids Res. 2014; 42:13026–13038. 25378304 10.1093/nar/gku1021 PMC4245933

[13] Khoury-Haddad H. , Guttmann-Raviv N., Ipenberg I., Huggins D., Jeyasekharan A.D., Ayoub N. PARP1-dependent recruitment of KDM4D histone demethylase to DNA damage sites promotes double-strand break repair. Proc. Natl. Acad. Sci. U.S.A. 2014; 111:E728–E737. 24550317 10.1073/pnas.1317585111 PMC3932863

[14] Milek M. , Imami K., Mukherjee N., Bortoli F., Zinnall U., Hazapis O., Trahan C., Oeffinger M., Heyd F., Ohler U. et al. DDX54 regulates transcriptome dynamics during DNA damage response. Genome Res. 2017; 27:1344–1359. 28596291 10.1101/gr.218438.116 PMC5538551

[15] Montecucco A. , Biamonti G. Pre-mRNA processing factors meet the DNA damage response. Front Genet. 2013; 4:102. 23761808 10.3389/fgene.2013.00102 PMC3674313

[16] Adamson B. , Smogorzewska A., Sigoillot F.D., King R.W., Elledge S.J. A genome-wide homologous recombination screen identifies the RNA-binding protein RBMX as a component of the DNA-damage response. Nat. Cell Biol. 2012; 14:318–328. 22344029 10.1038/ncb2426 PMC3290715

[17] Mastrocola A.S. , Kim S.H., Trinh A.T., Rodenkirch L.A., Tibbetts R.S. The RNA-binding protein fused in sarcoma (FUS) functions downstream of poly(ADP-ribose) polymerase (PARP) in response to DNA damage. J. Biol. Chem. 2013; 288:24731–24741. 23833192 10.1074/jbc.M113.497974 PMC3750169

[18] Nishida K. , Kuwano Y., Nishikawa T., Masuda K., Rokutan K. RNA binding proteins and genome integrity. Int. J. Mol. Sci. 2017; 18:10.3390/ijms18071341. PMC5535834 28644387

[19] Hawley B.R. , Lu W.T., Wilczynska A., Bushell M. The emerging role of RNAs in DNA damage repair. Cell Death Differ. 2017; 24:580–587. 28234355 10.1038/cdd.2017.16 PMC5384027

[20] Kai M. Roles of RNA-binding proteins in DNA damage response. Int. J. Mol. Sci. 2016; 17:310. 27110771 10.3390/ijms17040604 PMC4849055

[21] Giono L.E. , Nieto Moreno N., Cambindo Botto A.E., Dujardin G., Munoz M.J., Kornblihtt A.R. The RNA response to DNA damage. J. Mol. Biol. 2016; 428:2636–2651. 26979557 10.1016/j.jmb.2016.03.004

[22] Dutertre M. , Vagner S. DNA-damage response RNA-binding proteins (DDRBPs): perspectives from a new class of proteins and their RNA targets. J. Mol. Biol. 2017; 429:3139–3145. 27693651 10.1016/j.jmb.2016.09.019

[23] Klaric J.A. , Wust S., Panier S. New faces of old friends: emerging new roles of RNA-binding proteins in the DNA double-strand break response. Front. Mol. Biosci. 2021; 8:668821. 34026839 10.3389/fmolb.2021.668821 PMC8138124

[24] Shkreta L. , Chabot B. The RNA splicing response to DNA damage. Biomolecules. 2015; 5:2935–2977. 26529031 10.3390/biom5042935 PMC4693264

[25] Naro C. , Bielli P., Pagliarini V., Sette C. The interplay between DNA damage response and RNA processing: the unexpected role of splicing factors as gatekeepers of genome stability. Front Genet. 2015; 6:142. 25926848 10.3389/fgene.2015.00142 PMC4397863

[26] Salton M. , Lerenthal Y., Wang S.Y., Chen D.J., Shiloh Y. Involvement of Matrin 3 and SFPQ/NONO in the DNA damage response. Cell Cycle. 2010; 9:1568–1576. 20421735 10.4161/cc.9.8.11298

[27] Marchesini M. , Ogoti Y., Fiorini E., Aktas Samur A., Nezi L., D’Anca M., Storti P., Samur M.K., Ganan-Gomez I., Fulciniti M.T. et al. ILF2 is a regulator of RNA splicing and DNA damage response in 1q21-amplified multiple myeloma. Cancer Cell. 2017; 32:88–100. 28669490 10.1016/j.ccell.2017.05.011 PMC5593798

[28] Kochan J.A. , Desclos E.C.B., Bosch R., Meister L., Vriend L.E.M., van Attikum H., Krawczyk P.M. Meta-analysis of DNA double-strand break response kinetics. Nucleic Acids Res. 2017; 45:12625–12637. 29182755 10.1093/nar/gkx1128 PMC5728399

[29] Salas-Armenteros I. , Barroso S.I., Rondon A.G., Perez M., Andujar E., Luna R., Aguilera A. Depletion of the MFAP1/SPP381 splicing factor causes R-loop-independent genome instability. Cell Rep. 2019; 28:1551–1563. 31390568 10.1016/j.celrep.2019.07.010 PMC6693559

[30] Cloutier A. , Shkreta L., Toutant J., Durand M., Thibault P., Chabot B. hnRNP A1/A2 and Sam68 collaborate with SRSF10 to control the alternative splicing response to oxaliplatin-mediated DNA damage. Sci. Rep. 2018; 8:2206. 29396485 10.1038/s41598-018-20360-x PMC5797138

[31] Shkreta L. , Toutant J., Durand M., Manley J.L., Chabot B. SRSF10 connects DNA damage to the alternative splicing of transcripts encoding apoptosis, cell-cycle control, and DNA repair factors. Cell Rep. 2016; 17:1990–2003. 27851963 10.1016/j.celrep.2016.10.071 PMC5483951

[32] Savage K.I. , Gorski J.J., Barros E.M., Irwin G.W., Manti L., Powell A.J., Pellagatti A., Lukashchuk N., McCance D.J., McCluggage W.G. et al. Identification of a BRCA1-mRNA splicing complex required for efficient DNA repair and maintenance of genomic stability. Mol. Cell. 2014; 54:445–459. 24746700 10.1016/j.molcel.2014.03.021 PMC4017265

[33] Martinez-Montiel N. , Rosas-Murrieta N.H., Anaya Ruiz M., Monjaraz-Guzman E., Martinez-Contreras R. Alternative splicing as a target for cancer treatment. Int. J. Mol. Sci. 2018; 19:545. 10.3390/ijms19020545 PMC5855767 29439487

[34] Du L. , Gatti R.A. Progress toward therapy with antisense-mediated splicing modulation. Curr. Opin. Mol. Ther. 2009; 11:116–123. 19330717 PMC2753608

[35] Zammarchi F. , de Stanchina E., Bournazou E., Supakorndej T., Martires K., Riedel E., Corben A.D., Bromberg J.F., Cartegni L. Antitumorigenic potential of STAT3 alternative splicing modulation. Proc. Natl. Acad. Sci. U.S.A. 2011; 108:17779–17784. 22006329 10.1073/pnas.1108482108 PMC3203802

[36] Hsu T.Y. , Simon L.M., Neill N.J., Marcotte R., Sayad A., Bland C.S., Echeverria G.V., Sun T., Kurley S.J., Tyagi S. et al. The spliceosome is a therapeutic vulnerability in MYC-driven cancer. Nature. 2015; 525:384–388. 26331541 10.1038/nature14985 PMC4831063

[37] Jbara A. , Siegfried Z., Karni R. Splice-switching as cancer therapy. Curr. Opin. Pharmacol. 2021; 59:140–148. 34217945 10.1016/j.coph.2021.05.008

[38] Martin B.T. , Serrano P., Geralt M., Wuthrich K. Nuclear magnetic resonance structure of a novel globular domain in RBM10 containing OCRE, the octamer repeat sequence motif. Structure. 2016; 24:158–164. 26712279 10.1016/j.str.2015.10.029 PMC4706790

[39] Mourao A. , Bonnal S., Soni K., Warner L., Bordonne R., Valcarcel J., Sattler M. Structural basis for the recognition of spliceosomal SmN/B/B' proteins by the RBM5 OCRE domain in splicing regulation. eLife. 2016; 5:e14707. 27894420 10.7554/eLife.14707 PMC5127646

[40] Callebaut I. , Mornon J.P. OCRE: a novel domain made of imperfect, aromatic-rich octamer repeats. Bioinformatics. 2005; 21:699–702. 15486042 10.1093/bioinformatics/bti065

[41] Inoue A. RBM10: structure, functions, and associated diseases. Gene. 2021; 783:145463. 33515724 10.1016/j.gene.2021.145463 PMC10445532

[42] Heath E. , Sablitzky F., Morgan G.T. Subnuclear targeting of the RNA-binding motif protein RBM6 to splicing speckles and nascent transcripts. Chromosome Res. 2010; 18:851–872. 21086038 10.1007/s10577-010-9170-7

[43] Bechara E.G. , Sebestyen E., Bernardis I., Eyras E., Valcarcel J. RBM5, 6, and 10 differentially regulate NUMB alternative splicing to control cancer cell proliferation. Mol. Cell. 2013; 52:720–733. 24332178 10.1016/j.molcel.2013.11.010

[44] Wang Q. , Wang F., Zhong W., Ling H., Wang J., Cui J., Xie T., Wen S., Chen J. RNA-binding protein RBM6 as a tumor suppressor gene represses the growth and progression in laryngocarcinoma. Gene. 2019; 697:26–34. 30772516 10.1016/j.gene.2019.02.025

[45] Wistuba II , Behrens C., Virmani A.K., Mele G., Milchgrub S., Girard L., Fondon J.W. 3rd, Garner H.R., McKay B., Latif F. et al. High resolution chromosome 3p allelotyping of human lung cancer and preneoplastic/preinvasive bronchial epithelium reveals multiple, discontinuous sites of 3p allele loss and three regions of frequent breakpoints. Cancer Res. 2000; 60:1949–1960. 10766185

[46] Angeloni D. Molecular analysis of deletions in human chromosome 3p21 and the role of resident cancer genes in disease. Brief. Funct. Genomic. Proteomic. 2007; 6:19–39. 17525073 10.1093/bfgp/elm007

[47] Gao J. , Aksoy B.A., Dogrusoz U., Dresdner G., Gross B., Sumer S.O., Sun Y., Jacobsen A., Sinha R., Larsson E. et al. Integrative analysis of complex cancer genomics and clinical profiles using the cBioPortal. Sci. Signal. 2013; 6:pl1. 23550210 10.1126/scisignal.2004088 PMC4160307

[48] Cerami E. , Gao J., Dogrusoz U., Gross B.E., Sumer S.O., Aksoy B.A., Jacobsen A., Byrne C.J., Heuer M.L., Larsson E. et al. The cBio cancer genomics portal: an open platform for exploring multidimensional cancer genomics data. Cancer Discov. 2012; 2:401–404. 22588877 10.1158/2159-8290.CD-12-0095 PMC3956037

[49] Cancer Genome Atlas Research, N. Weinstein J.N. , Collisson E.A., Mills G.B., Shaw K.R., Ozenberger B.A., Ellrott K., Shmulevich I., Sander C., Stuart J.M. The Cancer Genome Atlas Pan-Cancer analysis project. Nat. Genet. 2013; 45:1113–1120. 24071849 10.1038/ng.2764 PMC3919969

[50] Guo Y. , Updegraff B.L., Park S., Durakoglugil D., Cruz V.H., Maddux S., Hwang T.H., O’Donnell K.A. Comprehensive ex vivo transposon mutagenesis identifies genes that promote growth factor independence and leukemogenesis. Cancer Res. 2016; 76:773–786. 26676752 10.1158/0008-5472.CAN-15-1697

[51] Rangel R. , Lee S.C., Hon-Kim Ban K., Guzman-Rojas L., Mann M.B., Newberg J.Y., Kodama T., McNoe L.A., Selvanesan L., Ward J.M. et al. Transposon mutagenesis identifies genes that cooperate with mutant Pten in breast cancer progression. Proc. Natl. Acad. Sci. U.S.A. 2016; 113:E7749–E7758. 27849608 10.1073/pnas.1613859113 PMC5137755

[52] Takeda H. , Wei Z., Koso H., Rust A.G., Yew C.C., Mann M.B., Ward J.M., Adams D.J., Copeland N.G., Jenkins N.A. Transposon mutagenesis identifies genes and evolutionary forces driving gastrointestinal tract tumor progression. Nat. Genet. 2015; 47:142–150. 25559195 10.1038/ng.3175

[53] Morris S.M. , Davison J., Carter K.T., O’Leary R.M., Trobridge P., Knoblaugh S.E., Myeroff L.L., Markowitz S.D., Brett B.T., Scheetz T.E. et al. Transposon mutagenesis identifies candidate genes that cooperate with loss of transforming growth factor-beta signaling in mouse intestinal neoplasms. Int. J. Cancer. 2017; 140:853–863. 27790711 10.1002/ijc.30491 PMC5316486

[54] Bard-Chapeau E.A. , Nguyen A.T., Rust A.G., Sayadi A., Lee P., Chua B.Q., New L.S., de Jong J., Ward J.M., Chin C.K. et al. Transposon mutagenesis identifies genes driving hepatocellular carcinoma in a chronic hepatitis B mouse model. Nat. Genet. 2014; 46:24–32. 24316982 10.1038/ng.2847 PMC4131144

[55] Kodama T. , Bard-Chapeau E.A., Newberg J.Y., Kodama M., Rangel R., Yoshihara K., Ward J.M., Jenkins N.A., Copeland N.G. Two-step forward genetic screen in mice identifies Ral GTPase-activating proteins as suppressors of hepatocellular carcinoma. Gastroenterology. 2016; 151:324–337. 27178121 10.1053/j.gastro.2016.04.040

[56] Duan B. , Hu X., Fan M., Xiong X., Han L., Wang Z., Tong D., Liu L., Wang X., Li W. et al. RNA-binding motif protein 6 is a candidate serum biomarker for pancreatic cancer. Proteomics Clin Appl. 2019; 13:e1900048. 31207145 10.1002/prca.201900048

[57] Powers K.T. , Szeto J.A., Schaffitzel C. New insights into no-go, non-stop and nonsense-mediated mRNA decay complexes. Curr. Opin. Struct. Biol. 2020; 65:110–118. 32688260 10.1016/j.sbi.2020.06.011

[58] Pinder J. , Salsman J., Dellaire G. Nuclear domain ‘knock-in’ screen for the evaluation and identification of small molecule enhancers of CRISPR-based genome editing. Nucleic Acids Res. 2015; 43:9379–9392. 26429972 10.1093/nar/gkv993 PMC4627099

[59] Mansour W.Y. , Schumacher S., Rosskopf R., Rhein T., Schmidt-Petersen F., Gatzemeier F., Haag F., Borgmann K., Willers H., Dahm-Daphi J. Hierarchy of nonhomologous end-joining, single-strand annealing and gene conversion at site-directed DNA double-strand breaks. Nucleic Acids Res. 2008; 36:4088–4098. 18539610 10.1093/nar/gkn347 PMC2475611

[60] Love M.I. , Huber W., Anders S. Moderated estimation of fold change and dispersion for RNA-seq data with DESeq2. Genome Biol. 2014; 15:550. 25516281 10.1186/s13059-014-0550-8 PMC4302049

[61] Shen S. , Park J.W., Lu Z.X., Lin L., Henry M.D., Wu Y.N., Zhou Q., Xing Y. rMATS: robust and flexible detection of differential alternative splicing from replicate RNA-Seq data. Proc. Natl. Acad. Sci. U.S.A. 2014; 111:E5593–E5601. 25480548 10.1073/pnas.1419161111 PMC4280593

[62] Huang da W. , Sherman B.T., Lempicki R.A. Systematic and integrative analysis of large gene lists using DAVID bioinformatics resources. Nat. Protoc. 2009; 4:44–57. 19131956 10.1038/nprot.2008.211

[63] Bennetzen M.V. , Larsen D.H., Bunkenborg J., Bartek J., Lukas J., Andersen J.S. Site-specific phosphorylation dynamics of the nuclear proteome during the DNA damage response. Mol. Cell. Proteomics. 2010; 9:1314–1323. 20164059 10.1074/mcp.M900616-MCP200 PMC2877989

[64] Elia A.E. , Boardman A.P., Wang D.C., Huttlin E.L., Everley R.A., Dephoure N., Zhou C., Koren I., Gygi S.P., Elledge S.J. Quantitative proteomic atlas of ubiquitination and acetylation in the DNA damage response. Mol. Cell. 2015; 59:867–881. 26051181 10.1016/j.molcel.2015.05.006 PMC4560960

[65] Matsuoka S. , Ballif B.A., Smogorzewska A., McDonald E.R. 3rd, Hurov K.E., Luo J., Bakalarski C.E., Zhao Z., Solimini N., Lerenthal Y. et al. ATM and ATR substrate analysis reveals extensive protein networks responsive to DNA damage. Science. 2007; 316:1160–1166. 17525332 10.1126/science.1140321

[66] Ran F.A. , Hsu P.D., Lin C.Y., Gootenberg J.S., Konermann S., Trevino A.E., Scott D.A., Inoue A., Matoba S., Zhang Y. et al. Double nicking by RNA-guided CRISPR Cas9 for enhanced genome editing specificity. Cell. 2013; 154:1380–1389. 23992846 10.1016/j.cell.2013.08.021 PMC3856256

[67] Stante M. , Minopoli G., Passaro F., Raia M., Vecchio L.D., Russo T. Fe65 is required for Tip60-directed histone H4 acetylation at DNA strand breaks. Proc. Natl. Acad. Sci. U.S.A. 2009; 106:5093–5098. 19282473 10.1073/pnas.0810869106 PMC2664056

[68] Szumiel I. , Foray N. Chromatin acetylation, beta-amyloid precursor protein and its binding partner FE65 in DNA double strand break repair. Acta Biochim. Pol. 2011; 58:11–18. 21403922

[69] Liu Y. , Long Y.H., Wang S.Q., Li Y.F., Zhang J.H. Phosphorylation of H2A.X(T)(yr39) positively regulates DNA damage response and is linked to cancer progression. FEBS J. 2016; 283:4462–4473. 27813335 10.1111/febs.13951

[70] Wei D. , Parsels L.A., Karnak D., Davis M.A., Parsels J.D., Marsh A.C., Zhao L., Maybaum J., Lawrence T.S., Sun Y. et al. Inhibition of protein phosphatase 2A radiosensitizes pancreatic cancers by modulating CDC25C/CDK1 and homologous recombination repair. Clin, Cancer Res. 2013; 19:4422–4432. 23780887 10.1158/1078-0432.CCR-13-0788 PMC3754450

[71] Ryu S. , Teles F., Minopoli G., Russo T., Rosenfeld M.G., Suh Y. An epigenomic role of Fe65 in the cellular response to DNA damage. Mutat. Res. 2015; 776:40–47. 26255939 10.1016/j.mrfmmm.2015.01.006 PMC4531264

[72] Ghandi M. , Huang F.W., Jane-Valbuena J., Kryukov G.V., Lo C.C., McDonald E.R. 3rd, Barretina J., Gelfand E.T., Bielski C.M., Li H. et al. Next-generation characterization of the Cancer Cell Line Encyclopedia. Nature. 2019; 569:503–508. 31068700 10.1038/s41586-019-1186-3 PMC6697103

[73] Hashimoto Y. , Takahashi M., Sakota E., Nakamura Y. Nonstop-mRNA decay machinery is involved in the clearance of mRNA 5′-fragments produced by RNAi and NMD in Drosophila melanogaster cells. Biochem. Biophys. Res. Commun. 2017; 484:1–7. 28115162 10.1016/j.bbrc.2017.01.092

[74] Szadeczky-Kardoss I. , Gal L., Auber A., Taller J., Silhavy D. The No-go decay system degrades plant mRNAs that contain a long A-stretch in the coding region. Plant Sci. 2018; 275:19–27. 30107878 10.1016/j.plantsci.2018.07.008

[75] Szadeczky-Kardoss I. , Csorba T., Auber A., Schamberger A., Nyiko T., Taller J., Orban T.I., Burgyan J., Silhavy D. The nonstop decay and the RNA silencing systems operate cooperatively in plants. Nucleic Acids Res. 2018; 46:4632–4648. 29672715 10.1093/nar/gky279 PMC5961432

[76] Wolin S.L. , Maquat L.E. Cellular RNA surveillance in health and disease. Science. 2019; 366:822–827. 31727827 10.1126/science.aax2957 PMC6938259

[77] Ikeuchi K. , Yazaki E., Kudo K., Inada T. Conserved functions of human Pelota in mRNA quality control of nonstop mRNA. FEBS Lett. 2016; 590:3254–3263. 27543824 10.1002/1873-3468.12366

[78] Arribere J.A. , Fire A.Z. Nonsense mRNA suppression via nonstop decay. eLife. 2018; 7:e33292. 29309033 10.7554/eLife.33292 PMC5777819

[79] Rhee H.W. , Zou P., Udeshi N.D., Martell J.D., Mootha V.K., Carr S.A., Ting A.Y. Proteomic mapping of mitochondria in living cells via spatially restricted enzymatic tagging. Science. 2013; 339:1328–1331. 23371551 10.1126/science.1230593 PMC3916822

[80] Lykke-Andersen J. , Shu M.D., Steitz J.A. Communication of the position of exon-exon junctions to the mRNA surveillance machinery by the protein RNPS1. Science. 2001; 293:1836–1839. 11546874 10.1126/science.1062786

[81] Wilkinson M.F. A new function for nonsense-mediated mRNA-decay factors. Trends Genet. 2005; 21:143–148. 15734573 10.1016/j.tig.2005.01.007

[82] Minopoli G. , Stante M., Napolitano F., Telese F., Aloia L., De Felice M., Di Lauro R., Pacelli R., Brunetti A., Zambrano N. et al. Essential roles for Fe65, Alzheimer amyloid precursor-binding protein, in the cellular response to DNA damage. J. Biol. Chem. 2007; 282:831–835. 17121854 10.1074/jbc.C600276200

[83] Chen C.C. , Kass E.M., Yen W.F., Ludwig T., Moynahan M.E., Chaudhuri J., Jasin M. ATM loss leads to synthetic lethality in BRCA1 BRCT mutant mice associated with exacerbated defects in homology-directed repair. Proc. Natl. Acad. Sci. U.S.A. 2017; 114:7665–7670. 28659469 10.1073/pnas.1706392114 PMC5530697

[84] Bryant H.E. , Schultz N., Thomas H.D., Parker K.M., Flower D., Lopez E., Kyle S., Meuth M., Curtin N.J., Helleday T. Specific killing of BRCA2-deficient tumours with inhibitors of poly(ADP-ribose) polymerase. Nature. 2005; 434:913–917. 15829966 10.1038/nature03443

[85] Farmer H. , McCabe N., Lord C.J., Tutt A.N., Johnson D.A., Richardson T.B., Santarosa M., Dillon K.J., Hickson I., Knights C. et al. Targeting the DNA repair defect in BRCA mutant cells as a therapeutic strategy. Nature. 2005; 434:917–921. 15829967 10.1038/nature03445

[86] Murai J. , Huang S.Y., Das B.B., Renaud A., Zhang Y., Doroshow J.H., Ji J., Takeda S., Pommier Y. Trapping of PARP1 and PARP2 by Clinical PARP Inhibitors. Cancer Res. 2012; 72:5588–5599. 23118055 10.1158/0008-5472.CAN-12-2753 PMC3528345

[87] Zimmermann M. , Murina O., Reijns M.A.M., Agathanggelou A., Challis R., Tarnauskaite Z., Muir M., Fluteau A., Aregger M., McEwan A. et al. CRISPR screens identify genomic ribonucleotides as a source of PARP-trapping lesions. Nature. 2018; 559:285–289. 29973717 10.1038/s41586-018-0291-z PMC6071917

[88] Dronkert M.L. , Kanaar R. Repair of DNA interstrand cross-links. Mutat. Res. 2001; 486:217–247. 11516927 10.1016/s0921-8777(01)00092-1

[89] Kennedy R.D. , Quinn J.E., Mullan P.B., Johnston P.G., Harkin D.P. The role of BRCA1 in the cellular response to chemotherapy. J. Natl. Cancer Inst. 2004; 96:1659–1668. 15547178 10.1093/jnci/djh312

[90] Horiuchi A. , Wang C., Kikuchi N., Osada R., Nikaido T., Konishi I. BRCA1 expression is an important biomarker for chemosensitivity: suppression of BRCA1 increases the apoptosis via up-regulation of p53 and p21 during cisplatin treatment in ovarian cancer cells. Biomark Insights. 2007; 1:49–59. 19690636 PMC2716781

[91] Nojima K. , Hochegger H., Saberi A., Fukushima T., Kikuchi K., Yoshimura M., Orelli B.J., Bishop D.K., Hirano S., Ohzeki M. et al. Multiple repair pathways mediate tolerance to chemotherapeutic cross-linking agents in vertebrate cells. Cancer Res. 2005; 65:11704–11711. 16357182 10.1158/0008-5472.CAN-05-1214

[92] Tutt A.N. , Lord C.J., McCabe N., Farmer H., Turner N., Martin N.M., Jackson S.P., Smith G.C., Ashworth A. Exploiting the DNA repair defect in BRCA mutant cells in the design of new therapeutic strategies for cancer. Cold Spring Harb. Symp. Quant. Biol. 2005; 70:139–148. 16869747 10.1101/sqb.2005.70.012

[93] Nusinow D.P. , Szpyt J., Ghandi M., Rose C.M., McDonald E.R. 3rd, Kalocsay M., Jane-Valbuena J., Gelfand E., Schweppe D.K., Jedrychowski M. et al. Quantitative Proteomics of the Cancer Cell Line Encyclopedia. Cell. 2020; 180:387–402. 31978347 10.1016/j.cell.2019.12.023 PMC7339254

[94] Hu W. , Lei L., Xie X., Huang L., Cui Q., Dang T., Liu G.L., Li Y., Sun X., Zhou Z. Heterogeneous nuclear ribonucleoprotein L facilitates recruitment of 53BP1 and BRCA1 at the DNA break sites induced by oxaliplatin in colorectal cancer. Cell Death. Dis. 2019; 10:550. 31320608 10.1038/s41419-019-1784-x PMC6639419

[95] Yuan M. , Eberhart C.G., Kai M. RNA binding protein RBM14 promotes radio-resistance in glioblastoma by regulating DNA repair and cell differentiation. Oncotarget. 2014; 5:2820–2826. 24811242 10.18632/oncotarget.1924 PMC4058047

[96] Simon N.E. , Yuan M., Kai M. RNA-binding protein RBM14 regulates dissociation and association of non-homologous end joining proteins. Cell Cycle. 2017; 16:1175–1180. 28426349 10.1080/15384101.2017.1317419 PMC5499918

[97] Wang W.Y. , Pan L., Su S.C., Quinn E.J., Sasaki M., Jimenez J.C., Mackenzie I.R., Huang E.J., Tsai L.H. Interaction of FUS and HDAC1 regulates DNA damage response and repair in neurons. Nat. Neurosci. 2013; 16:1383–1391. 24036913 10.1038/nn.3514 PMC5564396

[98] Krietsch J. , Caron M.C., Gagne J.P., Ethier C., Vignard J., Vincent M., Rouleau M., Hendzel M.J., Poirier G.G., Masson J.Y. PARP activation regulates the RNA-binding protein NONO in the DNA damage response to DNA double-strand breaks. Nucleic Acids Res. 2012; 40:10287–10301. 22941645 10.1093/nar/gks798 PMC3488241

[99] Yang X. , Zhan P., Feng S., Ji H., Tian W., Wang M., Cheng C., Song B. SRSF6 regulates alternative splicing of genes involved in DNA damage response and DNA repair in HeLa cells. Oncol. Rep. 2020; 44:1851–1862. 32901876 10.3892/or.2020.7750 PMC7551351

[100] He X. , Zhang P. Serine/arginine-rich splicing factor 3 (SRSF3) regulates homologous recombination-mediated DNA repair. Mol. Cancer. 2015; 14:158. 26282282 10.1186/s12943-015-0422-1 PMC4539922

[101] Rampias T. , Karagiannis D., Avgeris M., Polyzos A., Kokkalis A., Kanaki Z., Kousidou E., Tzetis M., Kanavakis E., Stravodimos K. et al. The lysine-specific methyltransferase KMT2C/MLL3 regulates DNA repair components in cancer. EMBO Rep. 2019; 20:e46821. 30665945 10.15252/embr.201846821 PMC6399616

[102] Jimeno S. , Camarillo R., Mejias-Navarro F., Fernandez-Avila M.J., Soria-Bretones I., Prados-Carvajal R., Huertas P. The helicase PIF1 facilitates resection over sequences prone to forming G4 structures. Cell Rep. 2018; 24:3262–3273. 30232007 10.1016/j.celrep.2018.08.047

[103] Prados-Carvajal R. , Rodriguez-Real G., Gutierrez-Pozo G., Huertas P. CtIP-mediated alternative mRNA splicing finetunes the DNA damage response. RNA. 2021; 27:303–323. 10.1261/rna.078519.120 PMC7901839 33298529

[104] Prados-Carvajal R. , Lopez-Saavedra A., Cepeda-Garcia C., Jimeno S., Huertas P. Multiple roles of the splicing complex SF3B in DNA end resection and homologous recombination. DNA Repair (Amst.). 2018; 66-67:11–23. 29705135 10.1016/j.dnarep.2018.04.003

[105] Jang Y. , Elsayed Z., Eki R., He S., Du K.P., Abbas T., Kai M. Intrinsically disordered protein RBM14 plays a role in generation of RNA:DNA hybrids at double-strand break sites. Proc. Natl. Acad. Sci. U.S.A. 2020; 117:5329–5338. 32094185 10.1073/pnas.1913280117 PMC7071921

[106] Frischmeyer P.A. , van Hoof A., O’Donnell K., Guerrerio A.L., Parker R., Dietz H.C. An mRNA surveillance mechanism that eliminates transcripts lacking termination codons. Science. 2002; 295:2258–2261. 11910109 10.1126/science.1067338

[107] van Hoof A. , Frischmeyer P.A., Dietz H.C., Parker R. Exosome-mediated recognition and degradation of mRNAs lacking a termination codon. Science. 2002; 295:2262–2264. 11910110 10.1126/science.1067272

[108] Karamyshev A.L. , Karamysheva Z.N. Lost in translation: ribosome-associated mRNA and protein quality controls. Front. Genet. 2018; 9:431. 30337940 10.3389/fgene.2018.00431 PMC6180196

