# Supplementary material for: RBM6 splicing factor promotes homologous recombination repair of double-strand breaks and modulates sensitivity to chemotherapeutic drugs
Source: Nucleic Acids Res. 2021 Oct 28;49(20):11708–27. doi: 10.1093/nar/gkab976 (PMC8599755; doi:10.1093/nar/gkab976)
Supplement: gkab976_Supplemental_Files [file gkab976_supplemental_files.zip › Machour et al-Supplementary_Materials-NAR-00500-U-2021-R2.pdf]

Supplementary Materials for

RBM6 Splicing Factor Promotes Homologous Recombination Repair of Double-Strand Breaks and Modulates Sensitivity to Chemotherapeutic Drugs

Feras E. Machour, Enas R. Abu-Zhayia, Samah W. Awwad, Tirza Bidany-Mizrahi, Stefan Meinke, Laila A. Bishara, Florian Heyd, Rami I. Aqeilan and Nabieh Ayoub*

*Corresponding author. Email: ayoubn@technion.ac.il

This PDF file includes:

- Supplementary materials and methods
- Supplementary Figures and Legends: S1 to S21
- Key Resources Table
- Appendix 1

Supplementary materials and methods:

Cell lines

All cell lines were grown at 37°C and 5% CO2. All cell culture media were supplemented with 2mM L-glutamine (Gibco), and 100unit/mL penicillin and 100µg/mL streptomycin (Gibco). MDA-MB-231, HeLa, and HEK293T were cultured in Dulbecco's modified Eagle medium (Gibco) supplemented with 10 % heat-inactivated fetal bovine serum (Gibco). MCF7 cells were cultured in RPMI-1640 medium (Gibco) supplemented with 10 % heat-inactivated fetal bovine serum. MCF10A-Hras cells were cultured in DMEM/F12 media (Invitrogen) supplemented with 5% horse serum (Invitrogen), 20 ng/ml EGF (Peprotech), 0.5 mg/ml hydrocortisone (Sigma H-0888), 100 ng/ml cholera toxin (Sigma C-8052), and 10 µg/ml insulin (Sigma I-1882). The U2OS-HR-ind cell line (kind gift from Michal Goldberg, Hebrew University, Jerusalem, Israel) was cultured in a phenol red free-DMEM supplemented with charcoal-treated FCS, as previously described (1).

Generation of RBM6-knockout MCF10A cell lines using CRISPR/Cas9 methodology

Guide RNAs (gRNAs) were designed using Deskgen tool (deskgen.com). MCF10A-Hras cells were transfected with pSpCAS9(BB)-2A-GFP vector expressing GFP-Cas9 and gRNA targeting RBM6 or co-transfected with pSpCas9n(BB)-2A-GFP (PX461) vector expressing GFP-nickase-Cas9 and one RBM6 gRNA and pSpgRNA-RBM6 gRNA vector expressing another RBM6 gRNA. At 24 h after transfection, GFP-positive cells were sorted using BD LSRFortessa™ cell analyzer (BD Biosciences) and plated in 96-well plates at a dilution of one cell per well. Following single colony growth, clones were first screened by western blot analysis and positive RBM6-knockout clones were further validated by sequencing and immunofluorescence.

Generation of lentiviral particles and cell transduction

Gene expression knockdown using shRNA sequences was performed as previously described (2). First, scramble short hairpin oligonucleotides and short hairpin oligonucleotide directed against RBM6 or RNPS1 were annealed and inserted into pLKO.1-TRC lentiviral vector digested with *Eco*RI and *Age*I. The generated lentiviral vectors were verified by nucleotide sequencing. Viral particles containing the shRNA construct were generated by co-transfecting HEK293T cells plated in 10cm with the shRNA containing pLKO.1-TRC plasmid (1.64pmol), together with viral

packaging plasmids psPAX2 (1.3pmol) and pMD2.G (0.72pmol). Viral particles containing pLV-Flag-RBM6-T2A-Blast and pLV-myc-Fe65-Blast were generated as described above. Media containing the viral particles were collected 48 h post-transfection and filtered with 0.45 μm filters. Then, the viral particles were used to infect the indicated cell lines. At 72 h post-infection, cells were selected with the appropriate selection for 3-7 days (Blasticidin - 10μg/ml – 7 days; Puromycin - 1μg/ml – 3 days).

Western Blot

Protein extracts were prepared using Hot-lysis buffer (1% SDS, 5 mM EDTA, 50 mM Tris, pH 7.5) and protease inhibitor mixture (Calbiochem). Western blot analysis was then performed as previously described (3). A complete list of antibodies and their dilutions are described in the Key Resources table.

RNA Isolation, reverse transcription, RT-PCR and qPCR

Total RNA was isolated from cells using the TRIzol reagent according to the manufacturer's instructions (Ambion) and treated with RQ1 DNase (M6101, Promega). 1 μg RNA was used for cDNA synthesis using the qScript cDNA Synthesis Kit (Quanta) with random primers. RT-PCR was performed using standard PCR using Red Load Taq Master (Jena Bioscience). mRNA levels were measured by real-time PCR in the Step-One-Plus real-time PCR System (Applied Biosystems) using the indicated primers and the Fast SYBR Green Master mix (Applied Biosystems) with three technical repeats for each PCR. Data analysis and quantification were performed using StepOne software V2.2 supplied by Applied Biosystems. As shown in Appendix 1 in the supplementary methods, standard curves were performed to determine the efficiency and linear range of the qPCR reactions described in Figures 3H ,3J, 3L, 4B-D, 4F-G, 5H. GAPDH gene was used as a housekeeping gene.

Clonogenic survival assay

For colony survival assay, cells seeded onto 6-well plates (500 cells per well), and treated with the indicated doses of IR or PARPi as specified in the figure legends. After 14 days, cells were fixed methanol and stained with 0.25% crystal violet. Individual colonies were counted manually and the percentage survival was calculated by normalizing to the number of colonies in untreated wells.

Short-term growth delay assay

For determining IR-sensitivity, cells were exposed to the indicated IR dosage in triplicates and then plated in 96-well plates at a density of 5000 cells per well. For determining drug sensitivity, cells were seeded in 96-well plates in triplicates at a density of 2000-5000 cells per well. 24 hours post-seeding, PARPi, ATMi, or cisplatin were added at the indicated concentrations. Cell viability was measured 48-96 hrs after drug treatment using the CellTiter 96® AQueous One Solution Cell Proliferation Assay (Promega) following the manufacturer's protocol, and absorbance was measured using Epoch Microplate Spectrophotometer (BioTek). Cell viability was normalized to the viability of untreated cells.

APEX2-tagging of the endogenous RBM6 protein

CRISPR-Cas9 was used to fuse 3xFLAG-APEX2 at the N terminus of endogenous RBM6. MCF10A cells were co-transfected with px330 plasmid containing Cas9 nuclease and gRNA targeting the start of the RBM6 coding sequence and a donor plasmid containing puromycin resistance selection gene, P2A self-cleavage site, and 3x-FLAG-APEX2 flanked by homology arms (~700bp each side) corresponding to RBM6 gene. Puromycin resistant cell clones were screened by genomic PCR using the primer sequences provided in the key resources table. Positive clones were further validated by western blot and by monitoring APEX2-dependent protein biotinylation in the engineered cell lines.

APEX-based proximity labeling and affinity enrichment of biotinylated proteins and preparation for MS analysis

Control and 3xFLAG-APEX2-RBM6-expressing cells were incubated with 0.5mM biotin-phenol reagent for 4hrs prior to start of the labeling reaction. The cells were washed with PBS and the labeling reaction was initiated by adding 1mM H2O2 in PBS for 2 min at room temperature. The reaction was terminated by washing cells thrice with a quencher solution containing 10mM sodium azide, 10mM sodium ascorbate, and 5mM Trolox in PBS. Subsequently, the cells were washed twice with ice-cold PBS and cytoplasmic and nuclear fractions were prepared as follows: cell pellet was resuspended with ~3 volume with ice-cold Buffer A (10 mM HEPES (pH=7.9), 10 mM KCl, 1.5 mM MgCl2, 0.34 M sucrose, 10% Glycerol) supplemented with 0.2% Triton and protease inhibitor mixture (Calbiochem) and incubated 15 minutes on ice. Next, Nuclei were collected by centrifugation at 2,000 g for 5 min at 4oC. The nuclear pellet was lysed with RIPA buffer (50mM Tris (pH 7.5), 1M sodium chloride, 1% NP-40, 0.1% sodium deoxycholate, 1mM EDTA)

supplemented with complete protease inhibitor cocktail (Calbiochem), 1mM DTT, 5mM Trolox, 10 mM Sodium Azide, and 10 mM Sodium Ascorbate. Nuclear lysate was incubated on ice for 10 min, sonicated at amplitude 35 for 1 min, Samples were treated with benzonase for 20 min at room temperature before clarifying the lysate by centrifugation at 12,800 g for 15 min. Clarified lysate was collected and proteins were quantified by Bradford's reagent. 40µg of nuclear lysate was resolved on SDS-PAGE gel and blotted with Streptavidin-HRP to validate APEX2 activation and functionality.

Streptavidin magnetic beads (GenScript) were dimethylated by 2hrs incubation with 0.04M Formaldehyde and 0.02M Cyanoborohydrid at room temperature and pH=7, followed by 8hrs incubation at 4°C and pH=8. Residual demethylation reagents were depleted by washing the beads with RIPA buffer before the addition of the nuclear lysate. 2.5mg nuclear lysates from control and 3xFlag-APEX2-RBM6 expressing cells were incubated with 15µl di-methylated Streptavidin magnetic beads (Cat# L00424) at 4°C with rotation overnight. Beads were then washed twice with RIPA buffer containing 200mM NaCl, followed by 5 washes with 0.1M Ammonium Bicarbonate (ABC). Next, beads were incubated with ABC supplemented with 10mM DTT at 65oC for 30 minutes followed by the addition of 25mM Chloroacetamide and incubation for 30 minutes at room temperature. Next, Trypsin was added to a final concentration of 200 ng/ µl and incubated overnight at 37oC for on-bead digestion. Digestion was quenched by the addition of 0.1% Trifluoroacetic acid (TFA) and supernatant was collected followed by additional extraction step with 0.1% TFA, 5% Acetonitrile, 0.1% ABC. Eluates were dried completely using SpeedVac and desalted on reversed-phase C18 StageTips before sending for Mass spectrometry analysis. Finally, peptides were resuspended in a buffer containing 2% acetonitrile and 0.1% Formic Acid before injecting into mass spectrometry (MS) at the Smoler Proteomics Center in the department of Biology in the Technion.

MS analyses

The samples were analyzed by LC-MS/MS using Q-Exactive plus mass spectrometer (Thermo Scientific), coupled to Easy nano LC-1000 capillary UHPLC (Thermo Scientific). The resulted tryptic peptides from on-beads digestions were resolved by a reverse phase chromatography using homemade fused silica capillary (0.075x200mm) packed with Reprosil reversed phase material (Dr Maisch GmbH), in 0.1% formic acid. The peptides were eluted with a 120 min linear gradient of 5% to 28% acetonitrile with 0.1% formic acid (in water), followed by 15 min linear gradient

28% to 95%, and 10 min at 95% acetonitrile with 0.1% formic acid at flow rates of 0.15 μl/min. Mass-spectrometry was performed with data dependent acquisition mode for positive ions at mass range of 300-1800 m/z with resolution of 7000, selecting the 10 most intense ions (with charge>1) in each full MS. Ions fragmentation in MS/MS was done by collision-induced dissociation (CID) at 35 normalized collision energy. The AGC was set to 3 × 106 for the full MS and to 1 × 105 for the MS/MS scans. The intensity threshold for triggering MS/MS was set 1 × 104, and the dynamic exclusion duration was set to 20 sec. The MS data was clustered and analyzed using the Perseus software platform and maxquant (4) searching against the human section of the Uniprot database. Proteins enriched in APEX2-RBM6-expressing cells were subsequently analyzed for gene set enrichment of biological themes using DAVID bioinformatics platform (5).

Animals

Animal studies and protocols were approved by the Committee on the Ethics of Animal Experiments of the Technion, Israel Institute of Technology (IL-116-07-2019). Immunocompromised NOD.CB17-Prkdc^{scid}/NcrCrl (NOD SCID) mice were purchased from Envigo (170). Mouse model of MDA-MB-231 subcutaneous xenografts were established by transplanting cells into 5-week-old female NOD SCID mice. Mice were randomly assigned into control and treatment groups. Prior to initiating any experiments, mice were allowed one week acclimation to housing conditions at the Technion Animal Facility. All mice were housed in a strict pathogen-free environment.

Breast tumor microarrays

Human Paraffin-embedded Tissue arrays of breast cancer patients were purchased from US Biomax, Inc.: BR1005b, BR1008b, and BR2082c. Paraffin embedded tumor arrays were deparaffinized and rehydrated. Antigen retrieval was performed in 25mM sodium citrate buffer PH 6.0 using pressurized chamber for 2.5 min. Endogenous peroxidase was blocked with 3% H2O2 for 15 min. The sections were then incubated with blocking solution (CAS Block) for 30 min to reduce non-specific binding followed by incubation with the RBM6 antibody (abcam) overnight. Slides were subsequently incubated with horseradish peroxidase-conjugated anti-rabbit immunoglobulin antibody for 30 min. The enzymatic reaction was detected in a freshly prepared 3,3 diamminobenzidine using DAB peroxidase kit for several min at room temperature. The sections were then counterstained with hematoxylin.

Supplementary References

1. Shahar, O.D., Raghu Ram, E.V., Shimshoni, E., Hareli, S., Meshorer, E. and Goldberg, M. (2011) Live imaging of induced and controlled DNA double-strand break formation reveals extremely low repair by homologous recombination in human cells. *Oncogene*, **31**, 3495-3504.
2. Awwad, S.W., Abu-Zhayia, E.R., Guttmann-Raviv, N. and Ayoub, N. (2017) NELF-E is recruited to DNA double-strand break sites to promote transcriptional repression and repair. *EMBO Rep*, **18**, 745-764.
3. Abu-Zhayia, E.R., Awwad, S.W., Ben-Oz, B.M., Khoury-Haddad, H. and Ayoub, N. (2018) CDYL1 fosters double-strand break-induced transcription silencing and promotes homology-directed repair. *J Mol Cell Biol*, **10**, 341-357.
4. Tyanova, S., Temu, T. and Cox, J. (2016) The MaxQuant computational platform for mass spectrometry-based shotgun proteomics. *Nat Protoc*, **11**, 2301-2319.
5. Huang da, W., Sherman, B.T. and Lempicki, R.A. (2009) Systematic and integrative analysis of large gene lists using DAVID bioinformatics resources. *Nat Protoc*, **4**, 44-57.
6. Ghandi, M., Huang, F.W., Jane-Valbuena, J., Kryukov, G.V., Lo, C.C., McDonald, E.R., 3rd, Barretina, J., Gelfand, E.T., Bielski, C.M., Li, H. *et al.* (2019) Next-generation characterization of the Cancer Cell Line Encyclopedia. *Nature*, **569**, 503-508.
7. Cancer Genome Atlas Research, N., Weinstein, J.N., Collisson, E.A., Mills, G.B., Shaw, K.R., Ozenberger, B.A., Ellrott, K., Shmulevich, I., Sander, C. and Stuart, J.M. (2013) The Cancer Genome Atlas Pan-Cancer analysis project. *Nat Genet*, **45**, 1113-1120.
8. Gao, J., Aksoy, B.A., Dogrusoz, U., Dresdner, G., Gross, B., Sumer, S.O., Sun, Y., Jacobsen, A., Sinha, R., Larsson, E. *et al.* (2013) Integrative analysis of complex cancer genomics and clinical profiles using the cBioPortal. *Science signaling*, **6**, pl1.
9. Cerami, E., Gao, J., Dogrusoz, U., Gross, B.E., Sumer, S.O., Aksoy, B.A., Jacobsen, A., Byrne, C.J., Heuer, M.L., Larsson, E. *et al.* (2012) The cBio cancer genomics portal: an open platform for exploring multidimensional cancer genomics data. *Cancer discovery*, **2**, 401-404.
10. Li, W., Tam, K.M.V., Chan, W.W.R., Koon, A.C., Ngo, J.C.K., Chan, H.Y.E. and Lau, K.F. (2018) Neuronal adaptor FE65 stimulates Rac1-mediated neurite outgrowth by recruiting and activating ELMO1. *J Biol Chem*, **293**, 7674-7688.
11. Pinder, J., Salsman, J. and Dellaire, G. (2015) Nuclear domain 'knock-in' screen for the evaluation and identification of small molecule enhancers of CRISPR-based genome editing. *Nucleic Acids Res*, **43**, 9379-9392.

Supplementary Figures S1-S21:

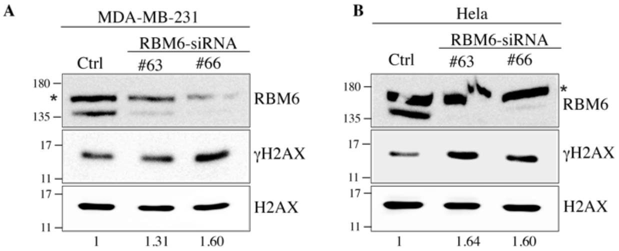

Supplementary Figure 1

(**A-B**) RBM6 depletion increases γH2AX levels. MDA231 (A) and Hela (B) cells were transfected with siRNA against RBM6 and subjected to western blot analysis with the indicated antibodies. H2AX was used as loading control. The bands intensities of γH2AX were normalized relative to the intensities of their respective H2AX bands and the ratios are shown at the bottom of the blot. * indicates unspecific band.

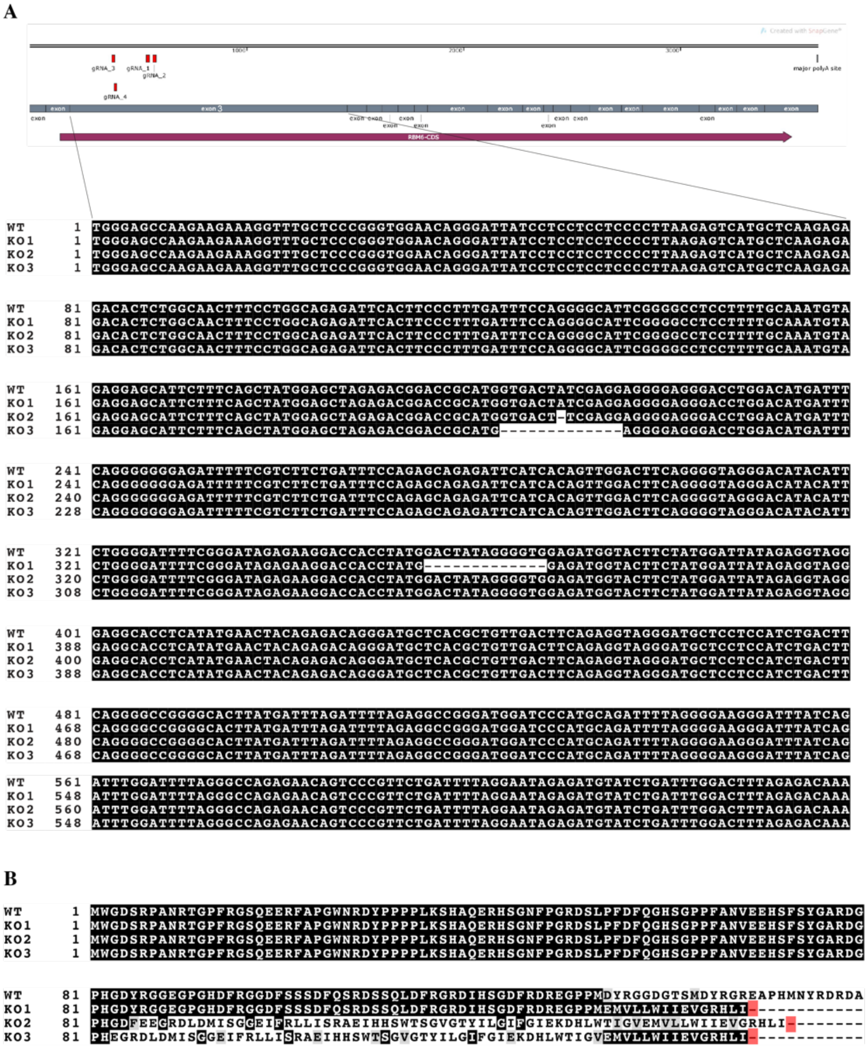

Supplementary Figure 2

(**A**) Top: schematic of RBM6 exon structure. Indicated are the positions of gRNAs targeting RBM6 exon 3 used to obtain the 3 different RBM6 knockout clones. gRNA1 and gRNA2 were used to obtain knockout clone #1 (KO1) using Cas9-D10A nickase. gRNA3 and gRNA4 were used to obtain knockout clones #2 and #3 (KO2 and KO3), respectively. Bottom: Sequence alignment of RBM6 exon 3 showing the deletions obtained in RBM6 KO1-3 as determined by Sanger sequencing. (**B**) Shows protein sequence alignment between RBM6-WT and the 3 RBM6-KO clones. Red boxes highlight the position of the pre-mature stop codon following CRISPR-Cas9 frame-shift mutations.

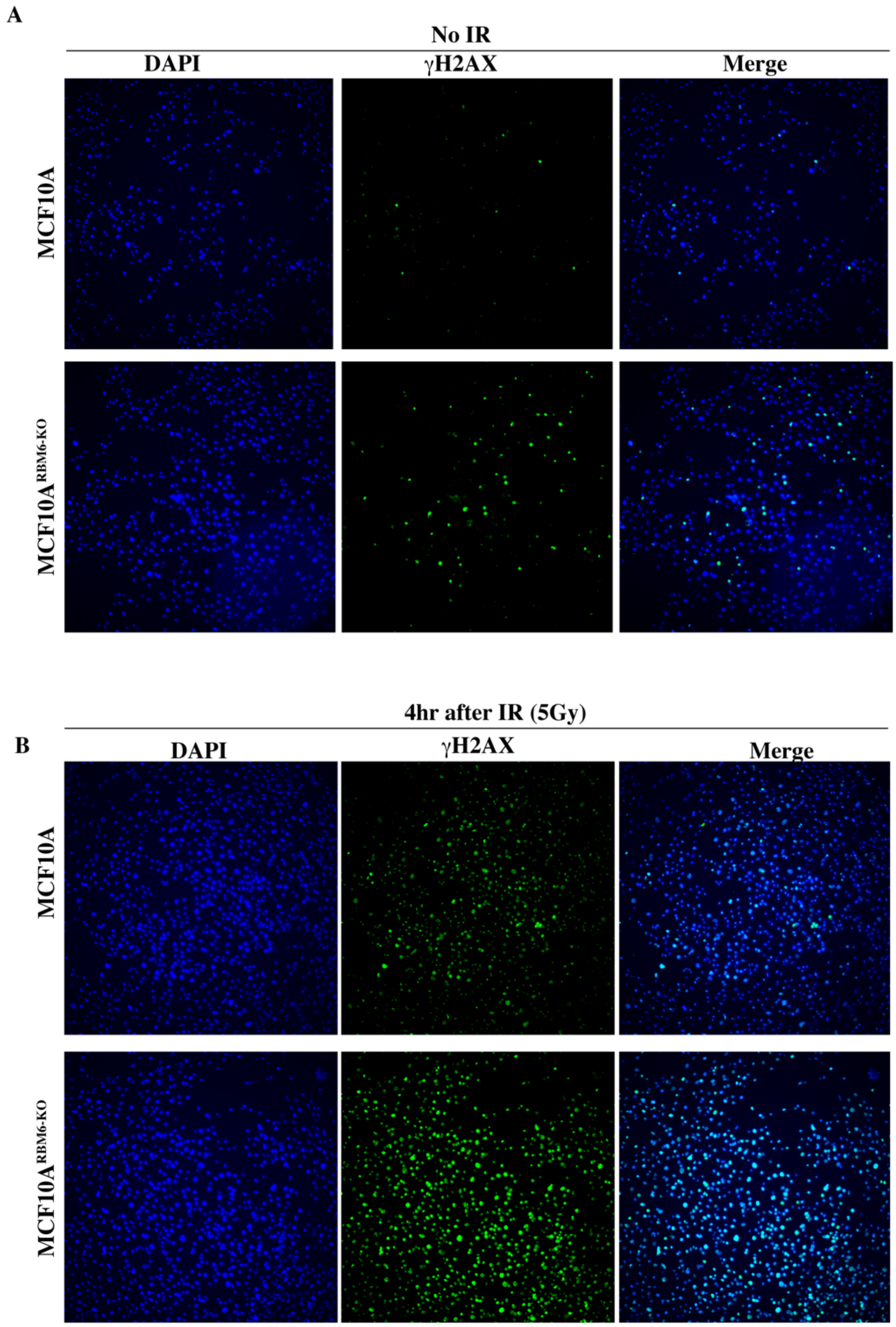

Supplementary Figure 3

(**A-B**) Representative images related to Figure 1E. Control and MCF10A RBM6-KO1 cells were fixed and stained for γH2AX and DAPI before (A) and after 4hrs exposure to IR (5Gy) (B). Images were obtained using high-content screening microscope (IN Cell Analyzer 2000; GE Healthcare).

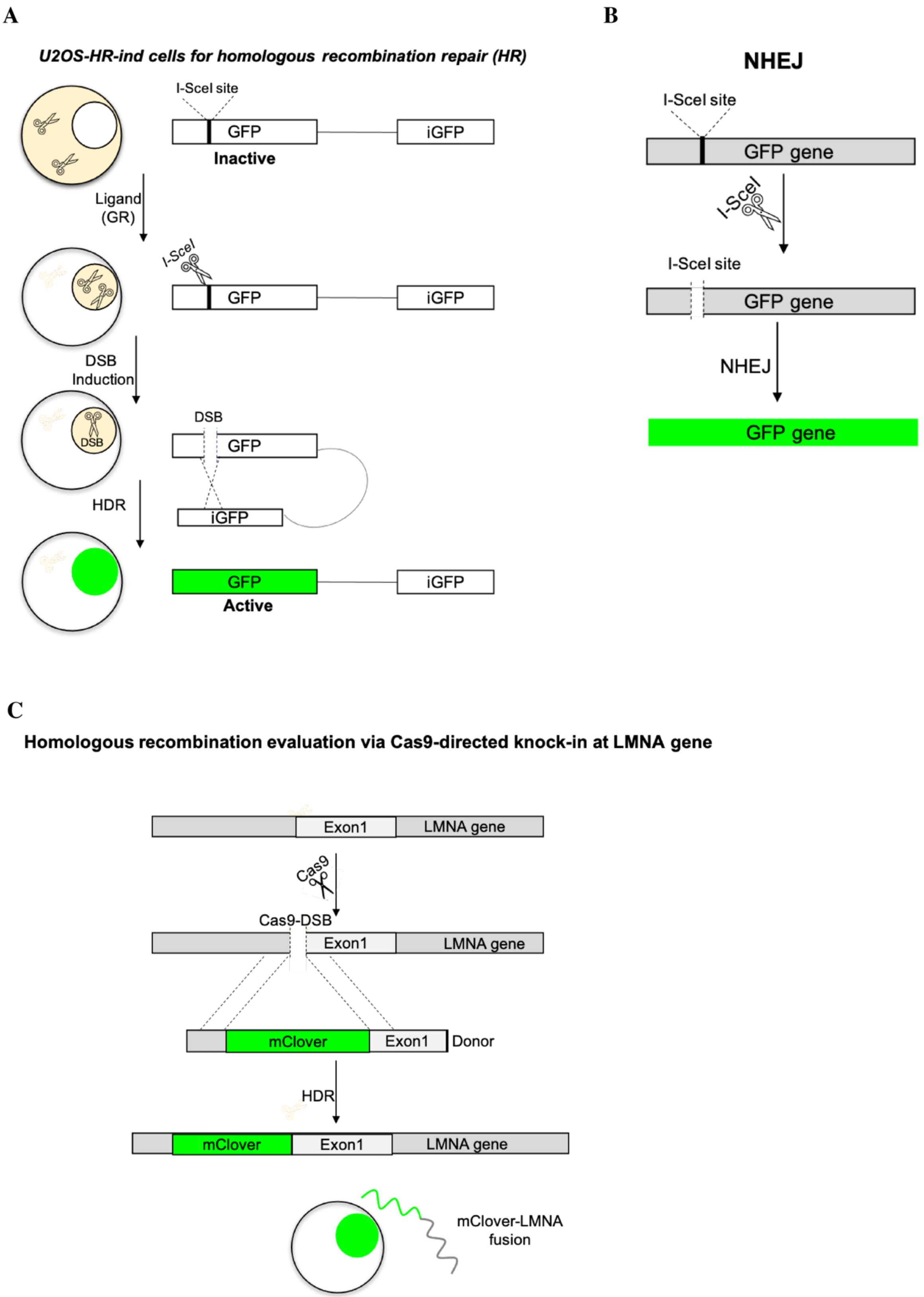
A
U2OS-HR-ind cells for homologous recombination repair (HR)
I-SceI site
GFP
iGFP
Inactive
Ligand
(GR)
I-SceI
GFP
iGFP
DSB
Induction
DSB
DSB
GFP
iGFP
HDR
GFP
iGFP
Active
B
NHEJ
I-SceI site
GFP gene
I-SceI
I-SceI site
GFP gene
NHEJ
GFP gene
C
Homologous recombination evaluation via Cas9-directed knock-in at LMNA gene
Exon1
LMNA gene
Cas9
Cas9-DSB
Exon1
LMNA gene
mClover
Exon1
Donor
HDR
mClover
Exon1
LMNA gene
mClover-LMNA
fusion

Supplementary Figure 4

Schematic representation of HR and NHEJ reporter assays. (**A**) U2OS-HR-ind cell line continuously expresses cytoplasmatic I-SceI endonuclease fused to Estrogen Receptor sequence and contains an I-SceI recognition site integrated within a GFP-reporter cassette together with inverted GFP sequence (iGFP). Following the addition of Doxycycline, I-sceI endonulclease migrates to the nucleus and generates DSB within its recognition site. During homologous recombination, the cells use the iGFP sequence as a template to repair DSB and thus GFP gene will be activated. (**B**) Hela-PEJJSA cell line containing I-SceI recognition site integrated within a GFP-reporter cassette. Following I-SceI transfection, DSBs are generated within the GFP gene sequence, and DSB repair by NHEJ restores GFP integrity. (**C**) Cas9 endonuclease targeted to the first exon of the LMNA gene is transfected to the cells together with a donor plasmid. Cas9-directed knock-in of mClover in the LMNA coding sequence indicates HR efficiency.

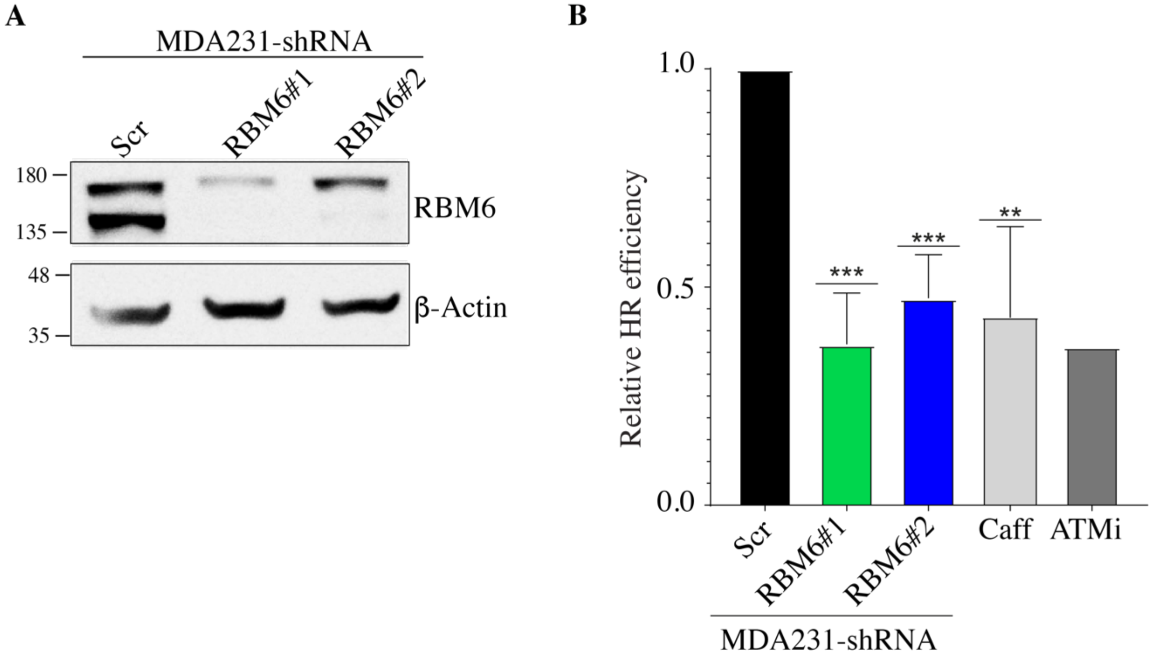

Supplementary Figure 5

(**A**) Western blot analysis shows expression of RBM6 in MDA231 cells expressing RBM6 shRNAs. (**B**) HR efficiency in MDA231 cells depleted of RBM6 was measured as in Fig. 2C. Caffeine and ATM inhibition were used as positive controls.

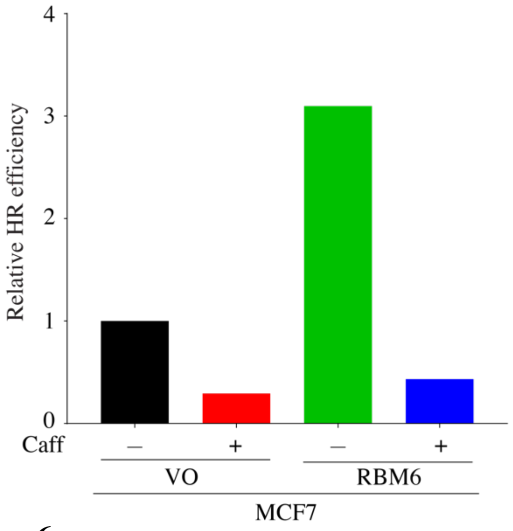

Supplementary Figure 6
HR efficiency in MCF7 cells complemented with empty vector or vector expressing RBM6[WT] fused to Mono-Red (MR) with or without Caffeine treatment. HR efficiency of endogenous DSBs at the LMNA gene was measured as described in Figure 2C.

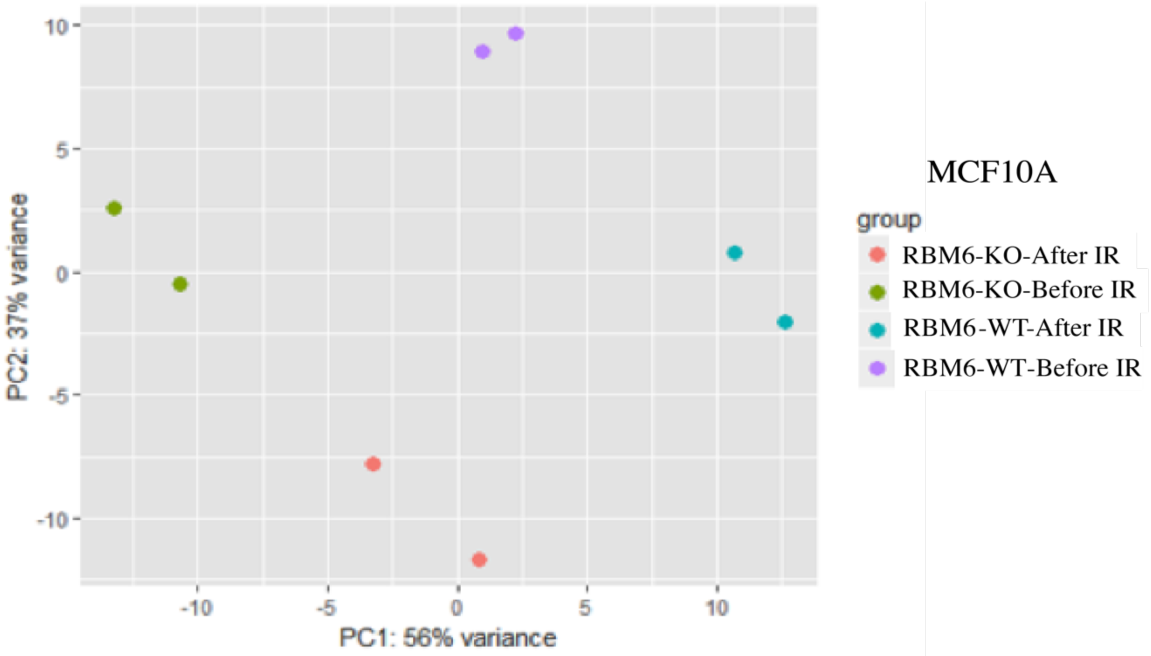

Supplementary Figure 7
Principal component analysis (PCA) of RNA-seq data of control and RBM6-KO1 MCF10A cells before and after 12hrs exposure to IR (5Gy) shows a high degree of reproducibility among the replicate samples within each group and significant variance between the different groups. (Related to Figure 3)

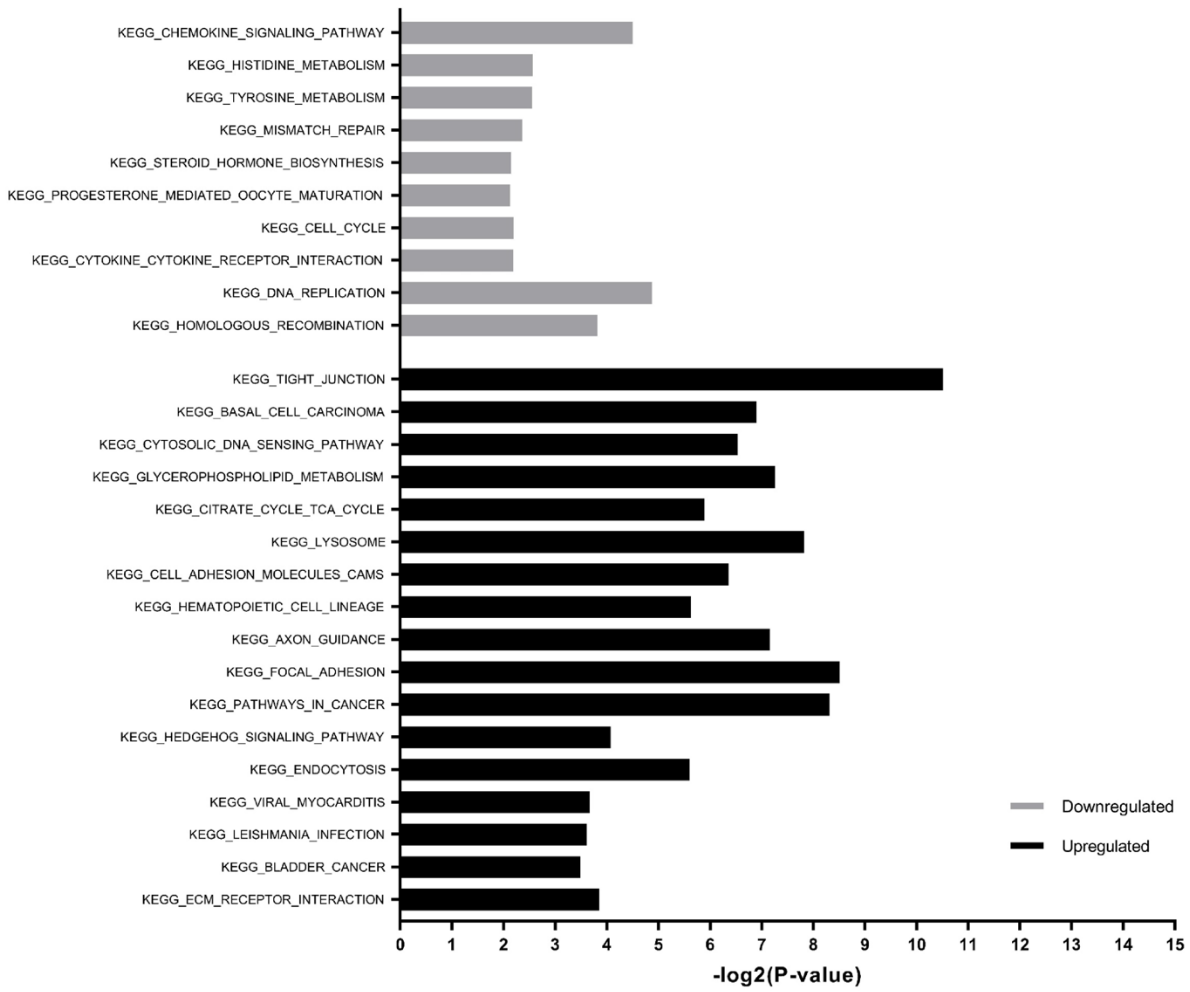

Supplementary Figure 8
Gene set enrichment analysis (KEGG pathways) of differentially expressed genes obtained from RNA-seq data between control and RBM6-KO1 MCF10A. (Related to Figure 3)

Supplementary Figure 9
Western blot analysis shows that complementing MCF10A RBM6-KO1 cells with Flag-RBM6 led to an increase in Fe65 protein level. α-Tubulin was used as a loading marker. Band intensities of Fe65 from 3 independent experiments were normalized to the intensities of their respective α-Tubulin bands and the mean normalized ratio ± SD is shown at the bottom of the blot. Two-tailed t-test: P-value=0.0014.

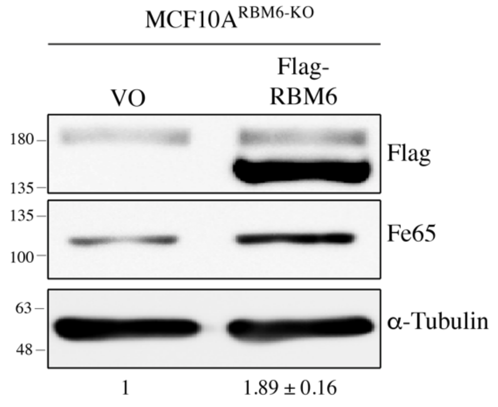

A

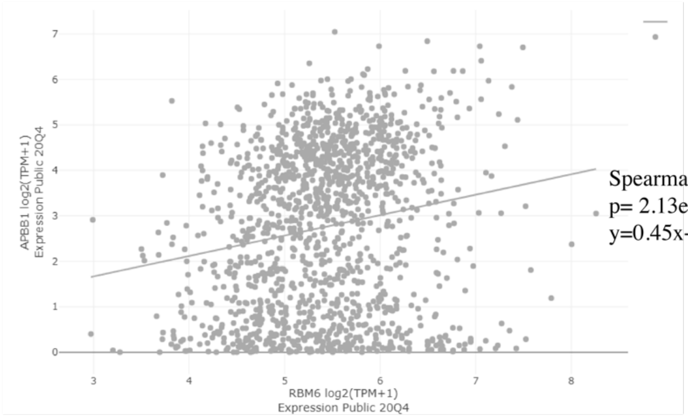

Spearman: 0.186
p= 2.13e-11
y=0.45x+0.32

B

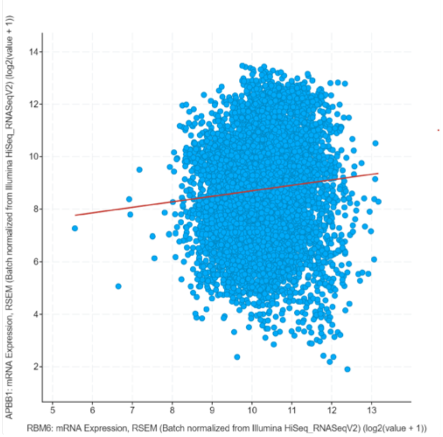

Spearman: 0.12
(p = 9.96e-36)
Pearson: 0.09
(p = 9.94e-19)
y = 0.21x + 6.59
R^2 = 0.01

C

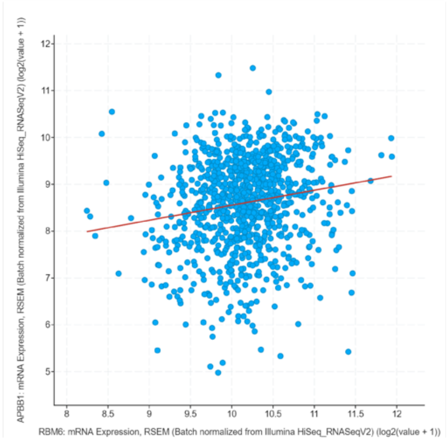

Spearman: 0.17
(p = 4.05e-8)
Pearson: 0.16
(p = 2.77e-7)
y = 0.32x + 5.35
R^2 = 0.03

Supplementary Figure 10

Correlation between RBM6 and Fe65 (APBB1) mRNA expression across human cancer cell lines (A), pan-cancer patients (B) and breast cancer patients (C). Cancer cell line expression data was obtained from the CCLE database (6) and correlation analysis was performed using DepMap platform (doi:10.6084/m9.figshare.13237076.v1). RNA-seq data from cancer patients was obtained from the TCGA database (7) and correlation analysis was performed using cBioPortal platform (8,9). (Related to Figure 3).

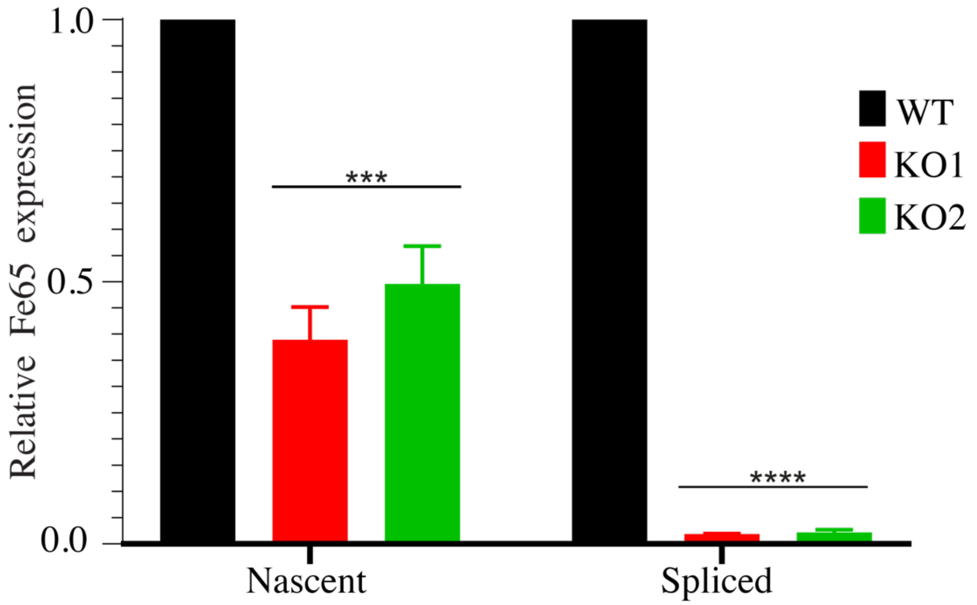

Supplementary Figure 11

RT-qPCR analysis using primers to detect nascent (unspliced) and spliced Fe65 transcript. Shown is the relative expression of spliced and unspliced Fe65 transcript in control and MCF10A RBM6-KO cells. Gene expression was normalized to the levels of GAPDH transcript. Data are presented as mean ± SD of three independent experiments. *$p<0.01$, **$p < 0.001$, ***$p < 0.0001$, ****$p < 0.00001$. Related to Figure 3L.

A

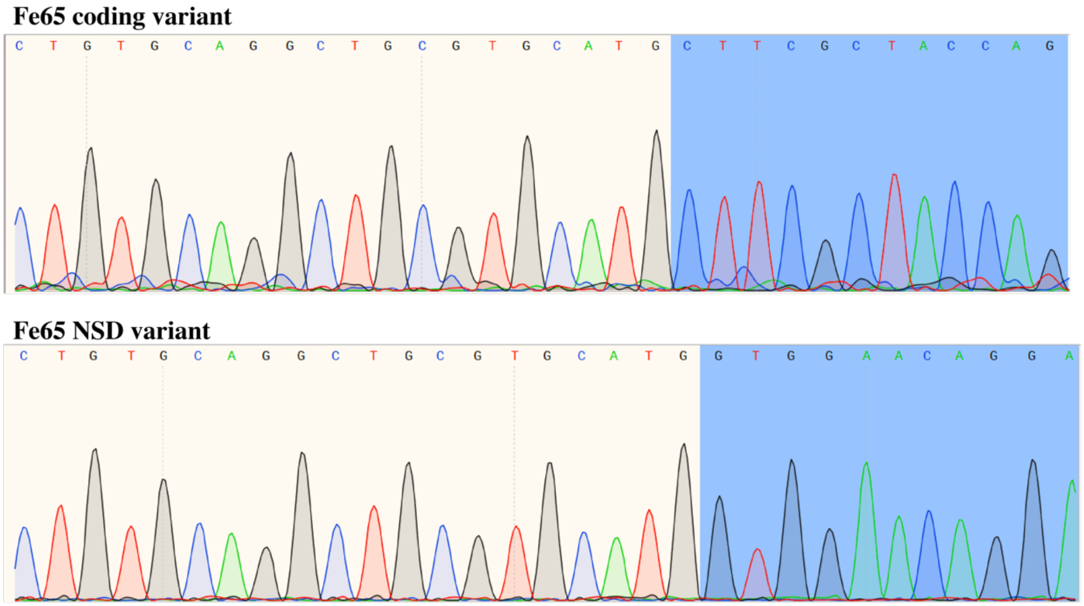

B

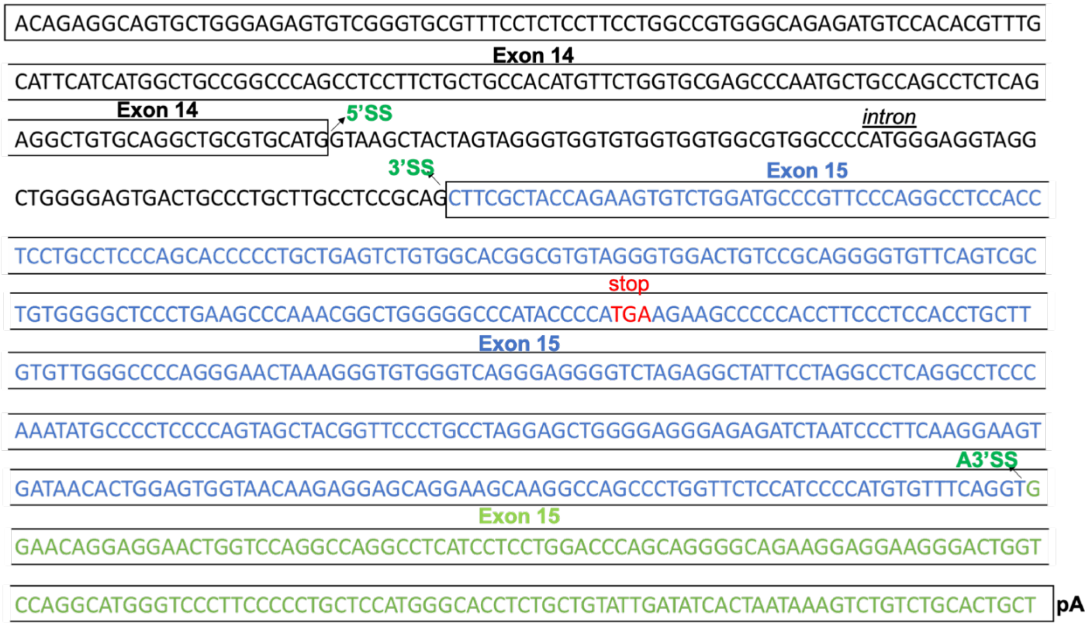

Supplementary Figure 12

(**A**) Sequencing of Fe65 variants in MCF10A. Fe65 coding and NSD variants were amplified by RT-PCR using variant specific primers and cloned into T-vector and subjected to Sanger sequencing. Highlighted in blue is the alternatively spliced exon 15 of Fe65. (**B**) DNA sequence corresponding to the 3' genomic sequence of Fe65 encompassing exons 14 and 15. The proposed splice sites resulting in either the correct (coding) Fe65 transcript and the NSD transcript lacking the stop codon are indicated within the sequence.

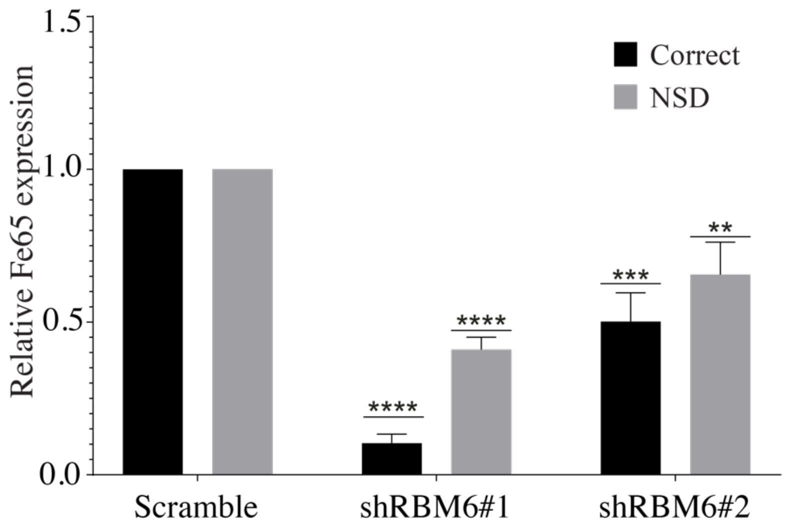

Supplementary Figure 13
RT-qPCR analysis using primers to detect correct (coding) and NSD Fe65 transcript. Shown is the relative expression of correct and NSD Fe65 transcript in MCF10A cells expressing either scramble shRNA or shRNAs against RBM6. Fe65 transcript levels were normalized to the levels of GAPDH transcript. Data are presented as mean ± SD of three independent experiments. *p<0.01, **p < 0.001, ***p < 0.0001, ****p < 0.00001. Related to Figure 4F.

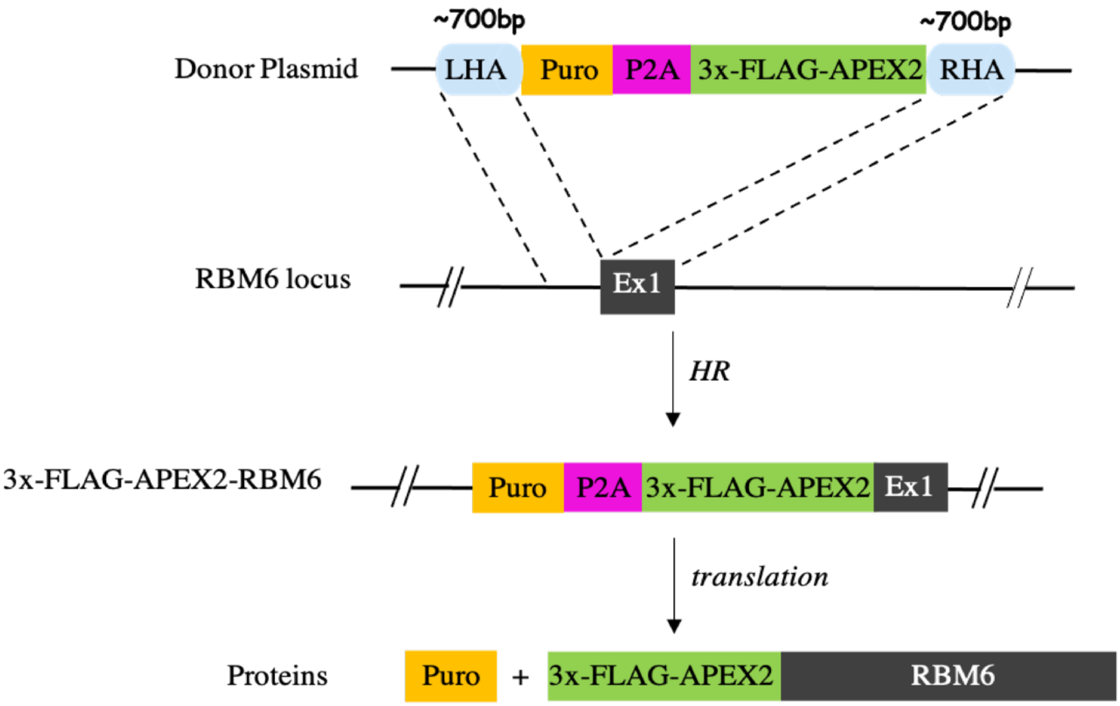

Supplementary Figure 14
Schematic diagram describing the strategy for CRISPR-based FLAG-APEX2 knock-in at the N-terminal of RBM6 protein (Related to Figure 5E).

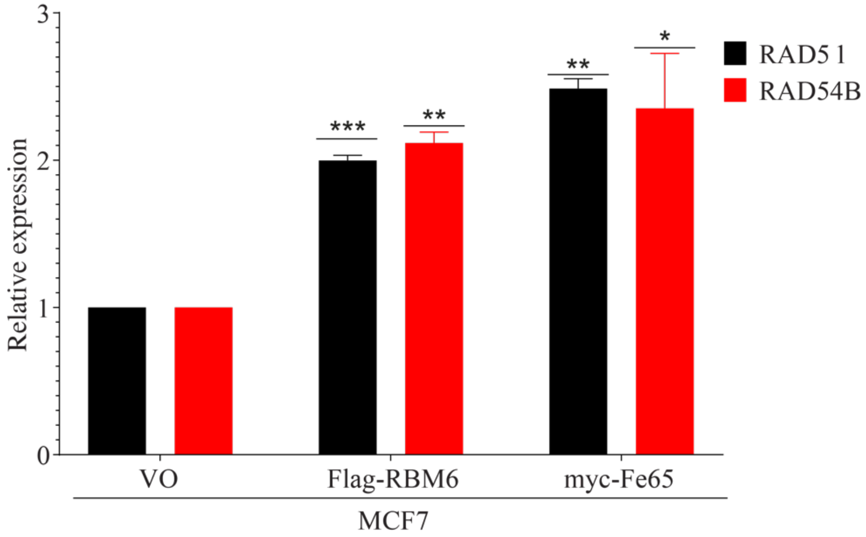

Supplementary Figure 15
MCF7 cells were complemented with empty vector or vector expressing either Flag-RBM6 or Myc-Fe65 and subjected to RT-qPCR analysis to measure the relative expression of RAD51 and RAD54B. Gene expression was normalized to the levels of GAPDH transcript. Data are presented as mean ± SD of three independent experiments. *p<0.01, **p < 0.001, ***p < 0.0001.

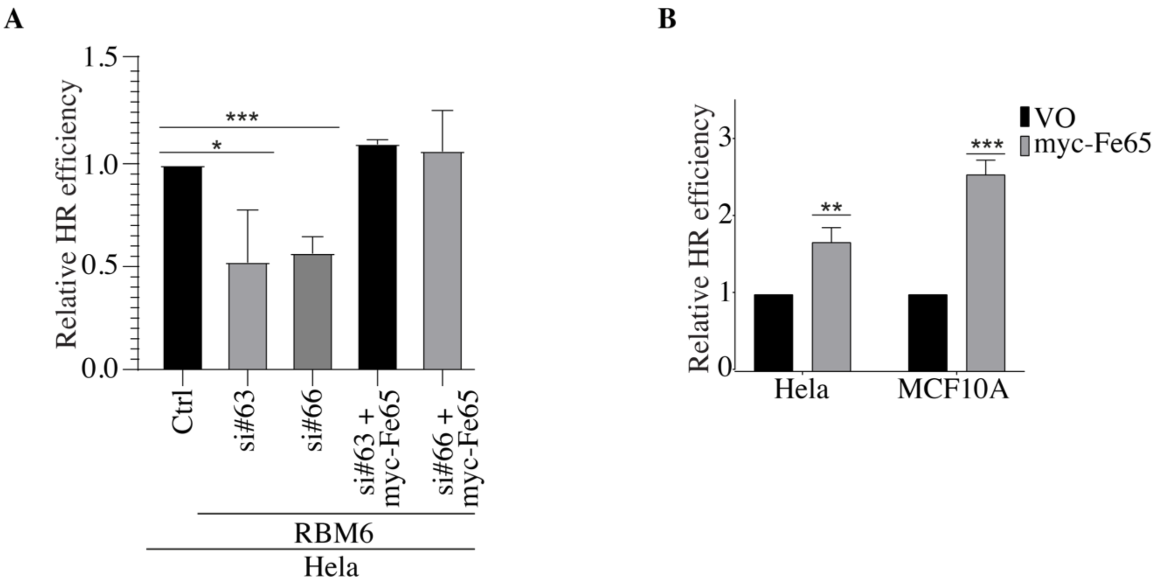

Supplementary Figure 16
(**A**) Hela cells depleted of RBM6 using two different siRNAs were complemented with vector expressing myc-Fe65 fused to Mono-Red (MR) and HR efficiency of endogenous DSBs at the LMNA gene was measured as described in Figure 2B. (**B**) As in (A), except that control HeLa and MCF10A cells were transfected with empty vector or vector expressing myc-Fe65 fused to Mono-Red (MR).

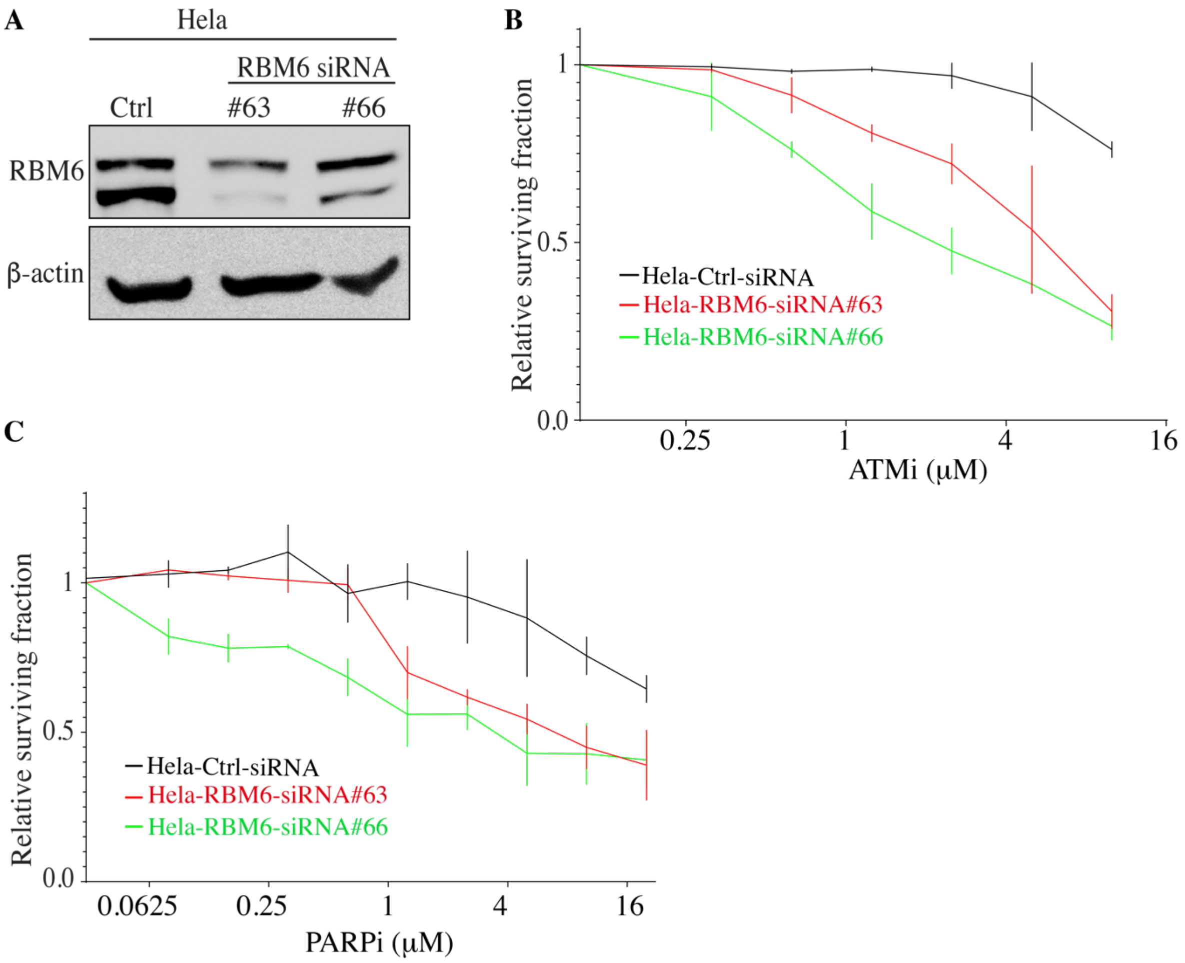

Supplementary Figure 17

(**A**) Western blot analysis shows the expression of RBM6 in Hela cells transfected with siRNAs against RBM6. (**B-C**) Hela cells deficient of RBM6 show hypersensitivity to ATM inhibition (B) and PARP inhibition (C). Cells were treated with increasing doses of ATMi or PARPi and cell viability was assessed by the CellTiter-Glo assay. Data are presented as mean ± SD of three independent experiments.

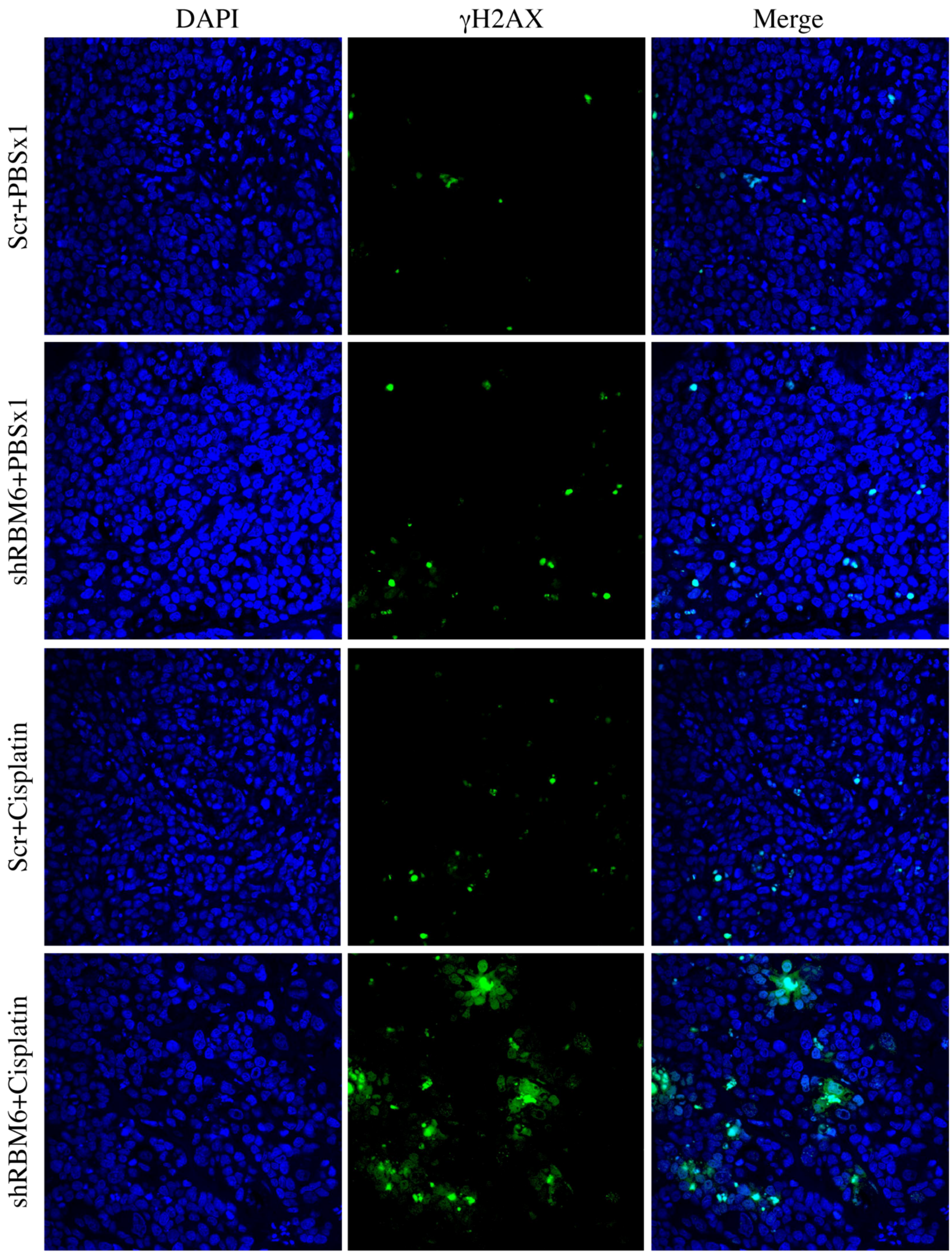

Supplementary Figure 18

Representative images of γH2AX staining in control (Scramble) and RBM6-deficient (RBM6-sh#1) MDA231 xenografts. Related to Figure 8E.

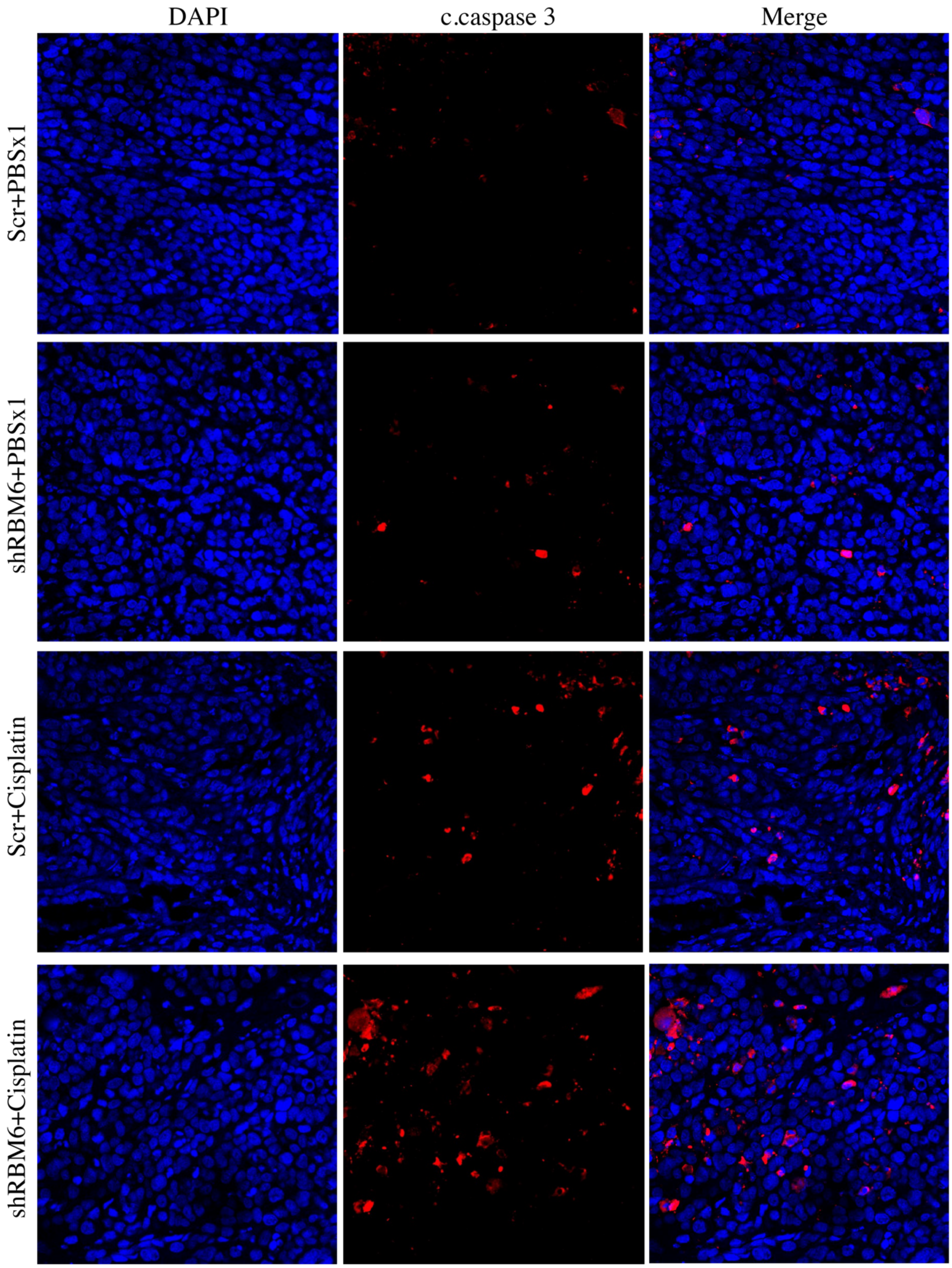

Supplementary Figure 19

Representative images of cleaved caspase 3 (c.caspase) staining in control (Scramble) and RBM6-deficient (RBM6-sh#1) MDA231 xenografts. Related to Figure 8F.

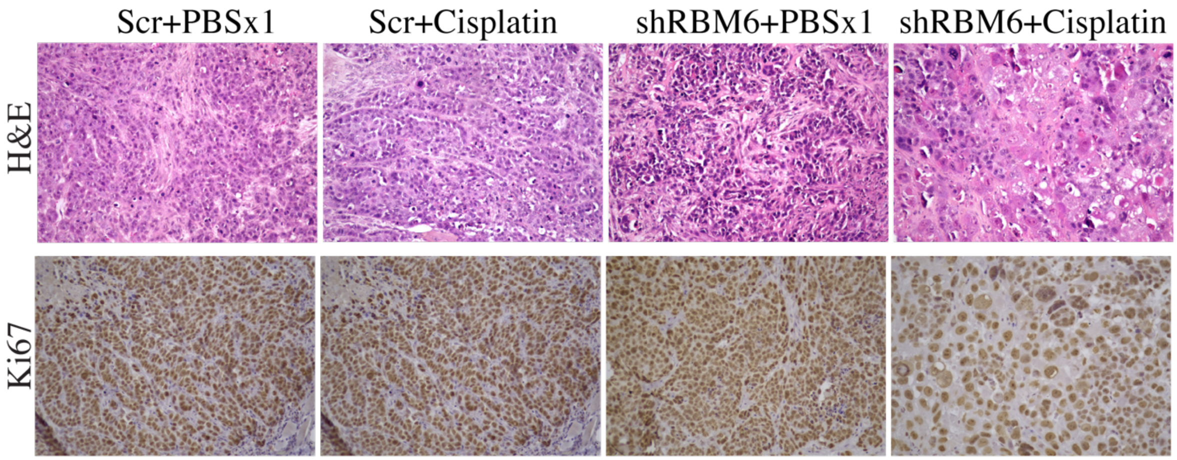

Supplementary Figure 20
Representative immunohistochemistry (IHC) micrographs showing H&E staining and Ki67 expression in MDA231 xenografts from. Related to Figure 8C.

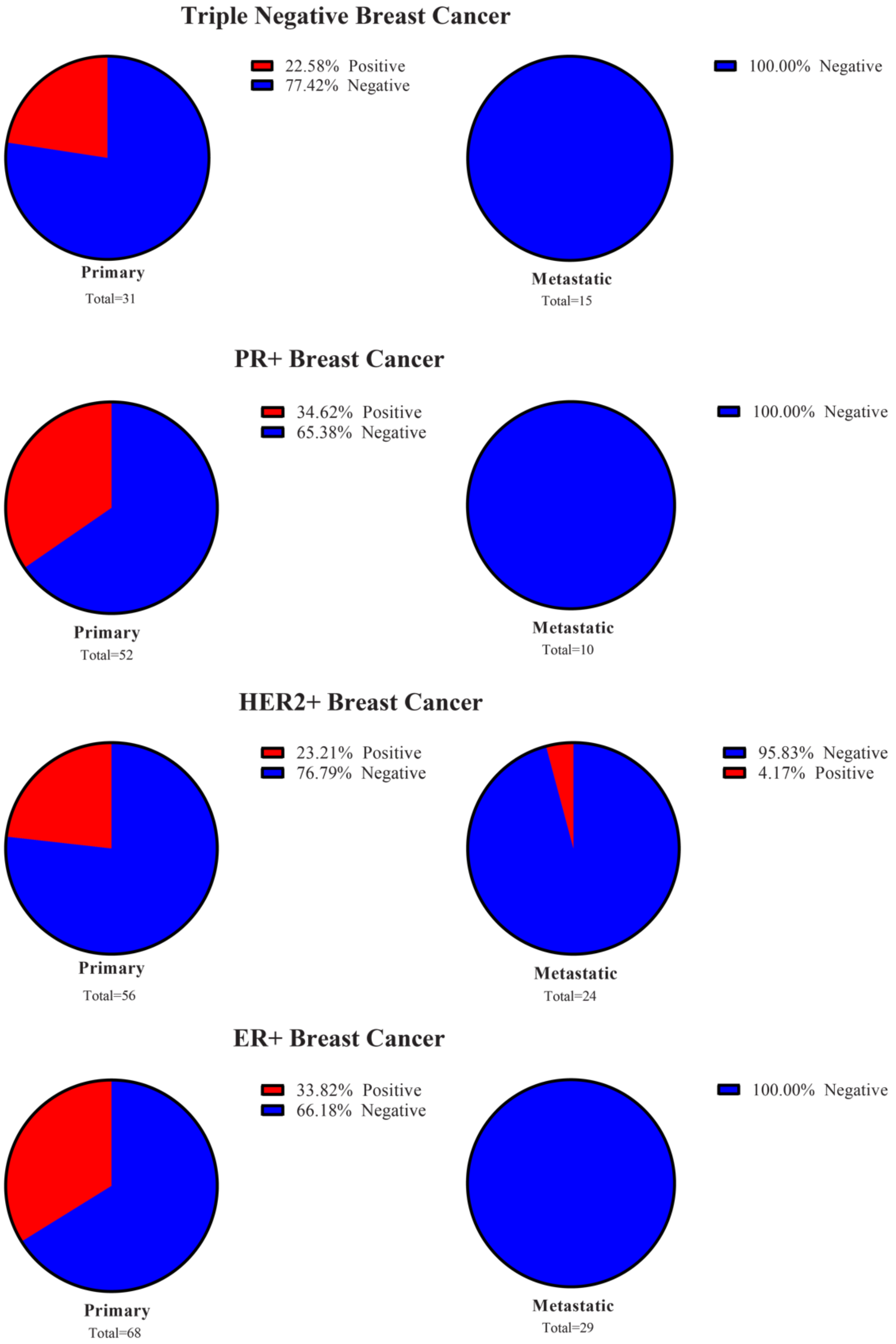

Supplementary Figure 21
IHC analysis of RBM6 protein expression in primary and metastatic breast cancers of different subtypes (US Biomax – BR1008b and BR2082c). Related to Figure 8I.

Key Resources Table

Reagent or Resource	Source	Identifier
**Antibodies**		
Rabbit monoclonal anti-RBM6 (WB 1:4000; IHC 1:1000	Abcam	ab188318
Rabbit polyclonal anti-Histone H2A.X phospho Ser-139 (γH2AX) (WB 1:1000)	Cell Signaling Technology	2577
Rabbit monoclonal anti-Histone H2A.X phospho Ser-139 (γH2AX) (IF 1:200)	Cell Signaling Technology	9718
Mouse monoclonal anti-phospho-Histone H2A.X (Ser139 clone JBW301 (IF 1:2500)	Millipore	05-636
Rabbit monoclonal anti-Histone H2A.X [EPR895] (WB 1:1000)	Abcam	ab124781
Rabbit polyclonal anti-FLAG (WB 1:5000)	Sigma-Aldrich	F7425
Mouse monoclonal anti-α-Tubulin (WB 1:1000)	Santa Cruz Bio	sc-5286
Mouse monoclonal anti-β-actin [AC-15] (WB 1:10,000)	Sigma-Aldrich	A5441
Rabbit polyclonal anti-Rad51 [H-92] (WB 1:1000)	Santa Cruz Bio	sc-8349
Rat monoclonal anti-GFP [3H9] (WB 1:1000)	Chromotek	3h9
Rabbit polyclonal anti-Fe65 (WB 1:2000)	Abcam	ab5668
Mouse monoclonal anti-c-Myc [9E10] (WB 1:1000)	Santa Cruz Bio	sc-40
Rabbit monoclonal anti-Histone H4 (acetyl K16) [EPR1004] (WB 1:1000)	Abcam	ab109463
Rabbit polyclonal anti-Histone H3 (WB 1:30,000)	Abcam	ab1791
Rabbit polyclonal anti-PARP1 (WB 1:3000)	Enzo Life Sciences	ALX-210-895
Rabbit polyclonal anti-PARP1 (IF 1:1000)	GeneTex	GTX100573
Rabbit polyclonal anti-Ki67 (IHC 1:1000)	Abcam	ab15580
Rabbit polyclonal anti-Cleaved Caspase-3 (Asp175) (IF 1:300)	Cell Signaling Technology	9661
Sheep anti-mouse IgG, HRP-linked (WB 1:10,000)	Amersham	NXA931

Goat Anti-Rabbit IgG (H+L) Peroxidase AffiniPure (WB 1:20,000)	Jackson ImmunoResearch	111–035-003
Goat Anti-Rat IgG (H&L) [HRP] (WB 1:10,000)	GenScript	A00167
Donkey anti-rabbit Alexa Fluor®488 (IF 1:500)	Invitrogen	A21206
Goat anti-rabbit IgG (H&L) Alexa Fluor® 647 (IF 1:300)	Abcam	ab150079
Streptavidin (HRP) (WB 1:5000)	Abcam	ab7403
**Bacterial and Virus Strains**		
DH5α Competent Cells	Thermo Scientific™	EC0112
ElectroMax Stbl4	Invitrogen™	11635018
**Biological Samples**		
Human Paraffin-embedded Tissue arrays of breast cancer	US Biomax, Inc.	BR1005b; BR1008b; BR2082c
**Chemicals and Recombinant Proteins**		
PARPi (KU-0059436; Olaparib)	Selleckchem	S1060
ATMi (KU-55933)	Sigma-Aldrich	5.31978
Cisplatin	Selleckchem	S1166
VP16	Sigma-Aldrich	E1383
Caffeine	Sigma-Aldrich	C8961
Cycloheximide (CHX)	Sigma-Aldrich	C7698
Ethidium bromide (EtBr)	Hylabs	BP451
Crystal Violet	Sigma-Aldrich	32675
polyethylenimine (PEI)	Polysciences	343-6484
Hexadimethrine bromide (Polybrene)	Sigma-Aldrich	H9268
EGF	Peprotech	AF-100-15
cholera toxin from vibrio cholerae	Sigma-Aldrich	C8052
Insulin, human recombinant	Biological Industries	41-975-100

Corning® Matrigel® Matrix High Concentration (HC), Growth Factor Reduced (GFR)	Corning	354263
Blasticidin	Invivogen	ANT-BL
Puromycin	invivogen	ant-pr
Dexamethasone (Dex)	Sigma-Aldrich	D4902
Biotin Phenol	Sigma-Aldrich	SML2135
**Critical Commercial Assays**		
WesternBright Quantom (ECL)	Advansta	K-12042
CellTiter 96® AQueous One Solution Cell Proliferation Assay (MTS)	PROMEGA	G3580
DAB peroxidase kit	Vector Labaratories	SK-4100
Lipofectamine RNAiMax	invitrogen	13778
TRIzol Reagent	Invitrogen	15596026
qScript cDNA synthetsis kit	Quanta Bio	95047
Fast SYBR green master mix	Applied Biosystems	4385610
GFP-Trap Magnetic Agarose Beads	Chromotek	gtma
Streptavidin MagBeads	GenScript	L00424
Phusion® High-Fidelity DNA Polymerase	NEB	M0530
Red Load Taq Master	Jena Bioscience	PCR-108
VECTASHIELD Antifade Mounting Medium with DAPI	Vector Labaratories	H-1200
Dako's Fluorescence Mounting Medium	Agilent	S3032
**Software and Algorithms**		
GraphPad Prism (v8.0)	GraphPad software lnc.	https://www.graphpad.com

ImageJ software (v1.8.0)	National Institutes of Health	https://imagej.nih.gov/ij/
IN Cell Investigator (v1.3)	GE Healthcare	https://www.cytivalifesciences.com/en/us

**Plasmids**		
pSpCas9(BB)-2A-GFP(PX458)	Addgene	#48138
pSpCas9N(BB)-2A-GFP(PX461)	Addgene	#48140
pSpgRNA	Addgene	#47108
pEGFP-C1	Clontech Laboratories	#6084
pDsRED-monomer-C1	Clontech Laboratories	632466
pLKO.1 - TRC cloning vector	Addgene	#10878
PLKO.1-TRC-RBM6-shRNA #1	Sigma-Aldrich	TRCN0000154498
PLKO.1-TRC-RBM6-shRNA #2	Sigma-Aldrich	TRCN0000152100
Tet-pLKO-puro	Addgene	#21915
Lenti-Cas9-2A-Blast	Addgene	#73310
pMD2.G	Addgene	#12259
psPAX2	Addgene	#12260
pCI-Fe65-myc	(10)	N/A
pcDNA3.1(+)	Invitrogen	V79020
pBS-II-KS(+)	Stratagene	212207
pCDNA3-APEX2-NES	Addgene	#49386
pX330-LMNA-gRNA1	(11)	N/A
pCR2.1-CloverLamin	(11)	N/A

**Plasmids generated in this study**		
**Plasmid**	**Vector backbone**	**Insert**
pSpCas9(BB)-2A-GFP(PX458)-RBM6-gRNA 244	pSpCas9(BB)-2A-GFP(PX458) digested with BbsI	phosphorylated and annelad primers F1,R1
pSpCas9(BB)-2A-GFP(PX458)-RBM6-gRNA 238	pSpCas9(BB)-2A-GFP(PX458) digested with BbsI	phosphorylated and annelad primers F2,R2
pSpCas9N(BB)-2A-GFP(PX461)-RBM6-Ex 3	pSpCas9N(BB)-2A-GFP(PX461) digested with BbsI	phosphorylated and annelad primers F3,R3
pSPgRNA-RBM6-EX 3	pSpgRNA digested with BbsI	phosphorylated and annelad primers F4,R4

pSpCas9(BB)-2A-GFP(PX458)-RBM6-gRNA-KI (For APEX2 KI)	pSpCas9(BB)-2A-GFP(PX458) digested with BbsI	phosphorylated and annelad primers F5,R5
pEGFP-C1-RBM6-WT	pEGFP-C1 digested with XmaI,SalI	PCR product containing RBM6 coding sequence was amplified from cDNA using primers F6,R6 and digested with AgeI,SalI
pEGFP-C1-RBM6-delRRM	pEGFP-C1-RBM6	All round PCR using primers F7,R7
pDsRed-monomer-C1-RBM6 wt	pDsRED-monomer-C1 digested with EcoRI, MluI	pEGFP-C1-RBM6 digested with EcoRI, MluI, ApaLI
pDsRed-monomer-C1-RBM6 delRRM	pDsRED-monomer-C1 digested with EcoRI, MluI	pEGFP-C1-RBM6-delRRM digested with EcoRI, MluI, ApaLI
pDsRed-monomer-C1-myc-Fe65	pDsRed-monomer-C1 digested with XbaI, BamHI	PCR product from pCI-Fe65-myc using primers F8,R8 and digested with BamHI, NheI
pLV-Fe65-T2A-Blast	Lenti-Cas9-2A-Blast digested with BamHI, NheI	PCR product from pCI-Fe65-myc using primers F8,R8 and digested with BamHI, NheI
pLV-RBM6-T2A-Blast	Lenti-Cas9-2A-Blast digested with BamHI, NheI - 5' overhangs filled in	PCR product from pEGFP-C1-RBM6-WT using primers F9,R9
PLKO.1-TRC-Scramble	pLKO.1 - TRC cloning vector digested with EcoRI, AgeI	Annealed primers F11,R11
PLKO.1-TRC-RNPS1-shRNA#1	pLKO.1 - TRC cloning vector digested with EcoRI, AgeI	Annealed primers F12,R12
PLKO.1-TRC-RNPS1-shRNA#2	pLKO.1 - TRC cloning vector digested with EcoRI, AgeI	Annealed primers F10,R10
pLenti-IRES-GFP-RBM6 WT	pLenti-IRES-GFP digested with AgeI, PstI	PCR product from pEGFP-C1-RBM6-WT using primers F13,R13 and digested with AgeI, SbfI
pBS-LA-RBM6	pBS-II-KS(+) digested with KpnI, SalI	PCR on genomic DNA extracted from MCF10A cells using primers F14,R14 and digested with KpnI, SalI
pBS-LA-RA-RBM6	pBS-LA-RBM6 digested with BamHI, SacI	PCR on genomic DNA extracted from MCF10A

		cells using primers F15,R15 and digested with BamHI, SacI
pCDNA3 Puro-P2A-APEX2	pCDNA3-APEX2-NES digested with NdeI, NotI	PCR on Tet-PLKO-puro using primers F16,R16 and digested with NdeI, NotI
pBS-LA-Puro-P2A-Flag-APEX2-linker-RA-RBM6	pBS-LA-RA-RBM6 digested with SalI, BamHI	PCR on pCDNA3 Puro-P2A-APEX2 using primers F17,R17 and digested with SalI, BamHI
pcDNA3.1-6xmyc-RNPS1	pcDNA3.1(+) digested with XhoI, ApaI	PCR product containing RNPS1 coding sequence was amplified from cDNA using primers F18,R18 and digested with ApaI,SalI

**Cloning Primers**	
**Primer**	**Sequence**
F1	CACCGCCTCCTCGATAGTCACCATG
R1	AAACCATGGTGACTATCGAGGAGGC
F2	CACCGGGACCGCATGGTGACTATCG
R2	AAACCGATAGTCACCATGCGGTCCC
F3	CACCGCCACCCCTATAGTCCATAGG
R3	AAACCCTATGGACTATAGGGGTGGC
F4	CACCGTATGGATTATAGAGGTAGGG
R4	AAACCCCTACCTCTATAATCCATAC
F5	CACCGTTGATAAAAAGAGATGTGGG
R5	AAACCCCACATCTCTTTTTATCAAC
F6	AACGTCGACATGTGGGGGGATTCTCGACCTGCTAAC
R6	GCGACCGGTTTAATCGAGTTCTTTATATCGAGCAAACAT
F7	AACCTGGCCACTGGAAAACGAAGA
R7	AAGCCTGCTGGGTTTCTCCTCACTTGG
F8	GCAGGATCCATGTCTGTTCCATCATCACTGAGCCAGT
R8	CGTGCTAGCCTCAATTCAAGTCCTCTTCAGAAATG
F9	AAGACCGGTATGTGGGGGGATTCTCGACCTGCTAAC
R9	CCGCCTGCAGGTTAATCGAGTTCTTTATATCGAGCAAAC
F10	CCGGCAGCTCCAACTCCTCCCGATACTCGAGTATCGGGAGGAGTTGGAGCTGTTTTTG
R10	AATTCAAAAACAGCTCCAACTCCTCCCGATACTCGAGTATCGGGAGGAGTTGGAGCTG

F11	CCGGGTGGACTCTTGAAAGTACTATCTCGAGATTTGACGGGTGG ATAATCTGTTTTT
R11	AATTAAAAACAGATTATCCACCCGTCAAATCTCGAGATAGTACT TTCAAGAGTCCAC
F12	CCGGCGTAGAGTTTGAGAATCCAGACTCGAGTCTGGATTCTCAA ACTCTACGTTTTTG
R12	AATTCAAAAACGTAGAGTTTGAGAATCCAGACTCGAGTCTGGAT TCTCAAACTCTACG
F13	AAGACCGGTATGTGGGGGGATTCTCGACCTGCTAAC
R13	CCGCCTGCAGGTTAATCGAGTTCTTTATATCGAGCAAAC
F14	AACGGTACCACTGCTTGAGCTCAGGAGTTTGAGACCAG
R14	GGCGTCGACCTCTTTTTATCAAGAGGGCCCCAACAAGTA
F15	ATAGGATCCTGGGGGGATTCTCGACCTGCTAACAGAAC
R15	AATCCGCGGGCCTGTAATCCCAGCTACTCAGAAGGCTG
F16	GGCCATATGATGGCTACCGAGTACAAGCCCACGG
R16	ATAGCGGCCGCAGGTCCAGGGTTCTCCTCCACGTCTCCAGCCTGC TTCAGCAGGCTGAAGTTAGTAGCGGCACCGGGCTTGCGGGTCAT G
F17	AATGGATCCAGATCCAGATCCACCACCTCCTGAGCCTCCACCTCC TCCCGGGGCATCAGCAAACCCAAGCTCGGAAAGC
R17	ATAGTCGACATGGCTACCGAGTACAAGCCCACGG
F18	GCGGTCGACATGGATTTATCAGGAGTGAAAAAGAAGAGC
R18	ATAGGGCCCTTATCGGGAGGAGTTGGAGCTGGAGCGGCTC

PCR Primers

**Primer**	**Sequence**
Fe65-mRNA-Ex7_8-F	GGCTGGGGGGAAGGAAAGGATCT
Fe65-mRNA-8-R	CTGGCTCTGTGGCTCCACTA
Fe65-Intron7-F	CAGAGATCAGCCACCATCCT
EYA2-RT-F	GCTGGTCACCACCACTCAAC
EYA2-RT-R	CCTCTCGAAGCAGCTCTCCT
PPP2R2C-RT-F	CCGACAGGAGCTTCAACATC
PPP2R2C-RT-R	CTGCTGCTGTAGACGAAGAG
Fe65- Ex14-F	TTCCTGGCCGTGGGCAGAGATGT
Fe65- Ex15-R	CACTTCTGGTAGCGAAGCAT
Fe65-NSD-A3SS-R	CCTGTTCCACCATGCACGCAGCCT
PELO-RT-F	CGGTCCAAATTTCTTCAGGTACA
PELO-RT-R	AGAAAGCTCGATCCGGTTCA
Fe65-Ex3-F1	GTGCAGCCACCTTGGCGTAA
Fe65-Ex3-R1	GACTGGTCCAAGAGGCAGTA
RNPS1-RT-F	ATCTGTCCAAAGGCTATGCG

RNPS1-RT-R	TCTCCTGGCCATCAATTTGT
Rad51-RT-F	CAATGCAGATGCAGCTTGAA
Rad51-RT-R	CCTTGGCTTCACTAATTCCCT
Rad54B-RT-F	TCCAGGTCTGAATGAAGAGATTAC
Rad54B-RT-R	TCTAGTACTTTCTTCACTAGGCAG
XRCC2-RT-F	TCACCTGTGCATGGTGATATT
XRCC2-RT-R	TTCCAGGCCACCTTCTGATT
GAPDH-RT-F	CCAGGGCTGCTTTTAACTCT
GAPDH-RT-R	GGTGCCATGGAATTTGCCAT

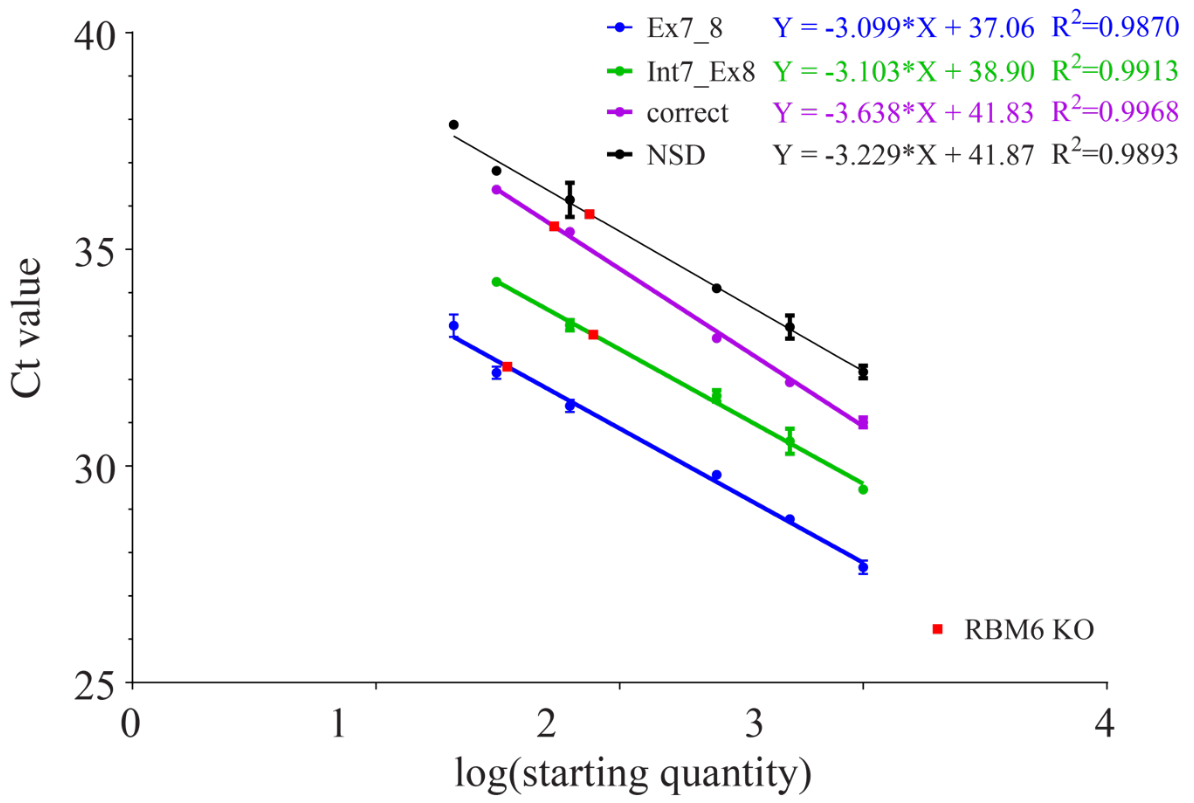

Appendix 1
cDNA from MCF10A-HRas cells was subjected to serial dilutions before qPCR analysis in the Step-One-Plus real-time PCR System (Applied Biosystems) using the indicated primers and the Fast SYBR Green Master mix (Applied Biosystems) with three technical repeats for each PCR. The Ct values for each serial dilution was plotted against the relative starting-quantity and linear range and fitness was determined to be acceptable. The Ct value obtained from MCF10A RBM6-KO1 samples (consistently the lowest values obtained in any experiment) are indicated on the graphs and fall within the linear range.
